# Flexible neural control of transition points within the egg-laying behavioral sequence in *Drosophila*

**DOI:** 10.1038/s41593-023-01332-5

**Published:** 2023-05-22

**Authors:** Kevin M. Cury, Richard Axel

**Affiliations:** 1 The Mortimer B. Zuckerman Mind Brain Behavior Institute, Department of Neuroscience, Columbia University, New York, NY USA; 2 Howard Hughes Medical Institute, Columbia University, New York, NY USA

**Keywords:** Decision, Motor control, Sensory processing, Sexual behaviour

## Abstract

Innate behaviors are frequently comprised of ordered sequences of component actions that progress to satisfy essential drives. Progression is governed by specialized sensory cues that induce transitions between components within the appropriate context. Here we have characterized the structure of the egg-laying behavioral sequence in *Drosophila* and found significant variability in the transitions between component actions that affords the organism an adaptive flexibility. We identified distinct classes of interoceptive and exteroceptive sensory neurons that control the timing and direction of transitions between the terminal components of the sequence. We also identified a pair of motor neurons that enact the final transition to egg expulsion. These results provide a logic for the organization of innate behavior in which sensory information processed at critical junctures allows for flexible adjustments in component actions to satisfy drives across varied internal and external environments.

## Abstract (web summary)

Behaviors often consist of sequences of component actions. Cury and Axel identify distinct classes of sensory neurons that control the transitions between the components of egg laying in the fly that afford this behavior an adaptive flexibility.

## Main

Organisms have evolved a repertoire of innate behaviors, comprised of sequences of component actions, to satisfy essential drives^1–3^. Progression along an innate behavioral sequence is regulated by distinct stimuli, or ‘releasers’, to ensure that transitions between component actions occur in a suitable context at the appropriate time^3^. This mechanism imparts behavioral flexibility by introducing decision points that allow innate behaviors to adapt to variation in the organism’s internal and external environment. Control at the junctures of component actions is a fundamental property of many instinctive behaviors.

The drive to reproduce is a dominant motivator of behavior in all species. Diverse behavioral programs dedicated to courtship, copulation and the production and care of offspring have evolved to optimize reproductive success. For oviparous animals that do not brood, such as the fruit fly *Drosophila melanogaster*, egg deposition represents the culmination of this array of reproductive behaviors. Considerable pressure is imposed on the selection of the appropriate time and place to deposit eggs. Fruit flies express strong, species-specific preferences for the site of egg deposition based, in part, on odor, taste, texture and the spatial dimension of the environment^4–12^. During egg laying, females evaluate the local environment before expressing an ordered motor sequence (abdominal bending, ovipositor (hypogynium) burrowing and egg expulsion) that culminates in egg deposition subterraneously within a nutritive substrate^10,13–18^. After egg expulsion, the female comes to rest, this final phase of the behavioral sequence is coupled to ovulation and fertilization, and the cycle repeats.

Egg laying in the fly is initiated by a seminal fluid peptide, sex peptide, introduced into the uterus (genital chamber) during mating^19^. Sensory information from neurons responsive to sex peptide is relayed to the brain, inhibiting a subset of the pC1 cluster of neurons^15,20–23^. This disinhibits the oviposition descending neurons (oviDNs), a collection of descending interneurons that project to the ventral nerve cord and are necessary and causal for the expression of the ordered egg deposition motor sequence^15^. One model posits that ramping activity in these descending neurons determines the progression of this terminal motor sequence^15,24^. Progression along the sequence, however, is likely to be dictated by the ongoing acquisition of sensory information, allowing the motor pattern to adapt to variability in the internal and external environment.

In this study, we have characterized the detailed structure of egg-laying behavior and identify that the transitions between component actions are variable and can be flexibly adjusted to accommodate diverse environmental conditions. Moreover, we have identified three classes of neurons that control the timing and direction of specific transitions within the terminal egg deposition motor sequence. These results provide both a behavioral logic and a neural basis for the imposition of adaptive flexibility on an innate and stereotyped sequence of motor actions.

## Results

### Variable transitions in the egg-laying behavioral sequence

**Fig. 1:**
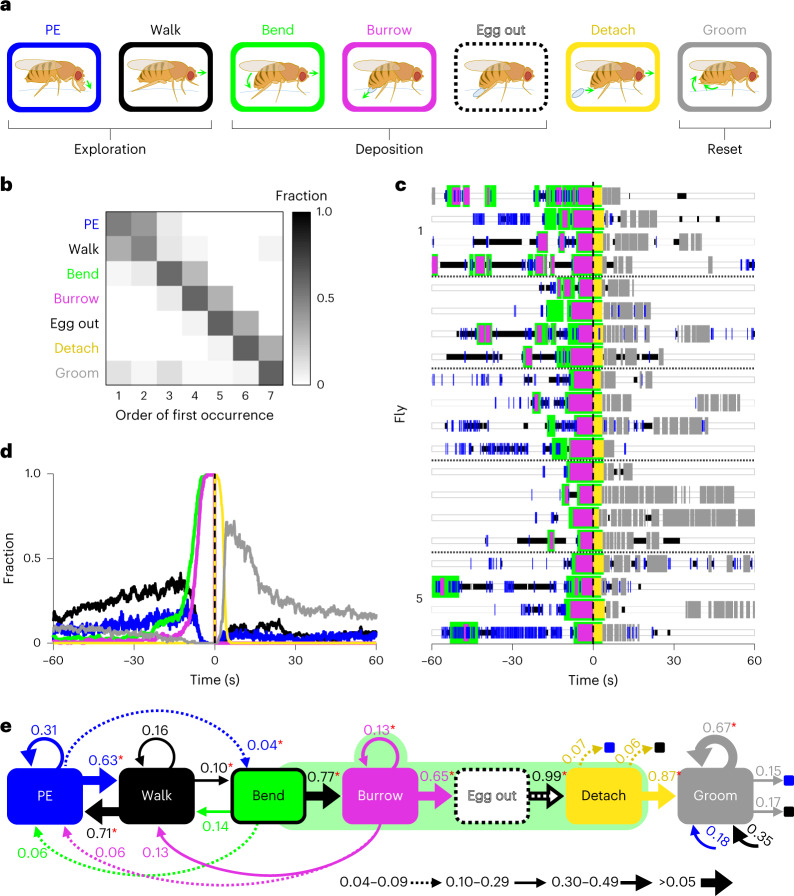
The egg-laying sequence exhibits variable transitions between component actions. **a**, Illustrations depicting component actions of the egg-laying behavioral sequence. Components comprising exploration, deposition and reset phases are indicated. The colors used in text and boxes here indicate component actions in all subsequent figures; PE, proboscis extension. **b**, Order of occurrence of the first instance of each behavioral component depicted as a fraction of total events. Only events including all components were analyzed (169 of 176 events). **c**, Representative ethograms of egg-laying behavior in five flies (*n* = 4 events per fly). Here and in **e**, bend is drawn wider to emphasize that this behavior is maintained throughout burrow, egg out and detach behaviors. Horizontal black dashed lines demarcate data from different flies. Here and in **d**, *t* = 0 marks the time of completed egg expulsion (egg out). **d**, Average time course of the seven annotated behaviors depicted as the instantaneous fraction of total events; *n* = 176 events from 18 flies. **e**, Diagram depicting the start-to-start transition probabilities between the seven annotated behaviors. An asterisk indicates transitions occurring significantly higher than chance (*P* < 0.001, one-sided permutation test; Methods and Supplementary Table 2). Transitions with probabilities less than 0.04 were not significant and were omitted from the diagram. Self-transitions indicate that the behavior started, stopped and started again without the initiation of any other intervening behavior.

**Extended Data Fig. 1:**
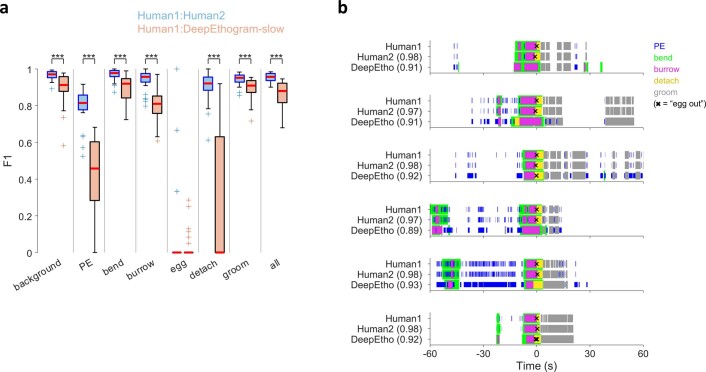
Human-Human agreement is high and exceeds supervised behavioral classifier output. **a**, Comparison of annotation agreement between two humans and between a human and a supervised behavioral classifier (DeepEthogram; Methods). n = 30 videos for all groups; same videos re-annotated by second human and held out as test dataset for supervised classifier. Blue, human-human agreement; pink, human-DeepEthogram agreement. Box bounds, 25^th^ and 75^th^ percentile; red line, median; whiskers, 5^th^ and 95^th^ percentile; +, outliers. background, unlabeled frames; all, all behaviors combined. ***p < 0.001, two-sided Wilcoxon rank sum test (Supplementary Table 7). **b**, Representative ethograms from six of 30 videos, displaying the annotations of two humans (top two rows of each plot) and the output of DeepEthogram (bottom row). Values within parenthesis at left, F1 score of the corresponding annotation compared with Human1 (top row annotation) for all behaviors combined. *t* = 0 marks the time of completed egg expulsion (egg out).

**Extended Data Fig. 2:**
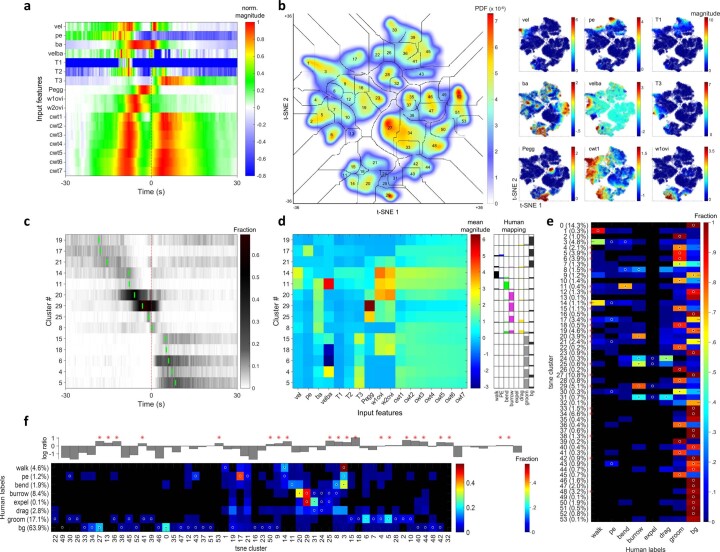
Reliable mapping between manual annotations and unsupervised behavioral classifier. **a**, Mean time course of the 17 extracted features used for unsupervised behavioral classification analysis, plotted surrounding egg-laying (input feature definitions in Methods), each normalized to a maximum of 1. Here and in **c**, *t* = 0, egg out. **b**, Left: probability density function (PDF) generated from t-SNE embedding. Boundary lines, watershed-transform segmented behavioral clusters, with cluster index indicated within. Right: feature magnitudes of embedded data points plotted for 9 of the 17 features. **c**, Time course of expression fraction of the 14 t-SNE clusters that were expressed significantly higher than chance (p < 0.001, one-sided Fisher’s exact test; see top plot in **f**) and displayed peak expression within ± 20 seconds of egg out. Clusters sorted according to their peak timing (green tick mark) here and in **d**. Examples of these 14 clusters shown in Supplementary Video 3. **d**, Left: mean feature magnitudes of embedded data points (columns) corresponding to each of the 14 t-SNE clusters shown in **c** (rows). Right: mapping of these 14 clusters onto human labels, displayed as a fraction (bar height indicates fraction from 0 to 1). Here and in **e** and **f**, bg, unlabeled, background frames. **e**, Mapping of t-SNE clusters (rows) onto human labels (columns), displayed as a fraction. Percentage of total frames within original embedded data points belonging to each cluster is indicated at left. Here and in **f**, mappings significantly higher than chance are indicated by a white-rimmed black circle (p < 0.001, one-sided permutation test; Supplementary Methods; Supplementary Table 8); red asterisks, clusters expressed significantly higher than chance surrounding egg laying (p < 0.001, one sided Fisher’s exact test; Supplementary Table 7); t-SNE cluster # 0 corresponds to stationary frames. **f**, Top: log ratio of t-SNE cluster expression fraction within ± 60 seconds surrounding egg-laying relative to expression fraction within original embedded data points. Here and below, clusters arranged according to their peak timing. Bottom: mapping of human labels (rows) onto t-SNE clusters (columns), displayed as a fraction. Percentage of total frames given a particular label indicated at left.

Females lay eggs one at a time in a repeating cycle, continually transitioning between three distinct phases of a behavioral sequence. Each egg-laying cycle is comprised of an active exploratory phase, deposition and a more stationary phase (‘reset’) that includes ovulation, after which the cycle repeats (Supplementary Fig. 1)^10,13,14,17,18^. We have studied the composition of this sequence in detail by filming individual gravid females at high resolution in small egg-laying chambers on a 1% agarose substrate and manually scoring the component behaviors (Supplementary Fig. 2, Supplementary Video 1 and Supplementary Table 1). Before deposition, flies explored the substrate with their proboscis and legs. During this phase, flies extended their proboscis to make brief contact with the substrate and walked to a new location (Fig. 1a). They then transitioned to deposition and bent their abdomen to bring the ovipositor in contact with the surface, initiated substrate burrowing (a rhythmic behavior in which the ovipositor digs into the substrate and expels the egg) and ultimately deposited the egg subterraneously. After egg expulsion, the flies abruptly stopped burrowing, detached from the egg and then lifted and groomed their ovipositor (Fig. 1a). The females then remained stationary for an extended period of time, intermittently grooming and exhibiting abdominal contortions likely to result from ovulation (the reset phase)^17^. The behavioral sequence then repeated. This ordering of component actions was highly conserved across repeated egg-laying events (Fig. 1b). We independently rescored a subset of data using a second human annotator to demonstrate the consistency and reproducibility of our manual labels. Human–human labeling agreement, as determined using the F1 scoring metric^25,26^, was above 90% for most behaviors and above 95% for all behaviors combined (Extended Data Fig. 1). We further validated these behavioral observations by implementing an unsupervised behavioral classification analysis based on a pose estimation model to automatically identify stereotyped, recurring behavioral actions^27,28^. There was high correspondence between the unsupervised classifier and our manually defined behavioral categories and labels (Supplementary Fig. 3, Extended Data Fig. 2, Supplementary Videos 2 and 3 and Methods). Thus, egg-laying behavior appears to be organized as an ordered sequence of behavioral components.

Although the sequential organization of these behaviors is conserved, the timing, frequency and duration of the individual components within this behavioral sequence exhibit considerable variability (Fig. 1c,d). Moreover, the behavioral sequence was conserved, but transitions could occur in both directions (Fig. 1e and Supplementary Table 2). This variability in transitions was not only apparent for exploration but also observed for deposition behaviors. Although burrowing was always preceded by abdominal bending, bending was not always followed by burrowing. Instead, walking or proboscis contact were observed. Likewise, 35% of burrowing episodes did not persist to egg expulsion but were aborted in favor of additional bouts of burrowing or further exploration. Persistent burrowing invariably preceded egg expulsion, and behavioral transitions following expulsion exhibited little variability and proceeded along the ordered sequence to the reset phase. Thus, during both exploration and deposition, the egg-laying sequence is comprised of multiple junctures between component actions that may serve as decision points. These junctures may allow the fly to advance or reinitiate the sequence contingent on sensory information obtained during a component action.

### Egg deposition sequence adjusts to changes in substrate firmness

**Fig. 2:**
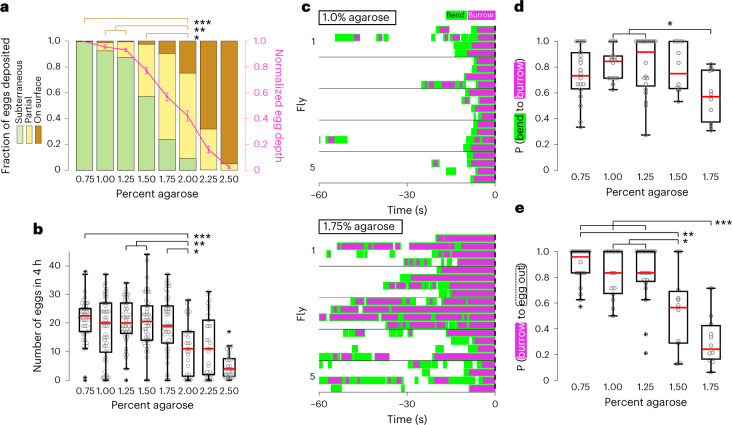
Egg deposition sequence adjusts to changes in substrate firmness. **a**, Left ordinate: distributions of the depth of eggs released on substrates of varying firmness; partial, partially subterraneous. The mean fraction of all eggs pooled per group is presented (here and in **b**; *n* = 48, 54, 48, 48, 48, 32, 32 and 32 flies per group). Right ordinate: mean and s.e.m. of the per-fly average-normalized egg depth (magenta). The statistical test comparing the number of ‘on surface’ eggs released is depicted only for groups from 0.75% to 2.0% agarose. Here and in **b**, **d** and **e**, *P < 0.05, **P < 0.01 and ***P < 0.001, as determined by a Kruskal–Wallis test with post hoc Tukey’s honestly significant difference (HSD) test (Supplementary Table 7). **b**, Number of eggs released on substrates of varying firmness in 4 h. Here and in **d** and **e**, box bounds indicate the 25th and 75th percentiles, the red lines indicate the medians, and the whiskers indicate the 5th and 95th percentiles; o, data from individual flies; +, outliers. The statistical test is depicted only for groups from 0.75% to 2.0% agarose. **c**, Representative ethograms depicting bending and burrowing behavior in five flies on 1.0% agarose (top) and 1.75% agarose (bottom); *n* = 4 events per fly; *t* = 0, egg out. **d**, Average probability (P) of progression from bending to burrowing across substrates of varying firmness. Only flies that exhibited three or more bend bouts are considered (*n* = 19, 15, 21, 11 and 11 flies per group). **e**, Average probability of progression from burrowing to completed egg expulsion (egg out). Only flies that exhibited three or more burrowing episodes are considered (*n* = 19, 15, 21, 10 and 11 flies per group).

We therefore asked whether abdominal bending and ovipositor burrowing, component actions obligatory for the subterraneous deposition of the egg, adapt to changes in the properties of the substrate. We initially scored both the count and the depth of penetration of eggs laid on agarose substrates of increasing firmness (agarose concentrations from 0.75% to 2.5%; Fig. 2a,b)^7,11,16^. As substrate firmness was increased, flies were less successful at achieving subterraneous egg deposition (Fig. 2a). Above 1.75% agarose, total egg output dropped significantly, with a mean of 11 eggs laid in 4 h on 2.0% agarose compared to 18–21 eggs on 0.75% to 1.75% agarose (Fig. 2b), and flies deposited a significantly larger fraction of eggs on the substrate surface (Fig. 2a). These data suggest that the ability to achieve subterraneous egg placement is sensitive to substrate firmness and may positively gate egg output.

We filmed egg-laying behavior on different substrates and observed that progression along the deposition sequence was dramatically reduced as the firmness of the agarose substrate was increased (Fig. 2c). The probability of transitioning from bending to burrowing was highest on 1% agarose and was reduced by 29% on 1.75% agarose (Fig. 2d). Moreover, burrowing-to-expulsion transitions reduced by 65% as the agarose concentration increased from 0.75% to 1.75% (Fig. 2e). These data suggest that abdominal bending is not simply a means of initiating burrowing. Rather, bending may allow substrate sampling by sensory organs on the abdominal terminalia that permits the recognition of tactile cues that regulate the transition to burrowing. Burrowing is also likely to be gated by tactile feedback as the fly makes contact with and engages the substrate in an effort to achieve subterraneous egg deposition. Burrowing may be unsuccessful on firmer substrates and can be aborted in search of a more favorable location. The variability in the sequence of these behaviors is likely to reflect the search for an optimal location to deposit eggs subterraneously.

### Terminalia sensory bristles regulate sequence progression

**Fig. 3:**
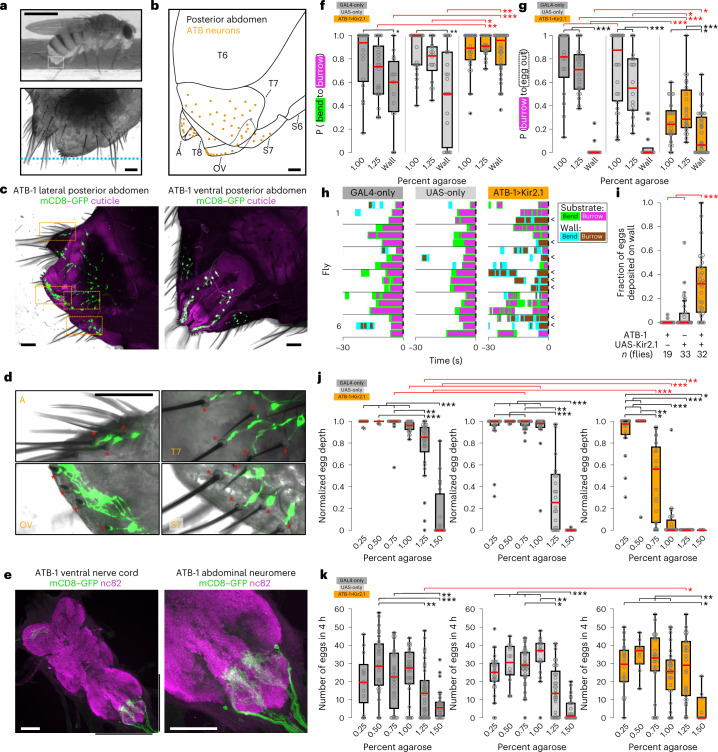
Terminalia sensory bristles regulate sequence progression. **a**, Top: example video snapshot of abdominal bending. Scale bar, 1 mm. Bottom: brightfield image of the female posterior abdomen, approximating terminalia bristle surface contact (white box at top). The dashed blue line indicates the approximate substrate surface. Scale bar, 50 μm (**b**–**e**). **b**, Diagram of the female posterior abdomen (lateral aspect); orange circles, bristles innervated by GFP-labeled neurons in the representative ATB-1>mCD8–GFP image in **c** (left); gray circles, non-innervated bristles; T6–T8, sixth–eighth tergite; S6 and S7, sixth and seventh sternite; A, analia; OV, ovipositor valve. **c**, Representative images of the posterior abdomen from two of nine ATB-1>mCD8–GFP females (lateral (left) and ventral (right) aspects); mCD8–GFP expression, membrane of ATB neurons (green); autofluorescence, abdominal cuticle (magenta); background, overlaid brightfield images revealing extended bristles; orange boxes, regions shown in **d**. **d**, Higher-resolution regions of the left image in **c** displaying GFP-labeled and brightfield images. Red asterisks indicate bristles innervated by single GFP-labeled neurons. **e**, Representative image of the ventral nerve cord (left) and abdominal neuromere (right) from three ATB-1>mCD8–GFP females stained with anti-GFP (ATB neurons, green) and anti-bruchpilot (nc82; synaptic neuropil, magenta). Black bars flanking the left image indicate the region shown at higher resolution on the right. **f**, Average probability (P) of progression from bending to burrowing. Only flies that exhibited two or more bend bouts were considered (*n* = 17, 22, 15, 27, 17, 23, 25, 24 and 36 flies per group). Here and in **g** and **i**–**k**, box bounds indicate the 25th and 75th percentiles, the red line indicates the medians, and the whiskers indicate the 5th and 95th percentiles; o, data from individual flies; +, outliers. Here and in **g** and **i**, *P < 0.05, **P < 0.01 and ***P < 0.001; data were analyzed by two-sided Wilcoxon rank-sum test followed by a Bonferroni correction (Supplementary Table 7). **g**, Average probability of progression from burrowing to egg out. Only flies that exhibited two or more burrowing episodes were considered (*n* = 17, 22, 15, 27, 17, 17, 25, 24 and 41 flies per group). **h**, Representative ethograms depicting bending and burrowing behavior on 1.0% agarose. Each ethogram depicts data from five flies (*n* = 3 events per fly); <, eggs deposited on the wall; *t* = 0, egg out. **i**, Fraction of eggs deposited on walls of chambers containing 1% agarose substrate (*n* = 19, 33 and 32 flies per group). Only flies that released three or more eggs were considered. **j**, Average normalized depth of penetration of eggs released on substrates of varying firmness (GAL4-only, *n* = 10, 27, 21, 25, 29 and 19 flies per group; UAS-only, 29, 13, 34, 18, 24 and 12 flies per group; ATB-1>Kir2.1, 19, 10, 22, 24, 25 and 4 flies per group). Here and in **k**, *P < 0.05, **P < 0.01 and ***P< 0.001; data were analyzed by Kruskal–Wallis test with a post hoc Tukey’s HSD test (Supplementary Table 7). **k**, Number of eggs released in 4 h on substrates of varying firmness (GAL4-only, *n* = 13, 29, 28, 30, 42 and 28 flies per group; UAS-only, 31, 13, 35, 19, 33 and 27 flies per group; ATB-1>Kir2.1, 20, 10, 26, 27, 27 and 10 flies per group).

**Extended Data Fig. 3:**
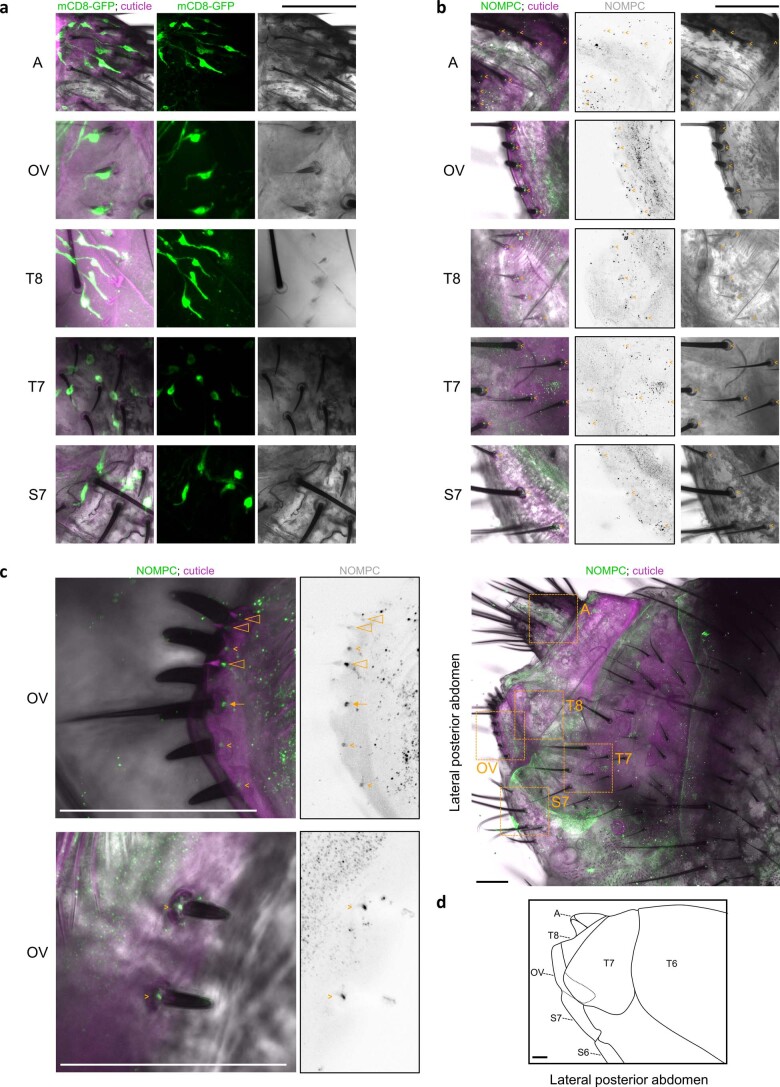
Mono-innervation and anti-NOMPC labeling of terminalia bristles. **a**, Representative images of posterior abdomen from one of four pan-neuronal elav-GAL4>mCD8–GFP females. Left and middle: mCD8-GFP, pan-neuronal expression demonstrating the innervation of each bristle by the distal process of a single bipolar neuron (green). The innervation patterns associated with the long sensillum and short sensilla of the ovipositor (hypogynium) could not be deciphered. Left: autofluorescence, abdominal cuticle (magenta). Left and right: bright-field (grayscale). Here and in **b**-**d**, A, analia; OV, ovipositor valves; T8, 8^th^ abdominal tergite; T7, 7^th^ abdominal tergite; S7, 7^th^ abdominal sternite; scale bar, 50 μm. **b**, Top five rows: representative images of posterior abdomen from one of nine wild-type female flies stained with anti-NOMPC (green at left, grayscale at middle). Left: autofluorescence, abdominal cuticle (magenta). Left and right: bright-field (grayscale). Arrowheads, foci of anti-NOMPC labeling observed at the base of all bristles^34^. Bottom: lower resolution image of the posterior abdomen, lateral aspect, containing the regions displayed above (orange boxes). **c**, Representative images of the ovipositor valves from two of nine wild-type flies stained with anti-NOMPC (green at left, grayscale at right). Left, autofluorescence, abdominal cuticle (magenta); bright-field (grayscale). Foci of anti-NOMPC labeling at the base of the three hypogynial short sensilla, the singular long sensillum, and the hypogynial teeth are indicated by triangles, the arrow, and the arrowheads, respectively. **d**, Diagram of female posterior abdomen, lateral aspect.

Peripheral touch sensation in *Drosophila* is mediated by tactile hairs, or bristles, that cover the surface of the fly^29^. The bristles of the ovipositor valves and adjacent segments of the abdominal terminalia make contact with the substrate during egg deposition in *Drosophila* (Fig. 3a)^30–32^. In flies that express pan-neuronal green fluorescent protein (GFP), we observed that the vast majority of terminalia bristles are innervated by a single bipolar neuron, a canonical feature of purely mechanosensory bristles (elav-GAL4>mCD8–GFP; Extended Data Fig. 3a)^29,31^. Moreover, the base of all terminalia bristles stains with an antibody directed to NOMPC, a force-sensitive ion channel present in mechanosensory neurons (Extended Data Fig. 3b,c)^33–35^. These observations suggest that the terminalia bristles harbor mechanosensory neurons that play a role in tactile sensing during the egg deposition sequence.

**Extended Data Fig. 4:**
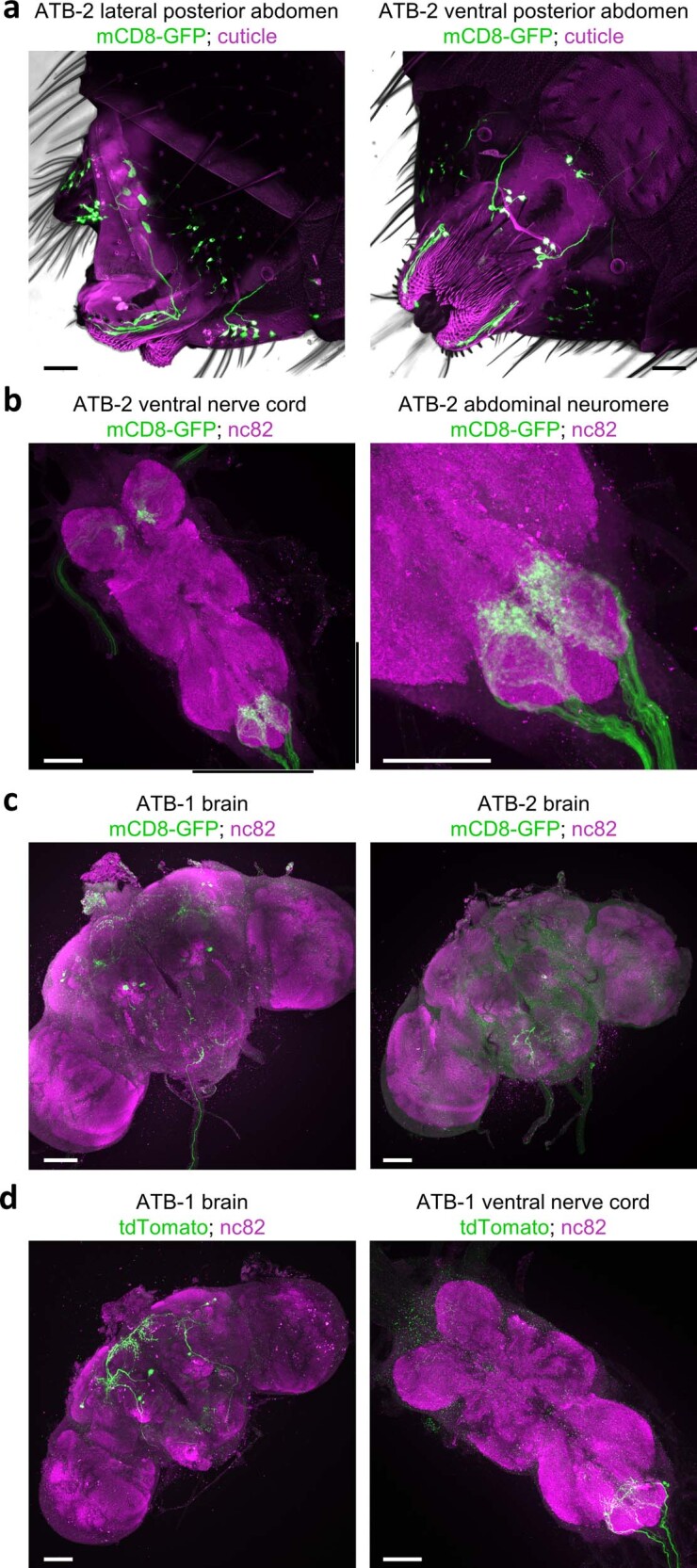
Expression pattern of ATB-split-GAL4 lines. E **a**, Representative images of the posterior abdomen from two of seven ATB-2>mCD8-GFP females, lateral (left) and ventral aspects (right). mCD8-GFP expression, membrane of ATB neurons (green); autofluorescence, abdominal cuticle (magenta). Background, overlaid bright-field images reveal extended bristles. Here and in **b**-**d**, scale bar, 50 μm. **b**, Representative image of the ventral nerve cord (left) and abdominal neuromere (right) from two ATB-2>mCD8-GFP females, stained with anti-GFP (membrane of ATB neurons, green) and nc82 (synaptic neuropil, magenta). Black bars flanking left image, region shown in higher resolution at right. **c**, Representative images of the brain from one of two ATB-1>mCD8-GFP females (left) and from one of two ATB-2>mCD8-GFP females (right), stained with anti-GFP (membrane of ATB neurons, green) and nc82 (synaptic neuropil, magenta). **d**, Representative images of the brain (left, one of six flies) and ventral nerve cord (right, one of six flies) from ATB-1>Kir2.1-T2A-tdTomato females, stained with anti-DsRed (ATB neurons co-expressing tdTomato and Kir2.1, green) and nc82 (synaptic neuropil, magenta). Foreleg expression is sparse in these flies (0.67 + /− 0.65 efferents per side in the dorsal prothoracic nerve; n = 12 nerves from six flies; mean ± s.d.).

We searched literature and image databases and used the split-GAL4 intersectional strategy to generate two restrictive driver lines (ATB-1 and ATB-2) that target the sensory neurons that innervate the abdominal terminalia bristles (ATB neurons; Fig. 3b–e, Extended Data Fig. 4 and Supplementary Table 3)^36–39^. ATB-1 also drives expression in a sparse population of neurons in the brain, whereas ATB-2 drives reliable expression in the forelegs but not in the brain. However, the only consistently labeled neurons common to these two lines are those that innervate the approximately 150 terminalia bristles (88% and 76% innervated overall, including 81% and 79% of ovipositor bristles, in ATB-1 and ATB-2, respectively; Supplementary Table 3). The axons of these neurons project to a ventral domain of the abdominal neuromere (Fig. 3e and Extended Data Fig. 4b) and are thus poised to inform local circuits about tactile properties of the substrate during egg deposition^18,40^.

**Extended Data Fig. 5:**
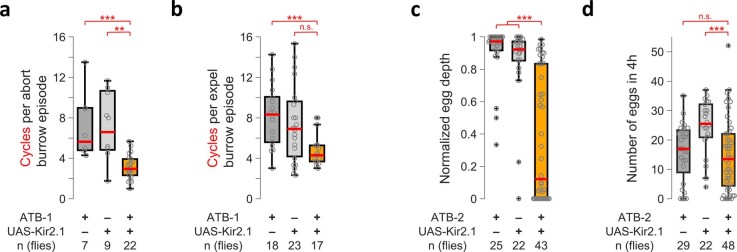
Aberrant egg-laying in ATB-1 silenced and ATB-2 silenced flies. **a**, Average number of cycles per aborted burrowing episode on a 1% agarose substrate. Only flies that exhibited two or more aborted burrowing episodes were considered here and in **b**. Burrowing episodes are comprised of discrete, rhythmic cycles (see Fig. 4); the cycle count per burrowing episode was highly positively correlated with the duration of the episode (r = 0.91). Here and in **b**-**d**, box bounds, 25^th^ and 75^th^ percentile; red line, median; whiskers, 5^th^ and 95^th^ percentile; o, data from individual flies; +, outliers; **p < 0.01, ***p < 0.001, n.s., p > .05, two-sided Wilcoxon rank sum test followed by Bonferroni correction (Supplementary Table 7). **b**, Average number of cycles per egg-expulsion burrowing episode on a 1% agarose substrate. **c**, Average normalized depth of penetration of eggs released on a 1% agarose substrate. Silencing neurons using either ATB-1 or ATB-2 splitGAL4 yielded significant deficits in subterraneous egg deposition (see also Fig. 3j, right panel), implicating a defect in the common set of terminalia bristle-innervating neurons in producing this phenotype. **d**, Number of eggs released on a 1% agarose substrate in 4 h.

We asked whether ATB neurons are functionally involved in egg laying by expressing the potassium channel Kir2.1 in these neurons to inhibit their activity (ATB-1>Kir2.1; Extended Data Fig. 4d)^41^. We initially filmed egg-laying behavior on 1% and 1.25% agarose substrates in control and ATB-silenced flies. Control flies exhibited reduced progression from bending to burrowing as we increased the firmness of the substrate (Fig. 3f). By contrast, ATB-silenced flies progressed from bending to burrowing with high probability on all substrates examined (Fig. 3f). These flies also showed aberrant burrowing behavior on agarose substrates; burrowing episodes were shorter in duration and more frequently aborted than in control flies (Fig. 3g,h and Extended Data Fig. 5a,b). Furthermore, ATB-silenced flies atypically deposited eggs on the rigid chamber walls; when burrowing on the wall, ATB-silenced flies expeled eggs during 15% of the burrowing episodes, whereas burrowing on the wall in control flies rarely persisted to egg expulsion (Fig. 3g–i).

Together, these results suggest that tactile feedback from ATB neurons modulates the egg deposition sequence, affording an adaptive response to the firmness of the substrate. While bending on a firm substrate, the tactile response of ATB neurons may suppress the transition to burrowing (Fig. 3f). During burrowing, tactile feedback from the ATB neurons may promote the persistence of burrowing on ideal substrates and elicit the abortion of burrowing on inappropriate substrates (Fig. 3g). Absent this feedback, bending transitions to burrowing with high frequency, and burrowing transitions to egg expulsion with low frequency, independent of the substrate firmness (Fig. 3f,g).

**Extended Data Fig. 6:**
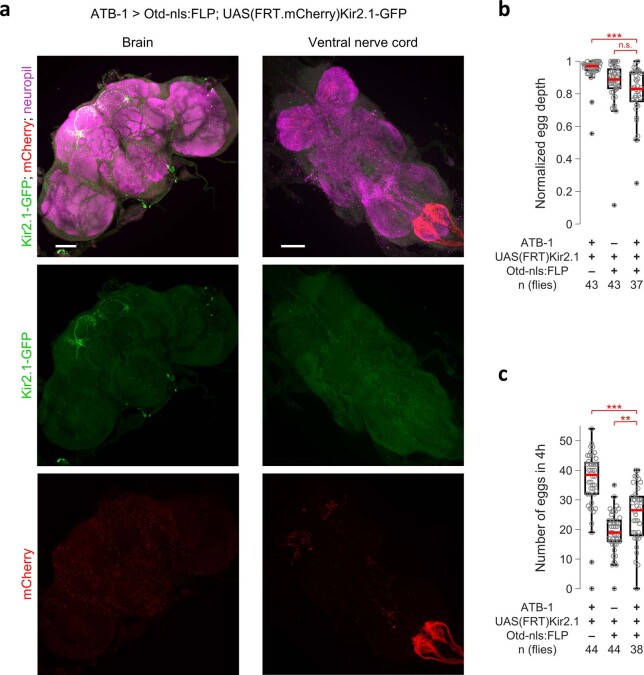
Silencing ATB-1 brain neurons does not result in deficit in subterraneous egg deposition. **a**, Representative images of the brain (left column, one of 13 flies) and ventral nerve cord (right column, one of four flies) from ATB-1>Otd-nls:FLP; UAS(FRT.mCherry)Kir2.1-GFP females. Flippase under control of the head-restricted Otd promotor used in combination with UAS(FRT.mCherry)Kir2.1-GFP results in the restricted expression of Kir2.1-GFP in ATB-1 brain neurons, whereas mCherry is expressed in ATB-1 non-brain neurons^42^. Samples stained with anti-GFP (Kir2.1-GFP-expressing ATB-1 neurons,green), anti-DsRed (mCherry-expressing ATB-1 neurons, red), and nc82 (synaptic neuropil, magenta). Scale bar, 50 μm. **b**, Average normalized depth of penetration of eggs released on a 1% agarose substrate. Subterraneous egg deposition is largely unaffected compared to ATB-1>Kir2.1 flies (see Fig. 3j, right panel). Here and in **c**, box bounds, 25^th^ and 75^th^ percentile; red line, median; whiskers, 5^th^ and 95^th^ percentile; “o”, data from individual flies; “+”, outliers; ***p < 0.001, **p < 0.01, n.s., p > .05, two-sided Wilcoxon rank sum test followed by Bonferroni correction (Supplementary Table 7). **c**, Number of eggs released on a 1% agarose substrate in a 4-hour window.

We next examined the consequences of ATB silencing on both the count and the depth of penetration of eggs across a wider range of substrate firmness. Flies with silenced ATB neurons exhibit a diminished ability to achieve subterraneous egg deposition on substrates firmer than 0.5% agarose, whereas control flies deposit subterraneously until 1.25% (Fig. 3j and Extended Data Fig. 5c,d; ATB-2>Kir2.1 silenced flies on 1% agarose). ATB-silenced flies continued to release a large number of eggs on firmer substrates despite the failure to achieve subterraneous egg placement (Fig. 3j,k and Extended Data Fig. 5c,d). By contrast, control flies began to show a reduction in egg output at 1.25% (Fig. 3j,k). In flies in which Kir2.1 expression was restricted to the subset of brain neurons labeled by ATB-1, subterraneous egg deposition was largely unaffected (ATB-1>Otd-nls:FLP; UAS(FRT.mCherry)Kir2.1-GFP; Extended Data Fig. 6)^42^. Thus, ATB neurons may coordinate penetration of the substrate by the ovipositor and positively gate egg expulsion after successful penetration.

ATB silencing yields a complex array of phenotypes that strongly implicate ATB neurons in providing tactile feedback during egg laying that modulates the progression from bending to burrowing to egg expulsion. The mechanisms by which ATB neurons exert this control may rely on the spatial and morphological heterogeneity of terminalia bristles^32^. Individual sets of bristles may exhibit unique tuning properties and may function independently to modulate different phases of the behavioral progression^29,33,43,44^.

### Burrowing behavior adjusts to changes in substrate firmness

**Fig. 4:**
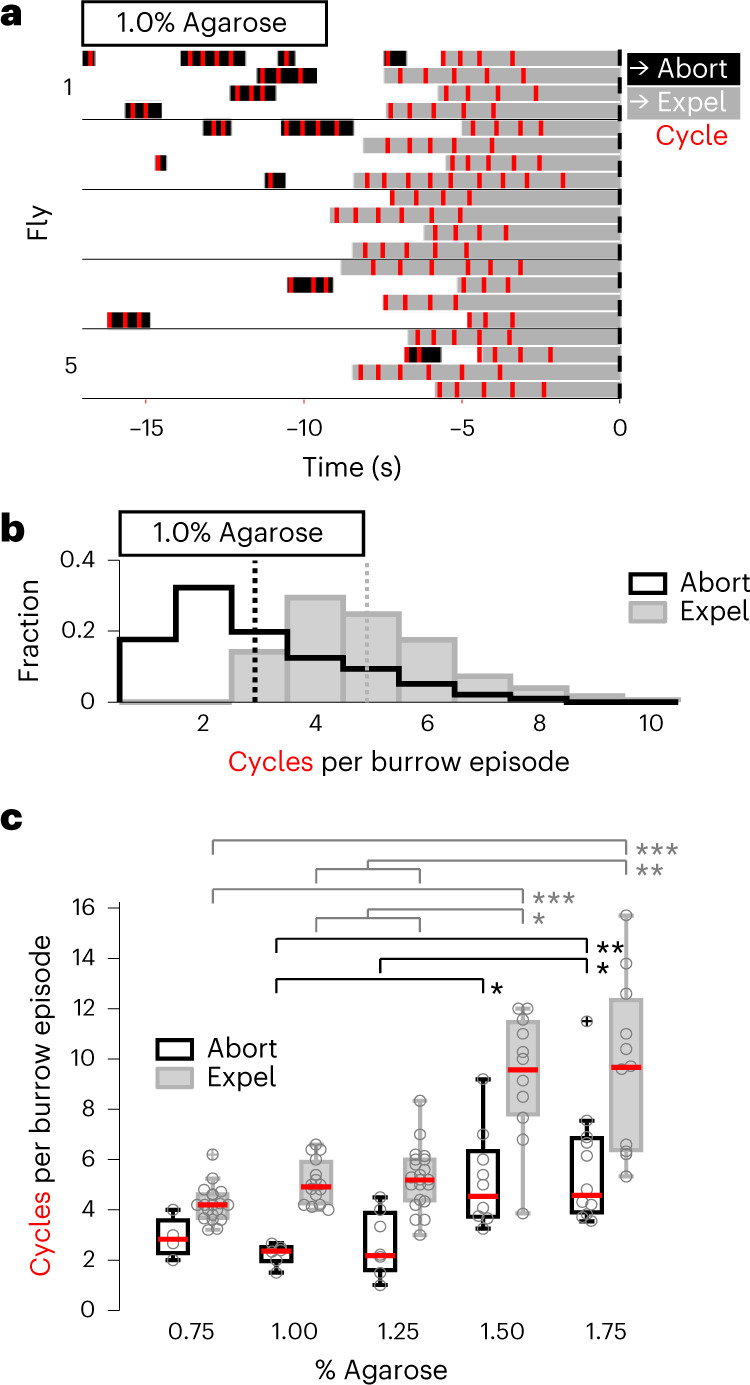
The substructure of burrowing adjusts to changes in substrate firmness. **a**, Representative ethograms depicting burrowing episodes in five flies (*n* = 4 events per fly); black, aborted episodes; gray, egg expulsion episodes; red, cycles within a burrowing episode; *t* = 0, egg out. Data are the same events depicted in Fig. 1c. **b**, Distributions of the number of cycles per burrowing episode; black, aborted episode; gray, egg expulsion episode. Dashed vertical lines indicate the mean value for each distribution. Data were pooled across all flies from experiments described in Fig. 1. **c**, Average number of cycles per burrowing episode for both aborted and egg expulsion episodes across substrates of increasing firmness. Only flies that exhibited two or more episodes for a given episode type were considered (abort episodes, *n* = 4, 6, 7, 9 and 11 flies per group; expel episodes, 19, 15, 21, 11 and 11 flies per group). Data are the same as those used in Fig. 2c–e. Box bounds indicate the 25th and 75th percentiles, red lines indicate the medians, and whiskers indicate the 5th and 95th percentiles; o, data from individual flies; +, outliers; *P < 0.05; **P < 0.01; ***P < 0.001. Data were analyzed by Kruskal–Wallis test with a post hoc Tukey’s HSD test (Supplementary Table 7).

The pivotal role of subterraneous egg placement in the progression of component behaviors led us to closely examine the substructure of burrowing (Fig. 4a). A burrowing episode is comprised of discrete cycles that begins with rhythmic ovipositor digging. As the surface is scored, the ovipositor extends into the substrate, and the egg emerges out of the uterus and into the ovipositor. Rhythmic pushing expels the egg out of the ovipositor and into the substrate, just beneath the surface. Completed egg expulsion halts the rhythm, terminating the burrowing episode, and the fly then detaches the ovipositor from the egg.

In chambers containing 1% agarose, egg expulsion required a minimum of three cycles and could require as many as ten cycles within a burrowing episode (Fig. 4b). The number of cycles was significantly lower in aborted episodes in which flies did not persist to egg expulsion (mean of three cycles for aborted episodes and five cycles for expulsion; *P* < 0.001, Wilcoxon rank-sum test), and burrowing could be aborted after any cycle within an episode (minimum of one and maximum of eight; Fig. 4b). This suggests that the decision to persist in burrowing may be determined after each individual cycle. Burrowing can therefore be extended or aborted and then reinitiated to achieve successful egg deposition.

We observed that the substructure of burrowing behavior was dramatically altered as the firmness of the agarose substrate was increased. The total number of burrow cycles required for egg expulsion increased over twofold (Fig. 4c) as the agarose concentration increased from 0.75% to 1.75%. Thus, additional burrowing cycles are required to dig and push the egg into the firmer substrates. Burrow episodes that were aborted also displayed a twofold increase in cycle count on firmer substrates (Fig. 4c). If the egg cannot be successfully deposited after an extended attempt, burrowing is aborted in search of a more favorable location. These data suggest that the transition to egg expulsion (‘egg out’) and the reset phase is contingent on the decision to persist in burrowing until the egg is completely expelled. The decision to persist in burrowing for additional cycles is likely to be informed by ongoing sensory feedback regarding the position of the egg as it is pushed through the uterus and ovipositor into the substrate.

### Internal sensory neurons activated by the progression of the egg

**Fig. 5:**
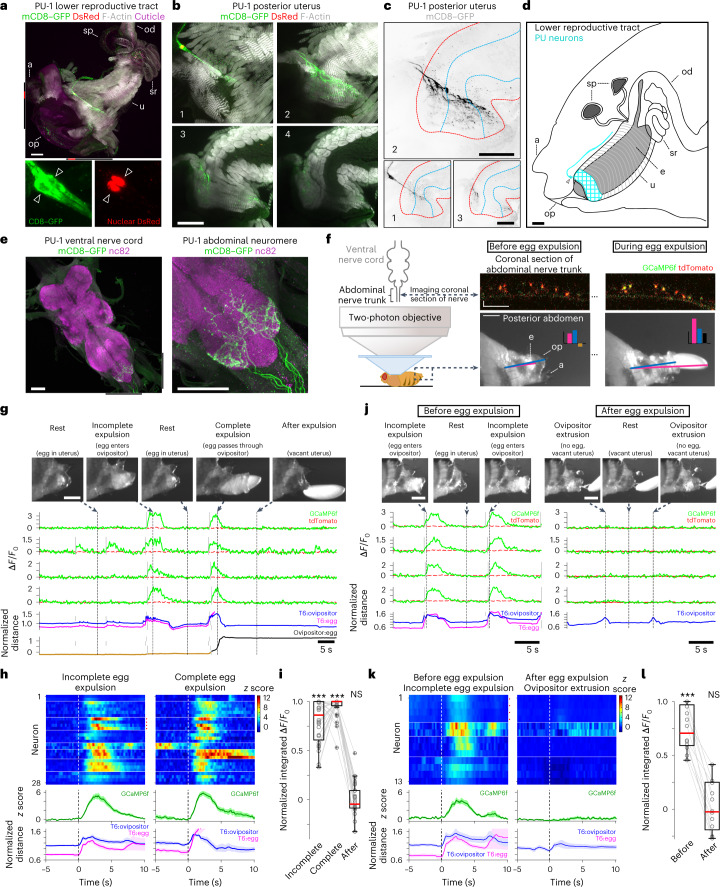
PU sensory neurons are activated by the progression of the egg. **a**, Top: representative image of the lower reproductive tract from four PU-1>RedStinger; mCD8–GFP females (lateral aspect) stained with anti-GFP (membrane of PU neurons, green), anti-DsRed (nuclei of PU neurons, red) and phalloidin (muscle F-actin, gray); autofluorescence, abdominal cuticle (magenta). Bottom (left and right): higher-resolution region of the top image (indicated by red bars flanking the top image) displaying two PU cell bodies (white triangles). The black bars flanking the top image indicate the region show in **b**. Here and in **d** and **f**, a indicates analia, op indicates ovipositor (hypogynium), sp indicates spermathecae, u indicates uterus (genital chamber), od indicates oviduct, sr indicates seminal receptacle, and e indicates egg. Scale bar, 50 μm (**b**–**e**). **b**, Higher-resolution region of the top image in **a** displaying PU labeling at four successive depths surrounding the posterior uterus (region 1 is the most superficial). **c**, PU neuron expression (anti-GFP) in the posterior uterus (depth indicated as in **b**). The red and blue dashed lines demarcate the outer and inner bounds, respectively, of the CMU. **d**, Diagram of the female posterior abdomen (lateral aspect) revealing the lower reproductive tract. PU neurons are labeled cyan (the triangle indicates the cell bodies). **e**, Representative image of the ventral nerve cord (left) and abdominal neuromere (right) from 15 PU-1>mCD8–GFP females stained with anti-GFP (PU neurons, green) and nc82 (synaptic neuropil, magenta). Black bars flanking the left image indicate the region shown at higher resolution on the right. **f**, Two-photon experimental setup involving simultaneous measurement of GcaMP6f (green) and tdTomato (red) fluorescence in axons within a coronal section of the abdominal nerve trunk (top right; scale bar, 10 μm) and videography of the posterior abdomen (bottom right; scale bar, 200 μm). Bottom right: magenta and blue lines connect the dorsal–posterior edge of T6 with the egg and ovipositor, respectively; the bar graph displays the normalized distances between T6:egg (magenta), T6:ovipositor (blue) and ovipositor:egg (brown, negative distance; black, positive distance; Methods). **g**, Representative experiment showing video snapshots of the posterior abdomen (top; scale bar, 200 μm), two-photon imaging of four PU neurons depicting relative fluorescence changes of GCaMP6f and TdTomato (middle; green and dashed red traces, respectively) and movement of the egg and ovipositor (bottom; Methods). Arrows and vertical dashed lines indicate the corresponding time point for each video snapshot. Vertical gray lines indicate the onset of calcium response events. Data are the same as those presented in Supplementary Video 4. **h**, Normalized PU responses and behavioral measures surrounding incomplete egg expulsion events (left) and completed egg expulsion (right). Top: individual neuron responses; horizontal white lines demarcate recordings performed in different flies. Cells from **g** are indicated by red dots. Middle and bottom: aggregate response of all neurons and aggregate behavioral measurements, respectively (darker traces, mean response; lighter area, s.e.m.); *n* = 28 neurons from eight flies; *t* = 0, behavioral event onset (Methods). **i**, Normalized population data showing the 3-s integrated Δ*F*/*F*_0_ fluorescence levels during incomplete egg expulsion and complete egg expulsion and after egg expulsion (*n* = 28 neurons). Here and in **l**, box bounds indicate the 25th and 75th percentiles, the red lines indicate the medians, and the whiskers indicate the 5th and 95th percentiles; o, data from individual neurons; +, outliers; ***P < 0.001; NS, *P* > 0.05. Data were analyzed by two-sided Wilcoxon signed-rank test compared to preexpulsion baseline (Supplementary Table 7). **j**, Representative experiment comparing PU neuron activity surrounding incomplete egg expulsion (left) and ovipositor extrusion events lacking an egg (right). The figure was constructed as in **g**. **k**, Normalized PU responses and behavioral measures surrounding incomplete expulsion events (left) and ovipositor extrusion events after egg expulsion (right). The figure was constructed as in **h**. Top: cells from **j** are indicated by red dots; *n* = 13 neurons from four flies. The same data are presented in Supplementary Video 5. **l**, Normalized population data showing the 3-s integrated Δ*F*/*F*_0_ fluorescence levels during incomplete expulsion events (‘before’) and ovipositor extrusion events (‘after’); *n* = 13 neurons.

**Extended Data Fig. 7:**
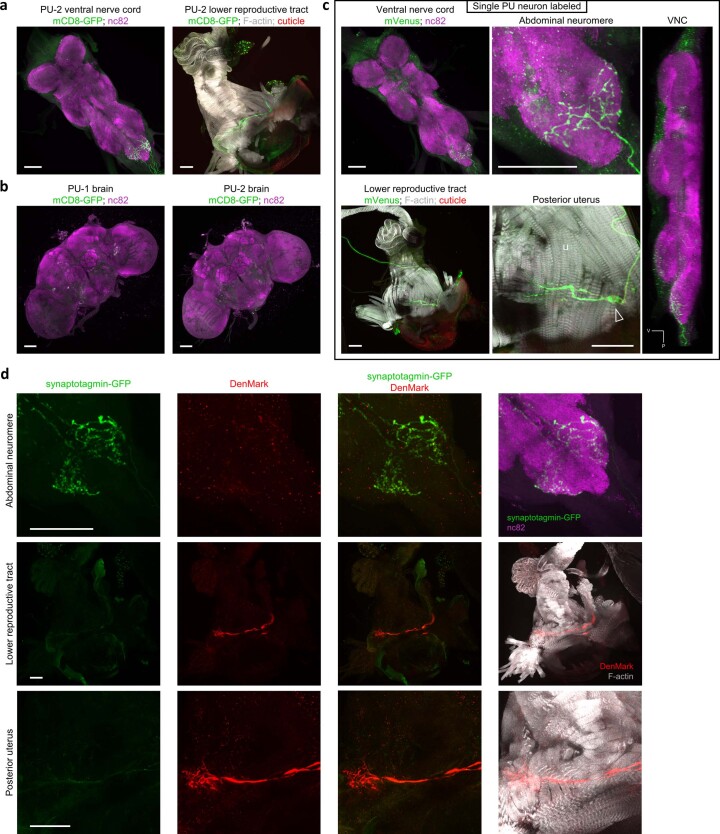
Expression pattern of PU-splitGAL4 lines. **a**, Representative images from PU-2 > mCD8-GFP females. Left: ventral nerve cord (one of 13 flies) stained with anti-GFP (membrane of PU neurons, green) and nc82 (synaptic neuropil, magenta). Right: lower reproductive tract (one of eight flies) stained with anti-GFP (green) and phalloidin (muscle f-actin, gray); autofluorescence abdominal cuticle (red). In addition to consistent labeling of the PU neurons (PU-1, 2.1 ± 0.4 ventral afferents per side, n = 17 sides in thirteen flies; PU-2, 2.0 ± 0.6; n = 11 sides in eight flies; mean ± s.d.), both lines exhibited inconsistent labeling in a small number of peripheral neurons that project to the dorsal abdominal neuromere (PU-1, 1.2 ± 0.6 dorsal afferents per side; PU-2, 0.5 ± 0.6; mean ± s.d.). Here and in **b**-**d**, scale bar, 50 μm. **b**, Representative images of the brain from PU-1>mCD8-GFP (left, one of three flies) and PU-2>mCD8-GFP (right, one of two flies) females, stained with anti-GFP (green) and nc82 (magenta). **c**, Representative images from hs-FLP; PU-2>(FRT.stop)CsChrimson-mVenus females (one of two flies) where stochastic labeling resulted in only a single PU neuron being labelled (Gordon and Scott, *Neuron* 61, 373–384 (2009)). Top two images and right image: ventral nerve cord stained with anti-GFP (mVenus-expressing PU neurons, green) and nc82 (magenta). Bottom two images: lower reproductive tract stained with anti-GFP (green) and phalloidin (muscle f-actin, gray); autofluorescence, abdominal cuticle (red); white triangle, PU cell body; u, uterus. Right: lateral projection of the ventral nerve cord after registration with a template; V, ventral; P, posterior. **d**, Representative images of the abdominal neuromere (top row, one of three flies) and lower reproductive tract (middle, bottom rows, one of three flies) from PU-2>DenMark, synaptotagmin-GFP females, stained with anti-DsRed (dendrites, red) and anti-GFP (synaptic vesicles, green)^52,53^.

We next screened a library of transgenic lines^45^ to identify candidate sensory neurons that innervate the lower reproductive tract and detect the passage of the egg through the uterus during burrowing^4,46–48^. We identified a cluster of sensory neurons whose cell bodies flank the posterior uterus and whose processes arborize along the outer surface of the distal-most fibers of the muscle that encircles the uterus (posterior uterine (PU) sensory neurons; Fig. 5a–d)^49^. We used the split-GAL4 intersectional strategy to generate two lines that labeled a pair of PU neurons on each side of the uterus (PU-1, 1.9 ± 0.5 cells per side, *n* = 17 sides in 13 flies; PU-2, 2.1 ± 0.3 cells, *n* = 11 sides in 8 flies; mean ± s.d.; Fig. 5a–c and Extended Data Fig. 7a,b). These neurons send projections centrally that terminate in the ventral-most neuropil of the abdominal neuromere, a sensory domain associated with multidendritic sensory neuron inputs^50,51^ (Fig. 5e and Extended Data Fig. 7a,c). We confirmed the polarity of PU neurons by targeted expression of both synaptotagmin–GFP^52^, a presynaptic marker, and DenMark^53^, a somatodendritic marker. Synaptotagmin–GFP was restricted to the central projections in the abdominal neuromere, while DenMark localized to the peripheral processes encircling the posterior uterus (Extended Data Fig. 7d). This pattern of dendritic innervation suggests that PU neurons may sense the passage of an egg through the posterior uterus into the ovipositor.

We therefore monitored the activity of PU neurons as the egg is expelled from the uterus. GCaMP6f was expressed in PU neurons, and calcium activity was recorded in flies mounted ventral-side up (Fig. 5f and Methods)^54,55^. Snapshots from a typical video recording over a 90-s window encompassing egg expulsion are shown in Fig. 5g, along with the corresponding activity of the four PU axons and behavioral measurements of the movement of the egg and ovipositor (Supplementary Video 4). Initially, we observed an incomplete expulsion event (Fig. 5g), where the egg advanced from the uterus (first frame) into the extruded ovipositor (second frame), after which the egg retreated into the uterus and the ovipositor retracted (third frame). During this event, calcium activity in PU neurons increased from baseline after the egg entered the ovipositor and returned to baseline when the egg retreated into the uterus. A second, complete expulsion event then occurred (fourth frame), and the PU neurons again responded after the egg entered the ovipositor, and the activity returned to baseline after the egg was completely expelled (fifth frame). These response properties were observed in all 28 PU neurons recorded from eight flies (Fig. 5h,i and Supplementary Fig. 4). PU neurons were not activated when the egg was at rest in the uterus. Moreover, PU neuron response was specific to the advancement of the egg into the ovipositor and not simply to the extrusion of the ovipositor. PU neurons did not respond to ovipositor extrusion events in flies lacking an egg (Fig. 5j–l and Supplementary Video 5). These observations demonstrate that PU neurons respond shortly after the egg passes through the posterior uterus into the ovipositor and may therefore inform circuits in the abdominal neuromere about the position of the egg during burrowing^18,40^.

### Silencing PU neurons disrupts the egg-laying sequence

**Fig. 6:**
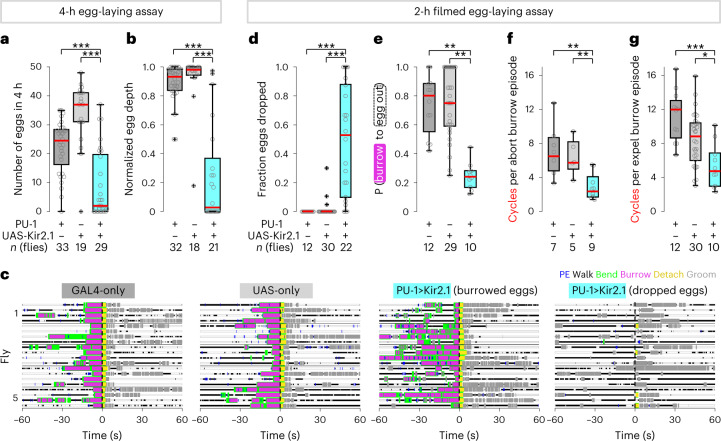
Silencing PU neurons reduces egg output and disrupts the egg-laying sequence. **a**, Number of eggs released on a 1% agarose substrate in 4 h (*n* = 33, 19 and 29 flies per group). Here and in **b** and **d**–**g**, box bounds indicate the 25th and 75th percentiles, the red lines indicate the medians, and the whiskers indicate the 5th and 95th percentiles; o, data from individual flies; +, outliers; *P < 0.05; **P < 0.01; ***P < 0.001. Data were analyzed by two-sided Wilcoxon rank-sum test followed by a Bonferroni correction (Supplementary Table 7). **b**, Average normalized depth of penetration of released eggs (*n* = 32, 18 and 21 flies per group). **c**, Representative ethograms of egg-laying behavior for genetic control flies (first and second graphs) and for PU-silenced flies (third (burrowed eggs) and fourth (spontaneously dropped eggs) graphs). Each ethogram depicts data from five flies (*n* = 4 events per fly); *t* = 0, egg out. **d**, Fraction of eggs spontaneously dropped without burrowing (*n* = 12, 30 and 22 flies per group). Only flies that released four or more eggs are considered here and in **e**–**g**. **e**, Average probability (P) of progression from burrowing to egg out (*n* = 12, 29 and 10 flies per group). Only flies that exhibited three or more burrowed eggs are considered. **f**, Average number of cycles per aborted burrowing episode (*n* = 7, 5 and 9 flies per group). Only flies that exhibited three or more aborted burrowing episodes are considered. **g**, Average number of cycles per egg expulsion burrowing episode (*n* = 12, 30 and 10 flies per group). Only flies that exhibited three or more egg expulsion burrowing episodes are considered.

We silenced PU sensory neurons to examine their role in egg-laying behavior by the targeted expression of Kir2.1 (PU-1>Kir2.1). In PU-silenced flies, we observed a dramatic reduction in egg output (mean of 9 eggs in 4 h compared to 23 and 34 in the two genetic controls; Fig. 6a and Supplementary Fig. 5a). Moreover, the majority of the eggs in PU-silenced flies were not deposited subterraneously on a 1.0% agarose substrate (29% deposited subterraneously versus 95% and 93% in both genetic controls; Fig. 6b and Supplementary Fig. 5b). This reduction in egg count was not a consequence of a mating defect. PU-silenced virgins showed no deficit in mating but exhibited a reduction in egg count after a single mating event (Supplementary Fig. 5c,d).

PU-silenced flies expelled 51% of the eggs in the typical fashion following burrowing but exhibited a 67% reduction in the progression from burrowing to egg expulsion (PU-1>Kir2.1, probability of 0.25 versus 0.75 for both genetic controls; Fig. 6c,e). Burrowing episodes were comprised of fewer cycles than burrowing episodes in control flies (Fig. 6f,g). Moreover, burrowing episodes culminating in egg expulsion resulted in the premature release of the egg on the substrate surface. These data suggest that PU neurons sense the passage of the egg into the ovipositor during a burrowing episode, promoting persistent burrowing to achieve subterraneous egg deposition.

The remaining 49% of eggs in PU-silenced flies were spontaneously dropped without burrowing or expression of any of the other behavioral components that typically precede egg expulsion (Fig. 6c). These eggs spontaneously emerged while the ovipositor was in midair and were removed by hindleg grooming. This phenotype was exhibited by 19 of 22 PU-silenced flies but was rarely observed in control flies (0% in 12 GAL4-only control flies and 2% in 3 of 30 UAS-only control flies; Fig. 6d). This distinct phenotype may implicate PU feedback in the regulation of musculature required for egg retention. Thus, the silencing of PU neurons resulted in deficits in burrowing and diminished and aberrant egg output.

### PU neurons control timing and direction of burrow transitions

**Fig. 7:**
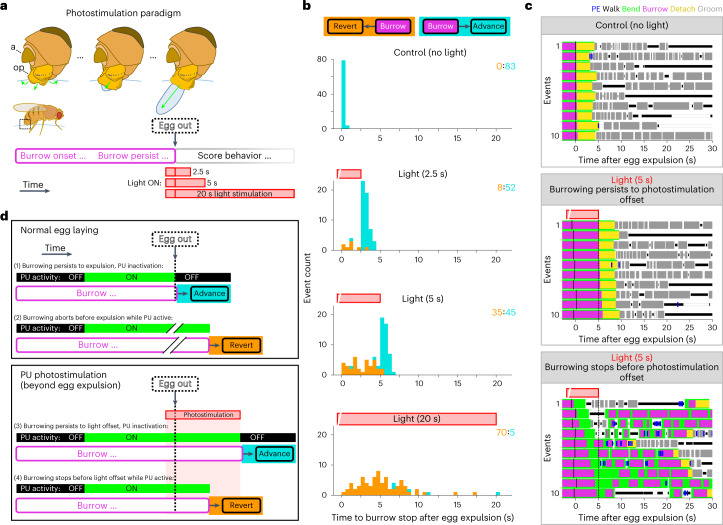
PU neurons control timing and direction of burrow transitions. **a**, Schematic of the photostimulation paradigm. The box drawn on the fly’s abdomen (middle left) depicts the region shown in higher detail above; a, analia; op, ovipositor. Photostimulation (655-nm light at 8 µW mm^–2^) was initiated during burrowing immediately before completed egg expulsion (egg out) and was sustained for variable amounts of time after egg expulsion. **b**, Stacked distributions of the timing that burrowing stopped after egg expulsion for control (top) and stimulus conditions. Events are color coded according to which transition was made after burrowing stopped; orange, flies reverted in the sequence; cyan, flies advanced to the reset phase (Methods and Extended Data Fig. 8d). Here and in **c**, the red bar above each plot indicates the photostimulation period. Data represent 298 events from 16 flies. The total number of events in each group is indicated in the top right. **c**, Representative ethograms of egg-laying behavior for no-light control events (top) and 5-s photostimulation events, separately depicting events where burrowing persisted throughout photostimulation (middle) and events where burrowing stopped during photostimulation (bottom); vertical black dashed line, photostimulation offset; black tick marks near *t* = 0, timing of completed egg expulsion for each event. **d**, Model for how PU neuron activity determines the timing and direction of burrow transitions. Top: normal egg-laying behavior. PU neurons at baseline (black, inactive) at the onset of burrowing become activated (green) after the passage of the egg into the ovipositor during burrowing and return to baseline after completed egg expulsion. Bottom: behavior during photostimulation. Vertical black dashed lines, time of completed egg expulsion (egg out); advance, fly progresses to the reset phase; revert, fly transitions to preceding components of the sequence.

**Extended Data Fig. 8:**
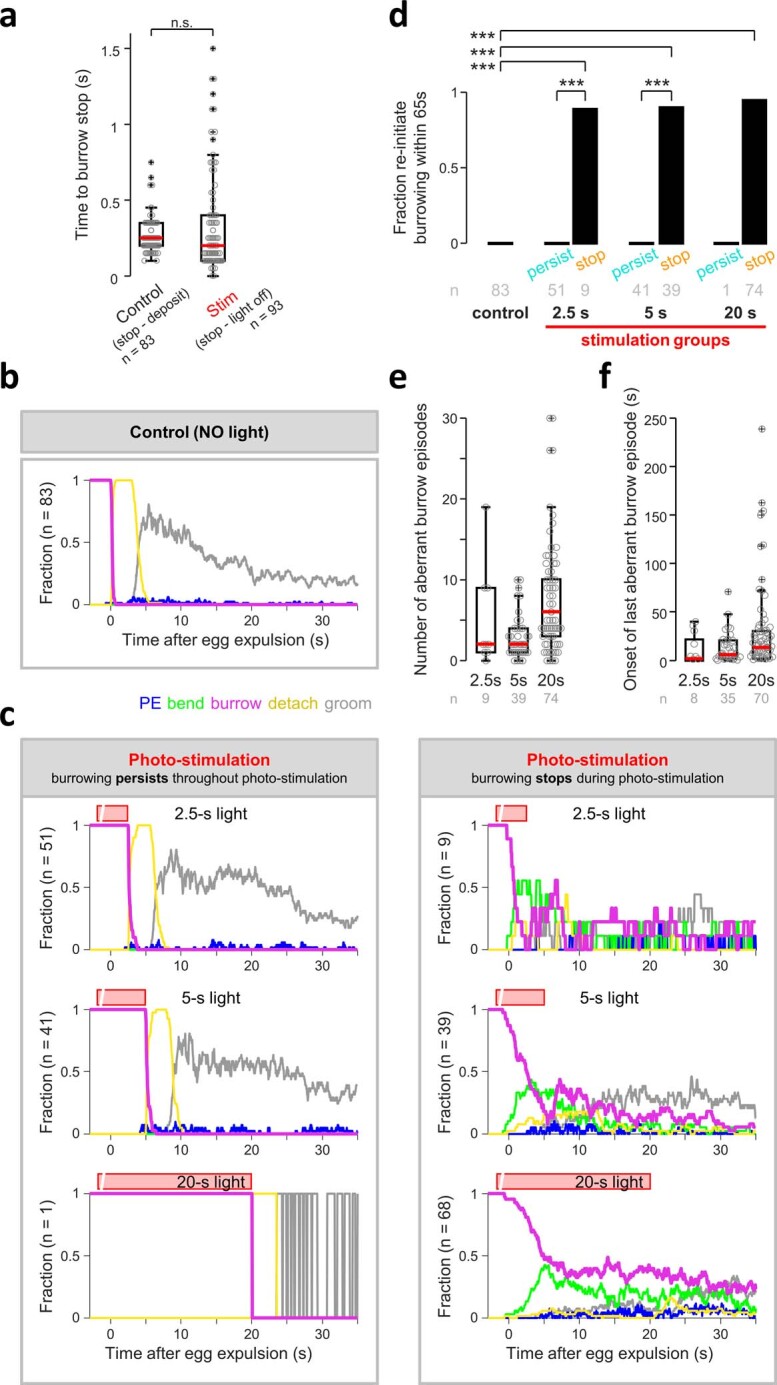
Phenotypes induced by PU photo-stimulation beyond egg expulsion. **a**, Timing that burrowing stopped after completed egg expulsion in no-light control events (control, n = 83 events) and after light offset in stimulation events where burrowing persisted throughout photo-stimulation (stim, n = 93). Here and in **e** and **f**, box bounds, 25th and 75th percentile; red line, median; whiskers, 5th and 95th percentile; o, data from individual events; +, outliers. n.s., p > 0.05, two-sided Wilcoxon rank sum test (Supplementary Table 7). **b**, Time course of annotated behaviors for no light control events. **c**, Time courses of annotated behaviors for three stimulus conditions. Left: burrowing persisted throughout photo-stimulation. Right: burrowing stopped during photo-stimulation. Red bar above each plot, period of photo-stimulation. **d**, Fraction of events where burrowing was re-initiated within 65 s of egg expulsion in control and three stimulus conditions. For stimulation groups, fraction plotted separately for events where burrowing persisted throughout photo-stimulation (persist), and events where burrowing stopped during photo-stimulation (stop). Events defined as reverting in the sequence if burrowing was re-initiated within 65 s of egg expulsion. ***p < 0.001, two-sided Fisher’s exact test (Supplementary Table 7). **e**, Number of aberrant burrowing episodes after egg expulsion for events where burrowing stopped during photo-stimulation for all three stimulus conditions. Episode defined as aberrant if occurring within 65 s of egg expulsion or a previous aberrant episode. **f**, Onset timing of last aberrant burrowing episode expressed after egg expulsion for events where burrowing stopped during photo-stimulation and the fly reverted in the sequence, plotted for all three stimulus conditions. Last aberrant episode after the fly reverted in the sequence defined as the first episode preceding a 65-s window devoid of burrowing behavior. Light offset during an aberrant burrow episode significantly increased the probability of progressing to reset: 14 of 33 aberrant burrow episodes that spanned light offset progressed to reset compared to 0 of 106 episodes that stopped before light offset and 93 of 457 episodes that started after light offset (p = 1.40×10^−10^, p = 0.0048 comparing group 1 to group 2 or group 3, respectively, one-sided Fisher’s exact test).

We next explored the function of PU neurons by targeted expression of the red-light-activated channelrhodopsin CsChrimson (PU-1>CsChrimson)^56^. We devised a physiological paradigm in which we photostimulated PU neurons in the context of egg laying. We have shown that PU neurons are activated after passage of the egg through the uterus into the ovipositor and that their activity returns to baseline after completed expulsion. We reasoned that prolonged PU neuron activation beyond egg expulsion may mimic the continued presence of the egg within the posterior uterus and ovipositor and delay progression along the behavioral sequence. In normal egg-laying behavior, burrowing ceases after egg expulsion, and the fly detaches from the egg, grooms its ovipositor and transitions to the reset phase. We photostimulated PU neurons during burrowing with a pulse of light that was triggered immediately before the completion of egg expulsion and remained on after egg expulsion for different durations (2.5, 5 or 20 s; light onset 0.9 ± 1.1 s before complete egg expulsion; *n* = 215 photostimulation events; mean ± s.d.; Fig. 7a). Prolonged PU neuron activation beyond egg expulsion resulted in the aberrant persistence of burrowing without transitioning to detachment despite the absence of an egg in the uterus (Fig. 7b). With 20-s photostimulation, flies stopped burrowing an average of 5.5 ± 1.3 s beyond egg expulsion (*n* = 15 flies; Fig. 7b). Flies that burrowed throughout the photostimulation period ceased burrowing after light offset (Fig. 7b and Extended Data Fig. 8a). As expected, given our observations with 20-s photostimulation, burrowing persisted until light offset frequently for 2.5-s stimulations, in approximately half of 5-s stimulations and almost never for 20-s stimulations (52 of 60 stimulations, 45 of 80 and 5 of 75, respectively). In the remaining events, burrowing persisted for variable durations but stopped before light offset. These data suggest that PU activation promotes burrowing persistence. However, persistent burrowing is not sustained, suggesting an intrinsic temporal control on the duration of burrowing.

Flies that persist in burrowing throughout photostimulation abruptly stopped burrowing after light offset and transitioned to the behavioral sequence normally triggered by egg expulsion (Fig. 7c). This behavior was observed for all three stimulus durations (Fig. 7b, Extended Data Fig. 8b,c and Supplementary Video 6). These data support the argument that PU neurons are activated by the passage of the egg into the ovipositor and drive the persistence of burrowing. These observations also suggest that a decrease in PU activity signals the completion of egg expulsion, resulting in the transition to detachment, grooming and the reset phase (Fig. 7d).

In the photostimulation events where burrowing stopped before light offset, flies exhibited exploration and deposition behaviors in new locations despite the fact that they had already expelled an egg (Fig. 7b,c, Extended Data Fig. 8b–d and Supplementary Video 7). This behavior appears to recapitulate the behavior observed after aborting a burrowing episode in normal egg-laying behavior. In wild-type flies, prolonged burrowing and PU activation without expulsion may signal the inability to deposit an egg, resulting in abortion of the episode (Fig. 7d). In the photostimulation experiment, the flies may be unaware of having laid an egg, and the prolonged activation of PU neurons may also signal the inability to deposit an egg, resulting in abortion (Fig. 7d). These flies persistently expressed exploration and deposition behaviors and exhibited numerous burrowing episodes for up to several minutes beyond light offset despite the absence of an egg in the uterus (Extended Data Fig. 8e,f). After the decision to abort and revert, a decrease in PU activity (photostimulation offset) no longer triggers the transition to reset.

**Extended Data Fig. 9:**
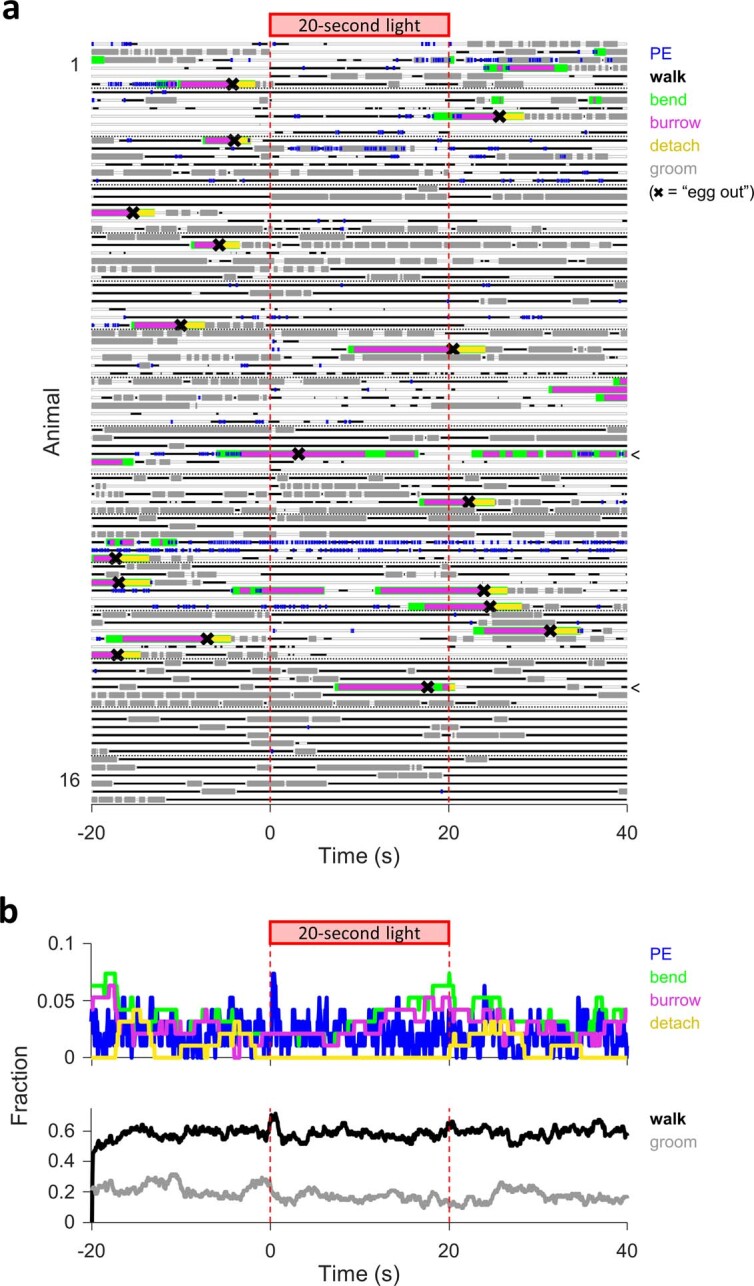
PU photo-stimulation outside the context of burrowing. **a**, Representative ethograms of behavior surrounding a 20-s photo-stimulation pulse delivered at 90-s intervals, independent of the ongoing behavioral state of the fly (Methods). Ethogram depicts data from 16 flies (n = 6 events per fly). Horizontal black dotted lines demarcate data from different flies. The component actions are color coded as in Fig. 1c, and completed egg expulsion events (egg out) are indicated by a black ‘X’. Photo-stimulation did not induce an overt behavioral response with the exception of two out of the 96 events where egg expulsion was completed within the stimulation window and the animal reverted in the sequence (‘<’ symbols at the right indicates these two events). Here and in **b**, *t* = 0, photo-stimulation onset; red bar above plot and vertical dashed magenta lines, period of photo-stimulation. **b**, Time course of annotated behaviors depicted in **a**.

These experiments demonstrate a role for PU activation and inactivation in the context of a burrowing episode. We therefore asked whether photostimulation of PU neurons could impact behavior outside the context of burrowing. We photostimulated PU neurons for 20 s at 90-s intervals, independent of the ongoing behavioral state of the fly. Neither the photostimulation period nor its offset induced an overt behavioral response (Extended Data Fig. 9). Thus, optogenetic activation only results in persistent burrowing during an ongoing burrowing episode. Moreover, a decrease in PU neuron activity only signals the completion of egg expulsion and the transition to postdeposition behaviors in the context of an ongoing burrowing episode.

### A pair of uterine motor neurons expels the egg during burrowing

**Fig. 8:**
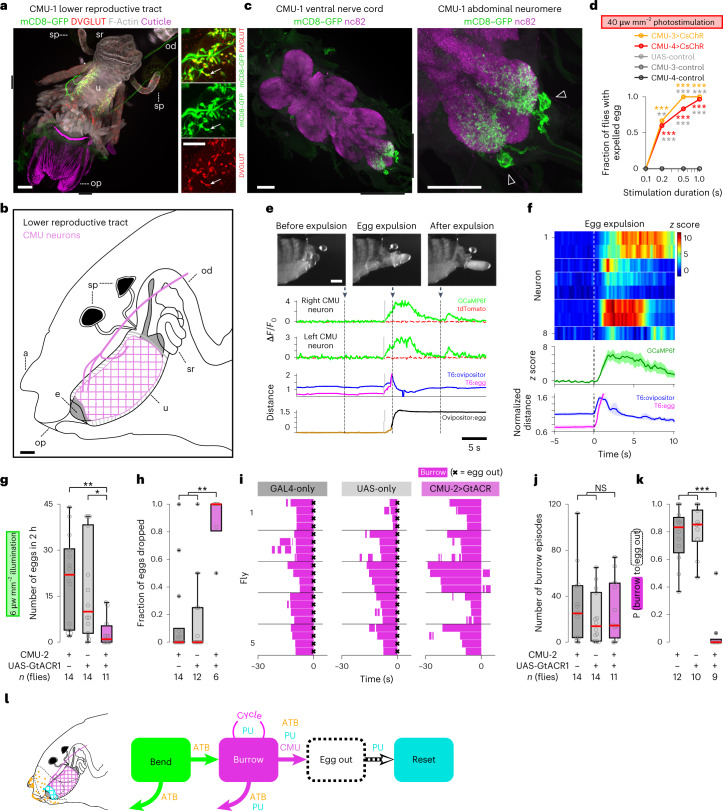
A pair of uterine motor neurons expels the egg during burrowing. **a**, Left: representative image of the lower reproductive tract (ventral aspect) from four CMU-1>mCD8–GFP females, stained with anti-GFP (membrane of CMU neurons, green), phalloidin (muscle F-actin, gray) and anti-DVGLUT (glutamatergic synapses, red); autofluorescence, ovipositor cuticle (magenta). Right: high-resolution images of axon terminals; arrow, individual bouton. Here and in **c**, black bars flanking the left image indicate the region shown at higher resolution on the right. Here and in **b**, op indicates ovipositor, sp indicates spermathecae, u indicates the uterus, od indicates the oviduct, sr indicates the seminal receptacle, a indicates the analia, and e indicates the egg. Scale bar, 50 μm (**b**,**c**). **b**, Diagram of the female posterior abdomen (lateral aspect) revealing the lower reproductive tract. A CMU axon is labeled magenta. **c**, Image of the ventral nerve cord (left) and abdominal neuromere (right) corresponding to the CMU-1>mCD8–GFP female in **a** stained with anti-GFP (CMU neurons, green) and nc82 (synaptic neuropil, magenta); triangles, CMU cell bodies. **d**, Fraction of flies that expelled an egg after delivery of photostimulation pulses of varied duration (CMU-3>CsChR, *n* = 4, 15, 13 and 11 flies per group; CMU-4>CsChR, *n* = 30, 30, 30 and 30 flies per group; UAS-control, *n* = 16, 17, 16 and 17 flies per group; CMU-3-control, *n* = 4, 9, 8 and 9 flies per group; CMU-4-control, *n* = 10, 10, 10 and 10 flies per group). Colored and gray asterisks indicate significance for comparisons with GAL4-only control flies and UAS-only control flies, respectively; **P < 0.01; ***P < 0.001. Data were analyzed by two-sided Fisher’s exact test (Supplementary Table 7). **e**, Representative experiment showing video snapshots of the posterior abdomen (top; scale bar, 200 μm), two-photon imaging of two CMU axons depicting relative fluorescence changes of GCaMP6f and TdTomato (middle; green and dashed red traces, respectively) and movement of the egg and ovipositor (bottom; Methods). Arrows and vertical dashed lines correspond to the time point for each video snapshot. Vertical gray lines indicate the onset of calcium response events. **f**, Normalized PU responses and behavioral measures surrounding incomplete egg expulsion events (left) and completed egg expulsion (right). Top: individual neuron responses; horizontal white lines demarcate recordings performed in different flies; neuron 1 and 2 from **e**. Middle and bottom: aggregate response of all neurons and aggregate behavioral measurements, respectively. Darker traces indicate the mean response, and the lighter area represents s.e.m.; *n* = 8 neurons from five flies; *t* = 0, behavioral event onset (Methods). **g**, Number of eggs released on a 1% agarose substrate in 2 h (*n* = 14, 14 and 11 flies per group). Here and in **h**, **j** and **k**, box bounds indicate the 25th and 75th percentiles, the red lines indicate the medians, and whiskers indicate the 5th and 95th percentiles; o, data from individual flies; +, outliers; **P < 0.01; ***P < 0.001; NS, *P* > 0.05. Data were analyzed by two-sided Wilcoxon rank-sum test followed by a Bonferroni correction (Supplementary Table 7). **h**, Fraction of eggs spontaneously dropped without burrowing (*n* = 14, 12 and 6 flies per group). Only flies that released two or more eggs were considered. **i**, Representative ethograms depicting burrowing episodes for genetic control flies (left and middle) and for CMU-silenced flies (right). Each ethogram depicts data from five flies (*n* = 4 events per fly); left and middle, *t* = 0, egg out (indicated by ×); right, *t* = 0, time that burrowing stopped. **j**, Number of burrowing episodes in 2 h (*n* = 14, 14 and 11 flies per group). **k**, Average probability (P) of progression from burrowing to egg out (*n* = 12, 10 and 9 flies per group). Only flies that exhibited two or more burrowing episodes were considered. **l**, Summary of egg-laying sequence transitions influenced by identified sensory and motor neurons; cycle loop, repeating cycles within burrow episode.

**Extended Data Fig. 10:**
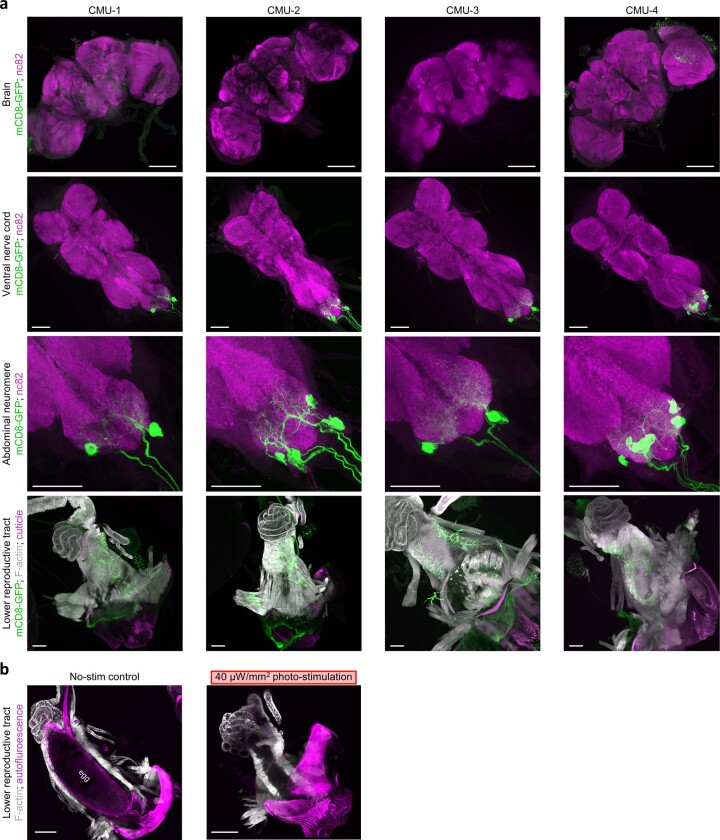
Expression pattern of CMU-splitGAL4 lines. **a**, Representative images of the brain (top row), ventral nerve cord (second, third rows), and lower reproductive tract (bottom row) from CMU-splitGAL4>mCD8-GFP females, stained with anti-GFP (membrane of CMU neurons, green). Top three rows: tissue also stained with nc82 (synaptic neuropil, magenta). Bottom row: tissue also stained with phalloidin (muscle f-actin, gray); autofluorescence, abdominal cuticle (magenta). Representative brain images (top row) from 12, 2, 2, 6 flies. Representative ventral nerve cord images (second, third rows) from 8, 22, 6, 12 flies. Representative lower reproductive tract images (bottom row) from 8, 11, 2, 10 flies. Images in third row are higher resolution regions of images in second row. For quantitation of expression patterns in the ventral nerve cord and lower reproductive tract, see Supplementary Table 4. Here and in **b**, scale bar, 50 μm. **b**, Representative images of the lower reproductive tract from gravid CMU-4>CsChrimson females that were flash frozen in liquid nitrogen without (left, one of two flies) or with (right, one of four flies) concurrent photo-stimulation (655 nm light at 40 µW/mm^2^). Prior to experiment, gravid females were maintained in the dark in an environment that ensured egg retention in the uterus, as in Fig. 8d (Methods).

The transition from burrowing to egg expulsion represents the final decision point in the egg-laying sequence and results in the transition to reset. We screened an image database^45^ to identify transgenic lines that target motor neurons innervating the uterus and involved in expelling the egg. We identified a symmetric pair of large neurons in the abdominal neuromere that project into the abdominal nerve trunk and ramify along the ipsilateral length of the muscle that encircles the uterus^49^. We used the split-GAL4 intersectional strategy to generate four lines with restricted expression in these neurons (circular muscle of the uterus (CMU) neurons; Fig. 8a–c, Extended Data Fig. 10a and Supplementary Table 4). Their axon terminals exhibit abundant boutons that stain with an antibody to *Drosophila* VGLUT, a marker for glutamatergic motor neuron synapses (Fig. 8a)^57,58^.

We asked whether stimulation of CMU neurons could trigger egg expulsion by expressing the channelrhodopsin CsChrimson in these neurons. Optogenetic activation reliably induced egg expulsion in gravid females (Fig. 8d). Moreover, following activation of CMU neurons, histological analysis revealed that the uterus was dramatically constricted (Extended Data Fig. 10b). Egg expulsion did not occur after photostimulation of control flies harboring only UAS-CsChrimson or the split-GAL4.

We used two-photon imaging to demonstrate that CMU neurons are indeed active when a fly expels an egg. GCaMP6f was expressed in CMU neurons, and calcium activity was recorded, as in Fig. 5f. We observed an acute increase in calcium activity concurrent with egg expulsion (Fig. 8e,f). These observations suggest that the CMU neurons are active during natural egg laying, expelling the egg during burrowing.

We also expressed the anion channelrhodopsin GtACR1 (ref. ^59^) to silence CMU neurons and examine their functional role in egg-laying behavior. In CMU-silenced flies, we observed a dramatic reduction in egg output compared to control flies (Fig. 8g and Supplementary Fig. 6). Moreover, CMU-silenced flies spontaneously dropped 89% of their eggs without burrowing (Fig. 8h). Egg-laying behavior was intact in these flies, and they engaged in a comparable number of burrowing episodes as control flies (Fig. 8i,j). However, burrowing almost never culminated in egg expulsion in flies with silenced CMU neurons (Fig. 8k). Thus, CMU neuron activity is necessary to progress from burrowing to egg expulsion, the final decision point in the egg-laying sequence (Fig. 8l).

## Discussion

We have characterized the structure of egg-laying behavior in the fly and demonstrate that it consists of a sequence of component actions analogous to Nikolaas Tinbergen’s ‘reaction chain’^3^. Tinbergen portrayed innate behaviors as a reaction chain, in which each component action of the sequence enhances the probability of encountering releasers, or ‘sign stimuli’, that promote progression to a subsequent component. This organization of component actions provides decision points at the junctures of component behaviors that ensure the successful progression toward the consummate act that satisfies the drive.

Our data demonstrate that the individual components of egg-laying behavior are not simply motor acts but also acts of sensory evaluation of the external and internal world that govern behavioral progression. During exploration, substrate cues are encountered while walking and proboscis sampling that may identify a suitable location for egg deposition^6,11,17^. The flies then initiate more refined local exploration involving abdominal bending to permit sampling with the abdominal terminalia. We have identified a class of external sensory neurons (ATB neurons) that innervate tactile hairs on the abdominal terminalia^29,31^, which contact the substrate and regulate the transition from bending to burrowing. During burrowing, the ovipositor is used to score the surface, extend into the substrate and expel the egg subterraneously. We further describe a pair of uterine motor neurons (CMU neurons) that enact this critical transition of burrowing to egg expulsion. The expulsion of the egg triggers egg detachment and the transition to the final behavioral phase, grooming and ovulation, facilitating the reinitiation of the sequence. We also identified a cluster of interoceptive sensory neurons (PU neurons), likely to be proprioceptive^4,46–48^, that signal the passage of the egg through the uterus into the ovipositor during burrowing and coordinate the transition to the final reset phase. Behavioral analysis along with genetic manipulation suggest that information from the PU neurons can either drive the persistence of burrowing, resulting in egg expulsion, or prompt the cessation of burrowing if the egg cannot be expelled after an extended attempt. Finally, diminished activity in PU neurons during burrowing signals the completion of egg expulsion and initiates the transition to the reset phase. These results provide a logic for a reaction chain in which sensory information at critical junctures guides flexible adjustments in component behaviors to achieve subterraneous egg deposition across varied environmental conditions.

The organization of innate behaviors into a sequence of component actions may confer additional adaptive advantages. Individual components in an innate behavioral sequence may be differentially responsive to the distinct sensory stimuli that promote transitions along the sequence. A given stimulus may behaviorally impact only one of the components in the sequence. Each component therefore establishes a context that filters relevant sensory input. For example, during the hunting of bees, digger wasps first visually identify a target bee. The odor of bees has no impact during this visual search, but once a bee has been spotted, the bee odor triggers an acute strike^3,60^. Similarly, we observe context-dependent behavioral responses to the activation of PU neurons. Photostimulation of PU neurons during a burrowing episode results in persistent burrowing, whereas activation at other times in the sequence does not elicit a behavioral response. Moreover, only during burrowing does a decline in PU neuron activity result in the transition to the reset phase. Thus, the behavioral impact of sensory stimuli differs for each of the components in a sequence. Each component therefore displays selective attention to distinct stimuli that structures the transition between behaviors to accommodate a complex and variable sensory environment.

In addition, each component in the sequence affords an entry point for adaptive evolutionary change. Changes in specific components of egg-laying behavior that accommodate a new ecological niche can occur without perturbing the overall sequence. For example, changes in substrate preferences during exploration may occur as an evolutionary adaptation to a changing environment^7,8,61^. Alterations in subsequent components, such as burrowing, may then be necessary to accommodate changes in the properties of the novel substrate. *D. melanogaster* and *D. suzukii* have different preferences for the site of egg laying^7^. *D. suzukii* females prefer to deposit eggs within firmer ripe fruit, whereas *D. melanogaster* favors softer rotting fruit. Egg-laying behavior is comprised of the same sequence of component actions in the two species, but *D. suzukii* females exhibit dramatically prolonged burrowing episodes^14^. Episodes in *D. suzukii* can persist for over 100 s, whereas *D. melanogaster* burrowing on the same substrate does not extend for more than 9 s. Interestingly, *D. suzukii* has also evolved an enlarged and serrated ovipositor^62^. These changes do not alter the sequence of behaviors but illustrate evolutionary adaptations that allow *D. suzukii* to deposit eggs within firmer fruits. A similar logic holds for male *Drosophila* courtship behavior, where adjustments in different steps in a conserved sequence (for example, foreleg pheromone sampling and singing) can be independently altered, and these modifications play critical roles in the sexual isolation of related species^63–65^.

An innate behavioral repertoire is thought to be initiated by higher-order brain centers that represent a specific motivational state or drive^3,66–71^. These centers are activated by stimuli relevant to the drive and then select an appropriate motor program for action^15,72–74^. A signal is then transmitted to preconfigured circuits in the ventral nerve cord or spinal cord that are capable of producing a coordinated sequence of motor actions^18,40,75–78^. Pivotal intermediaries in this pathway are the descending interneurons that link the output of higher brain centers with the appropriate local circuits in the ventral nerve cord^15,18,24,40,72,74,79–83^. One intermediary eliciting components of egg laying in *D. melanogaster* has been recently identified, the descending oviDN cluster of neurons^15^. OviDNs are necessary and causal for the expression of the terminal components of the egg-laying sequence: abdominal bending, ovipositor burrowing and egg expulsion. Higher-order brain centers disinhibit the oviDN cluster following mating and modulate oviDN activity in response to mechanical and gustatory stimuli presented to the legs^15,17^. Thus, oviDNs are poised to induce the transition from exploration (walking and proboscis sampling) to later steps in the sequence, resulting in egg deposition. Although activation of the oviDNs is capable of eliciting the complete sequence from abdominal bending to egg expulsion, our observations demonstrate that transitions along this late sequence are exquisitely sensitive to ongoing sensory feedback. We observe that abdominal bending does not always lead to burrowing and identify that this transition is modulated by tactile feedback from ATB neurons. Furthermore, the duration of burrowing and the transition to the reset phase are informed by feedback from PU neurons that sense the presence of the egg in the ovipositor. Thus, once the activation of oviDNs initiates the egg deposition motor program, sensory information acquired during the ensuing behavioral sequence governs the progression of the component actions to satisfy the drive.

## Methods

### Fly stocks and genotypes

All experiments were performed using 3- to 20-d-old females. For detailed fly stock sources and genotypes, see Supplementary Tables 5 and 6.

### Fly husbandry

Flies were reared at 25 °C and 55% relative humidity on a 12-h light/12-h dark cycle in vials containing cornmeal-agarose food. Females used in egg-laying experiments were genotyped under CO_2_ anesthesia within 1 d of eclosion and transferred to a vial containing an enriched medium (Nutri-Fly GF, Genesci Scientific) in a ratio of 4 females to 5 males, with a minimum of 12 and a maximum of 20 females per vial^48^. Egg-laying experiments were performed 5 to 7 d after eclosion. All experiments were initiated ±2 h of lights off. For optogenetic experiments, flies were reared and maintained in complete darkness, and all-*trans*-retinal (0.4 mM; Santa Cruz Biotechnology) was included in the enriched medium.

### Wild-type and loss-of-function behavior

For quantifying behavior at high resolution, single females were filmed in parallel within a custom three-dimensional-printed assembly containing six chambers (Shapeways). Individual chambers were 4.1 mm deep and tapered from top to bottom (7.3 mm × 5.8 mm to 6.7 mm × 4.3 mm). One side of the chamber was open to a reservoir, within which the agarose-based substrate (Affymetrix Agarose-LE, 32802) plus 3% acetic acid (vol/vol; Sigma-Aldrich, 338826) was poured and allowed to set for 30 min. Flies were introduced by gentle aspiration, the assembly was placed at the center of a 5-cm off-axis ring light (530 nm; Metaphase Technologies), and video recording was performed using a GigE camera (Basler Ace acA2000-50gmNIR) attached to a ×0.5 telecentric lens (Edmund Optics, 54-798) at 20 Hz (682 × 540 pixels per chamber) via pylon Viewer software (Basler). Experiments lasted 2 h.

The study of egg depth of penetration was performed in a custom acrylic assembly with 16 individual chambers (18.5 mm × 18.5 mm × 6 mm). Flies were introduced by gentle aspiration and allowed to habituate to the chamber. Forty milliliters of substrate containing agarose and 3% acetic acid was poured into a 120-mm square petri dish (Greiner) and allowed to set for 30 min. The experiment was initiated by removal of the thin plastic barrier separating the flies from the substrate, and the whole assembly was then placed in the dark for 4 h.

### Immunostaining and confocal microscopy

Flies were anesthetized with CO_2_ and fixed (2% paraformaldehyde in 75 mM lysine and 37 mM sodium phosphate buffer, pH 7.4) for 2 h at room temperature. The flies were then removed, and the brains, ventral nerve cord and lower reproductive tract were dissected in PBS containing 0.3% Triton X-100 (PBST), blocked with 10% normal goat serum diluted in PBST for 30 min and incubated in a primary antibody mix overnight at 4 °C. Subsequently, the tissue was washed for multiple rounds with PBST before being incubated in a secondary antibody mix overnight at 4 °C. A final round of PBST washing occurred before the tissue was mounted using VectaShield (Vector Laboratories) and imaged using an LSM 710 laser-scanning confocal microscope with a ×25/0.8 DIC or ×40/1.2 W objective (Zeiss). Primary antibodies used were mouse anti-bruchpilot (nc82; 1:10; Developmental Studies Hybridoma Bank), chicken anti-GFP (1:1,000; Aves Labs), rabbit anti-DsRed (1:500; Clontech), rabbit anti-NOMPC (1:5,000)^35^ and rabbit anti-DVGLUT (1:10,000)^57^. Secondary antibodies used were Alexa Fluor 633 goat anti-mouse, Alexa Fluor 488 goat anti-chicken, Alexa Fluor 488 goat anti-rabbit and Alexa Fluor 555 goat anti-rabbit (all at 1:200; Life Technologies). To visualize F-actin, Alexa Fluor 633 phalloidin was included in the secondary antibody mix (1:200; Life Technologies). Acquired images were processed using the Fiji distribution of ImageJ (NIH).

### Thoracic dissection for calcium imaging

Three- to 20-d-old females were anesthetized on ice, and the wings were removed before being mounted (ventral-side up) on a square acrylic platform using UV-curable glue (AA 3104, Loctite) and UV illumination (LED-200, Electro-Lite). The head and abdomen were lightly pressed down to ensure complete mounting from the head to just anterior of the analia. All legs were cut at the trochanter before, using a 30-gauge needle, a thin well was created with petroleum jelly that encompassed the remaining leg coxa and ranged from the neck connective to the anterior abdomen. A custom imaging platform with a hole (1 mm × 750 μm) at the bottom of a pyramidal basin was positioned using putty such that the hole was centered on the hindleg coxa. The basin was filled with external saline (108 mM NaCl, 5 mM KCl, 2 mM CaCl_2_, 4 mM MgCl_2_, 4 mM NaHCO_3_, 1 mM NaH_2_PO_4_, 5 mM trehalose, 10 mM sucrose and 5 mM HEPES, adjusted to pH 7.3) before the remaining coxa of the middle and rear legs were removed, along with the surrounding preepisternum, the internal sternal apophysis and any visible trachea, revealing a rectangular window above the abdominal neuromere and proximal extent of the abdominal nerve trunk. Finally, the basin was drained, and fresh saline was gently flushed over the window.

### Two-photon functional imaging

Pilot experiments revealed that gravid females mounted ventral-side up reliably expel an egg in midair within 30–60 min. Experiments were initiated immediately after completion of the dissection. The acrylic platform was secured adjacent to a camera and high-magnification lens setup (Point Grey USB3 camera, CM3-U3-13S2M-CS; InfiniProbe S-80 right angle video microscope lens) and infrared band-pass filter (Thorlabs, FGB25S) that, when illuminated by a nearby infrared (850-nm) LED lamp, allowed for high-resolution video recordings of the posterior abdomen concurrent with two-photon imaging.

Two-photon experiments were performed using an Ultra Microscope (Bruker) coupled to a Ti:Sapphire laser (Chameleon Vision, Coherent) via PrairiewView software (Bruker), with a GaAsp detector (Hamamatsu Photonics) for GCaMP6f and a photomultipier tube for tdTomato imaging. A ×40/0.80-NA water immersion objective (Nikon) was used, and the laser was tuned to 925 nm; the power measured after the objective ranged from 5 to 7 mW. The abdominal neuromere and abdominal nerve trunk were located using the microscope oculars and positioned near the center of the field of view by two-photon imaging. Using the tdTomato anatomical marker, a stretch of the abdominal nerve trunk where the axons were separated and ran in parallel was selected for the coronal section^55^. Coronal section imaging was performed at 10 Hz, covering 42.4 μm in *x* and 60 μm in *z* (512 × 85 pixels per image; 1.2-μs pixel dwell time). Small adjustments in the *x* and *z* dimensions were made as needed throughout the experiment to compensate for drift.

Axons corresponding to PU or CMU neurons were determined by two-photon imaging at the conclusion of the experiment. Axon projections were traced anteriorly, identifying PU or CMU axons by their expression pattern within the abdominal neuromere.

### Optogenetic perturbations during behavior

For the optogenetic stimulation of PU neurons during egg-laying behavior, the high-resolution filming apparatus described above was slightly modified. Chambers were illuminated with an infrared 5-cm off-axis ring light (880 nm; Metaphase Technologies), a single 655-nm high-power LED (Luxeon Star) was installed adjacent to the video lens to deliver red-light stimulation, and an infrared band-pass filter was mounted in front of the lens. A custom MATLAB graphical user interface (GUI) was used to select the stimulus condition and control the timing of the light stimulus via an Arduino UNO (Arduino) and LED controller (BuckPuck 700 mA, Luxeon Star). Experiments were performed on a 0.8% agarose substrate plus 3% acetic acid. As the egg neared complete expulsion during burrowing, a trigger was pressed that turned the light stimulus on. The instant the egg was fully expelled, a second trigger was pressed, initiating a countdown timer for stimulus offset whose duration was determined by the selected stimulus condition. Individual flies contributed a minimum of 15 events (5 events each for control and two experimental conditions) and a maximum of 20 events (5 events each for control and all three experimental conditions) to the final data set. At the beginning and end of a behavioral session, 20-s light pulses were delivered at 90-s intervals to examine the impact of photostimulation outside the context of burrowing. The aberrant persistence of burrowing and/or reversion in the behavioral sequence following egg expulsion were reliably observed using the second PU split-GAL4 line in response to 5-s stimulation at 8 µW mm^–2^ (PU-2>CsChrimson, 60/60, *n* = 12). Genetic control flies did not exhibit these aberrant behaviors in response to 5 s of stimulation at 8 µW mm^–2^ (PU-1>smGFP, 0/50, *n* = 10 flies; empty-split-GAL4>CsChrimson, 0/50, *n* = 10).

For the optogenetic stimulation of CMU neurons in gravid females, up to 12 flies were transferred to a small, circular acrylic chamber (28 mm in diameter and 2 mm in height) and placed atop an infrared panel light (850 nm; Smart Vision Lights). Video recordings were performed using a USB3 camera (Basler Ace acA2040-90umNIR) attached to a ×0.377 telecentric lens (Edmund Optics, 34-015) at 40 Hz (2,048 × 2,048 pixels). Photostimulation was controlled by a custom MATLAB GUI and delivered by four 655-nm LEDs. A single volley of five light pulses of the selected duration was delivered with 1 s between pulse offset and onset, and the fraction of flies that expelled an egg at any point before 4 s following the last pulse offset was scored.

For the optogenetic silencing of CMU neurons during egg-laying behavior, all experiments were performed in the assembly used for high-resolution filming. Flies were filmed under constant green light (6 μW, 530 nm) delivered by the off-axis ring light.

### Wild-type and loss-of-function behavior data analysis

For the high-resolution assay, the video was segmented in one of three ways. Wild-type flies on 1% agarose and PU-silenced flies were analyzed over ±60 s of egg expulsion. Wild-type flies on substrates of various firmness and ATB-silenced flies were analyzed over a contiguous video segment spanning three to eight egg-laying events beginning immediately after the first egg was deposited. CMU-silenced flies were analyzed over the entire 2-h recording session.

Segmented videos were manually annotated frame by frame in a custom MATLAB GUI. Manual scoring of behavior required 640.5 ± 275.1 s (mean ± s.d.) per 2-min video (*n* = 25 videos). Detailed annotation criteria are provided in Supplementary Table 1. The timing and count of burrow cycles were determined by observing individual episodes in real time. The cycle count per burrowing episode was highly positively correlated with the duration of the episode (*r* = 0.91). Manual annotations were validated by independently rescoring behavior on a subset of data using a second human annotator and were also compared to the output of a supervised learning algorithm (DeepEthogram^26^). Agreement was determined by calculating the F1 score^25,26^, a standard metric that ranges from 0 (poor agreement) to 1 (perfect agreement), calculated as

$${{{\mathrm{F1}}}}\;{{{\mathrm{score}}}} = \left( {{{{\mathrm{2}}}} \ast {{{\mathrm{precision}}}} \ast {{{\mathrm{recall}}}}} \right)/\left({{{\mathrm{precision}}}} + {{{\mathrm{recall}}}}\right),$$

with

$${{{\mathrm{precision}}}} = {{{\mathrm{true}}}}\;{{{\mathrm{positive}}}}/({{{\mathrm{true}}}}\;{{{\mathrm{positive}}}} + {{{\mathrm{false}}}}\;{{{\mathrm{positive}}}})$$

and

$${{{\mathrm{recall}}}} = {{{\mathrm{true}}}}\;{{{\mathrm{positive}}}}/({{{\mathrm{true}}}}\;{{{\mathrm{positive}}}} + {{{\mathrm{false}}}}\;{{{\mathrm{negative}}}}).$$

DeepLabCut (DLC; a feature detection algorithm)^28^ was used to track the *x*–*y* position of the posterior tip of the scutellum on the thorax, and the speed was estimated by comparing the distance between this position across ten frames (500 ms). A fly was considered to be walking if its speed, smoothed by a 1-s moving average, was greater than a threshold of 0.29 mm s^–1^.

For the determination of behavioral transition probabilities, the probability of start-to-start transitions was calculated as in ref. ^24^. Proboscis extension was only considered when expressed at distinct locations spaced greater than 500 μm apart. Proboscis extension events that occurred during other behaviors were omitted from this analysis. Bend onset was only scored once if burrowing was aborted and then reinitiated during a sustained abdominal bend. Transition probabilities were determined separately for behaviors happening before and after egg expulsion (egg out). The statistical significance of each transition was determined by comparison to a distribution of transition probabilities derived from 10,000 shuffled permutations of the original sequences. Transitions not shown were not significant and of low probability (<0.04; initial behavior distribution and complete transition matrices are shown in Supplementary Table 2).

For quantifying normalized egg depth of penetration, an egg was given a score of 1 if it was deposited entirely beneath the substrate surface, with only the egg’s spiracles exposed. If only part of the egg was beneath the surface, it was given a score of 0.5, whereas if it was entirely resting on the surface, it was scored as 0. The average normalized egg depth was calculated per fly for all flies that laid one or more eggs.

### Supervised behavioral classification analysis

A DeepEthogram-slow model^26^ was trained using 518 manually annotated 2-min videos surrounding egg-laying events (±1 min of completed egg expulsion). Test data consisted of a subset of 30 randomly selected 2-min videos corresponding to 72,000 frames, which were held out from the training data set. Proboscis extension and egg out labels were expanded from one frame to three frames in both train and test datasets.

### Unsupervised behavioral classification analysis

Our approach was based on ref. ^27^ and implemented using custom MATLAB code. Using key points from a DLC pose estimation model, 17 features were extracted from each video frame that represent postural and motion features relevant to egg laying. These features were velocity (movement of the scutellum over time, ‘vel’; Supplementary Video 2), movement of the proboscis relative to the ocellus (‘pe’), the *z*-score-normalized angle formed between a line connecting the ventral abdominal stripes and a line connecting the ocellus and scutellum (‘ba’), the angular velocity of this angle (‘velba’), movement of the leg joint from each leg (three features; ‘T1’–‘T3’), the DLC prediction confidence for egg emergence (‘Pegg’), the magnitude of two bands of the Morlet wavelet spectrogram of the pixel intensity of a circular region of interest (ROI) of radius 10 pixels surrounding the ovipositor (0.8 to 1.3 Hz and 1.3 to 2.3 Hz; ‘w1ovi’ and ‘w2ovi’) and the log of the magnitude of seven bands of the Morlet wavelet spectrogram of the movement of the dorsal arch of the stripe on abdominal segment A5 (ranging from 0.5 Hz to 10 Hz; ‘cwt1’–‘cwt7’). For a complete description of this analysis, see Supplementary Methods.

### Functional imaging data analysis

Imaging data were first segmented into cell-specific ROIs. The location and shape of ROIs corresponding to all labeled axons across all frames was determined from the tdTomato channel using a semiautomated pipeline. DLC was used to track the center position of all identified axons, appearing as ellipsoids, in the tdTomato image stack. DLC predictions were then used to select foreground ROIs from a binary thresholded image stack. The raw fluorescence, *F*, was then calculated as the mean pixel value within the ROI bounds for each frame. The raw fluorescence was converted to Δ*F*/*F*_0_ using a baseline determined as the median fluorescence value from recording onset to 20 s before completed egg expulsion, excluding ±20 s surrounding ovipositor extrusion events. Example Δ*F*/*F*_0_ traces shown in figures and videos were smoothed by a three-point moving average.

The timing of egg expulsion events and ovipositor extrusion events was determined via an automated analysis of the distances between DLC-tracked key points (the dorsal–posterior edge of T6, the posterior tip of the egg and the midpoint of the ovipositor). T6:egg distance and T6:ovipositor distance were used to detect behavioral events before and after egg expulsion, respectively.

Raw distance traces were high-pass filtered (0.001 Hz) before total variation regularization, with events identified as threshold crossings of one-fifth the maximum regularized signal. Distances were within-fly normalized by the median distance between T6 and the posterior edge of the analia base, which was set to 1. Calcium response events (Supplementary Fig. 4) were determined similarly.

To compare response magnitude across events and flies, the fluorescence data were integrated and normalized as follows. The integration window for both egg expulsion and ovipositor extrusion events was defined as *t* = 0 to *t* = 3 s after event onset. For comparing incomplete to complete egg expulsion, the baseline 0 value was determined as the median 3-s integral over the first contiguous stretch of 60 s leading up to complete egg expulsion, excluding ±20 s surrounding any egg expulsion event. The postexpulsion value was determined as the median 3-s integral from *t* = 10 to *t* = 20 s after complete egg expulsion. The maximum ‘1’ value for normalization was the maximum 3-s integral observed throughout. For comparing incomplete egg expulsion to postexpulsion ovipositor extrusion events, the minimum ‘0’ value was determined as the median of the first 60 s (non-contiguous) starting 10 s after egg expulsion and excluding ±20 s surrounding ovipositor extrusion events. For flies that expressed multiple events, the mean was used in plots and all analyses. Calcium imaging was performed using both PU-1 and PU-2 split-GAL4 lines, and the data were combined.

### Optogenetic activation data analysis

For every egg-laying event, the timing of completed egg expulsion and burrow termination was manually annotated in a custom MATLAB GUI. The time of completed egg expulsion was defined as the first frame where the egg reached maximum depth within the substrate. Burrow termination was defined as the frame associated with the onset of ovipositor detachment or lifting. The transition to the reset phase was determined if no additional burrowing episode occurred within 65 s of egg expulsion. If additional burrowing episodes did occur, the onset timing of the last burrowing episode before reset was similarly determined as the last burrowing episode to precede a 65-s window free of burrowing. For stimulation events presented in Fig. 7c and Extended Data Fig. 7b, *t* = 0 corresponds to the onset of the countdown timer, which approximately coincided with completed egg expulsion.

### Reporting summary

Further information on research design is available in the Nature Portfolio Reporting Summary linked to this article.

## Online content

Any methods, additional references, Nature Portfolio reporting summaries, source data, extended data, supplementary information, acknowledgements, peer review information; details of author contributions and competing interests; and statements of data and code availability are available at 10.1038/s41593-023-01332-5.

## Supplementary information

Supplementary Information Supplementary Figs. 1–6, Tables 1–8 and Methods.

Reporting Summary

Supplementary Video 1 Annotated behaviors surrounding an egg-laying event. Top: video recording of a female within an egg-laying chamber. Bottom: concurrent behavioral annotations. Blue, proboscis extension; white, walk; green, bend; magenta, burrow; yellow, detach; gray, groom. The vertical dashed line indicates the current time displayed in video; *t* = 0, completed egg expulsion (egg out).

Supplementary Video 2 Manual and automated analysis of egg-laying behavior. **i**, Video recording of a female within an egg-laying chamber displaying relevant key points used to determine features for unsupervised behavioral classification analysis (Extended Data Fig. 2 and Methods). Cyan circle, proboscis tip; red circle, dorsal arch of the stripe on abdominal segment A5; pink circle, ROI used to determine ovipositor pixel intensity in **vii**; white circle, T3 (metathoracic) leg joint; green lines, used to determine abdominal bend angle in **v**, with the upper green line connecting the ocellus to the posterior tip of the thoracic scutellum and the lower green line connecting the ventral-most edge of the stripes on abdominal segments A2 and A6. **ii**, Manual annotations. **iii**, Velocity (black, ‘vel’; 1/20× pixels per s) and proboscis movement (blue, ‘PE’; pixels per s). **iv**, Movement of the leg joint from each leg (magenta, ‘T1’; brown, ‘T2’; gray, ‘T3’; pixels per s). **v**, *Z*-score-normalized abdominal bend angle (>0 is downward bending, ‘ba’). **vi**, Egg emergence (DLC prediction confidence, ‘Pegg’). **vii**, Pixel intensity in a circular region of interest surrounding the ovipositor (1/1,000× intensity; pink circle in **i**). **viii**, Magnitude of continuous Morlet wavelet transform of ovipositor intensity trace in **vii**. The ovipositor intensity trace displays oscillations that slow in frequency as the egg incrementally emerges and is completely expelled. **ix**, Log magnitude of continuous Morlet wavelet transform of the position of the stripe on abdominal segment A5 (red circle in **i**; same data depicted in Supplementary Fig. 3).

Supplementary Video 3 Unsupervised behavioral clusters encompassing egg-laying behavior. Five-second video clips of flies displaying representative actions associated with each of the 14 t-SNE clusters that occurred significantly above chance and displayed peak expression within ±20 s of egg out (Extended Data Fig. 2c,d,f and Methods). Four clips are presented for each cluster. Black text at top left, exemplar cluster identity; gray index at center, real-time cluster expression; white index at center, midpoint of exemplar behavioral epoch after which the 5-s clip is centered. Clusters are presented according to their peak timing (Extended Data Fig. 2c).

Supplementary Video 4 PU neurons respond to the progression of the egg into the ovipositor. **a**, Two-photon imaging depicting relative fluorescence changes of GCaMP6f in four PU neurons (two each from the right and left sides) from a representative experiment encompassing an incomplete egg expulsion event followed by complete egg expulsion over a 90-s window. Measurements were performed in PU axons within the abdominal nerve trunk as shown in **c**; vertical dashed line, current time displayed in **c** and **d**. **b**, Top: normalized distance between T6 and ovipositor (blue). Bottom: normalized distance between T6 and posterior tip of the egg (magenta; Methods). Raw distance measures superimposed on video recording in **d**. **c**, Corresponding two-photon coronal section through the abdominal nerve trunk depicting raw GCaMP6f (green) and tdTomato (red) fluorescence in PU neurons (PU-1>GCaMP6f; tdTomato). Segmented ROIs corresponding to individual PU axons are color coded to match the traces in **a**; V, ventral; D, dorsal. **d**, Corresponding video recording of the posterior abdomen, lateral aspect, under infrared light. The blue and magenta lines connect the dorsal–posterior edge of T6 with the ovipositor and posterior tip of the egg, respectively. Before expulsion, the posterior aspect of the egg within the uterus is visible through the abdominal cuticle (same data depicted in Fig. 5g).

Supplementary Video 5 PU neurons do not respond to ovipositor extrusion after egg expulsion. **a**, Two-photon imaging depicting relative fluorescence changes of GCaMP6f in four PU neurons (two each from the right and left sides) from a representative experiment over two 30-s windows, one before and one after egg expulsion. The first clip (‘before egg expulsion’) encompasses multiple incomplete egg expulsion events. The second clip (‘after egg expulsion’) encompasses multiple ovipositor extrusion events lacking an egg. Measurements were performed in PU axons within the abdominal nerve trunk, as shown in **c**; vertical dashed line, current time displayed in **c** and **d**. **b**, Top: normalized distance between T6 and ovipositor (blue). Bottom: normalized distance between T6 and posterior tip of the egg (magenta; Methods). Raw distance measures superimposed on the video recording in **d**. **c**, Corresponding two-photon coronal section through the abdominal nerve trunk depicting raw GCaMP6f (green) and tdTomato (red) fluorescence in PU neurons (PU-1>GCaMP6f; tdTomato). Segmented ROIs corresponding to individual PU axons are color coded to match the traces in **a**; V, ventral; D, dorsal. **d**, Corresponding video recording of the posterior abdomen, lateral aspect, under infrared light. The blue and magenta lines connect the dorsal–posterior edge of T6 with the ovipositor and posterior tip of the egg, respectively. Before expulsion, the posterior aspect of the egg within the uterus is visible through the abdominal cuticle (same data depicted in Fig. 5j).

Supplementary Video 6 PU photostimulation beyond egg expulsion drives burrowing persistence. Four representative 35-s videos of PU-1>CsChrimson flies filmed within egg-laying chambers under infrared light exposed to different durations of red-light stimulation beyond completed egg expulsion. The ‘no-light’ control is at the top, and three experimental conditions (2.5-s, 5-s and 20-s light) are below. For each experimental condition, flies persist in burrowing throughout photostimulation and, after light offset, abruptly stop burrowing and transition to the behavioral sequence normally triggered by egg expulsion (‘advance’ group in Fig. 5). The light stimulation period indicated by the presence of a red box in the top left corner that, following completed egg expulsion, diminishes in size to reflect the stimulation time that remains. The background flashes white to indicate the timing of egg expulsion (*t* = 0.0 s).

Supplementary Video 7 Distinct phenotypes induced by PU photostimulation beyond egg expulsion. Eight representative 35-s videos of PU-1>CsChrimson flies filmed within egg-laying chambers under infrared light exposed to 5 s of red-light stimulation beyond completed egg expulsion. The first cohort of four videos depicts ‘advance’ events where flies persist in burrowing throughout photostimulation and, after light offset, abruptly stop burrowing and transition to the behavioral sequence normally triggered by egg expulsion. The second cohort of four videos depicts ‘revert’ events where flies stop burrowing before photostimulation offset and subsequently exhibit exploration and deposition behaviors in new locations despite the fact that they had already expelled an egg. The light stimulation period is indicated by the presence of a red box in the top left corner that, following completed egg expulsion, diminishes in size to reflect the stimulation time that remains. For the ‘revert’ cohort, the red stimulus box changes to yellow after burrow termination. The background flashes white to indicate the timing of stimulus-countdown trigger (*t* = 0.0 s; approximately concurrent with egg expulsion). In the final segment, both cohorts are displayed simultaneously (video playback speed 2× throughout).

## Data Availability

Data from this study are available at https://github.com/axellaboratory/Cury_and_Axel_2023 and upon request. Trained pose estimation models and the supervised behavioral classifier can be accessed via Dropbox (https://www.dropbox.com/sh/jh4422f3ld95j1a/AAAHVb-pFsmcEk40BgSHm1TEa?dl=0).

## Code availability

Code used for processing the data is available at https://github.com/axellaboratory/Cury_and_Axel_2023.
