# Supplementary material for: Flexible neural control of transition points within the egg-laying behavioral sequence in Drosophila
Source: Nat Neurosci. 2023 May 22;26(6):1054–67. doi: 10.1038/s41593-023-01332-5 (PMC10244180; doi:10.1038/s41593-023-01332-5)
Supplement: Supplementary file 1 — Supplementary Figs. 1–6, Tables 1–8 and Methods. [file 41593_2023_1332_MOESM1_ESM.pdf]

nature neuroscience

Article

https://doi.org/10.1038/s41593-023-01332-5

Flexible neural control of transition points within the egg-laying behavioral sequence in *Drosophila*

In the format provided by the authors and unedited

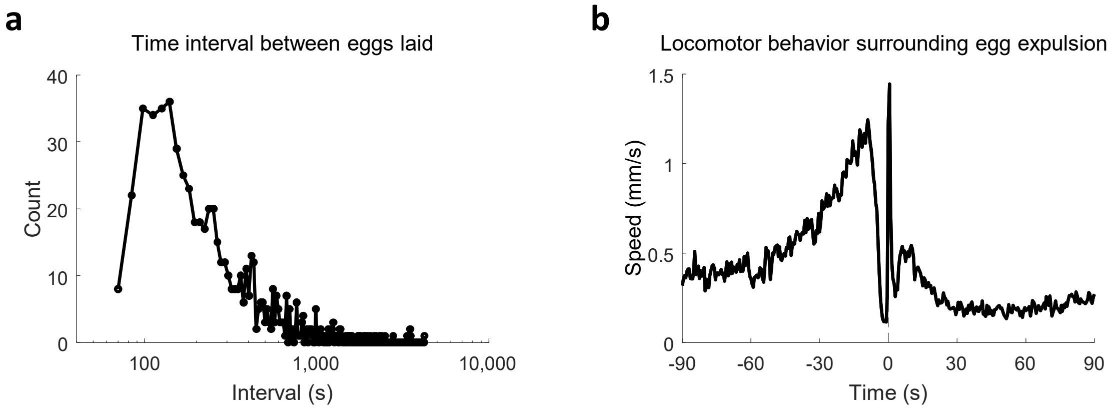

Supplementary Fig. 1 | Interval and locomotor behavior of egg-laying cycle.
a, Distribution of time interval between successive egg-laying events observed over 3 h (n = 669 eggs from 32 flies). Single females were filmed in parallel within a laser-cut acrylic assembly containing four chambers (10 mm x 18 mm x 16 mm). Substrate comprised of 1% agarose and 5% acetic acid (vol/vol) was poured into each chamber and allowed to set for 30 minutes before females were introduced by gentle aspiration. The assembly was placed atop a red led panel light (Advance Illumination) for illumination, and adjacent to a mirror positioned at a 45° angle, allowing for the flies to be simultaneously filmed from the top and side perspective. Video recording was performed using a USB3 camera (FL3-U3-13Y3M-C, Point Grey) attached to a ×6 macro zoom lens (Edmund Optics #68-667) at 2 Hz (262 x 445 x 390 pixels per chamber) via FlyCapture software (Point Grey). **b**, Average speed of flies over a 180-s window surrounding completed egg expulsion (egg out). $t = 0$, egg out. The speed surrounding egg expulsion was determined by comparing the distance between the fly's 3-dimensional center-of-mass across successive frames (500 ms).

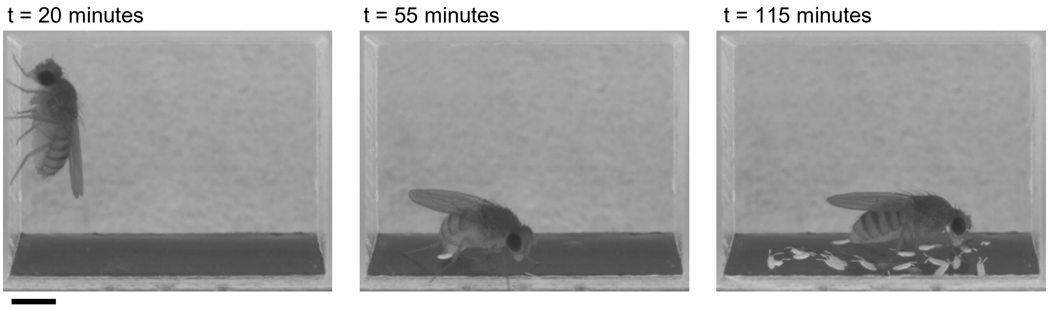

Supplementary Fig. 2 | High-resolution egg-laying behavioral assay.
Representative video snapshots of an individual chamber from egg-laying behavioral assay at three time points. Scale bar, 1 mm.

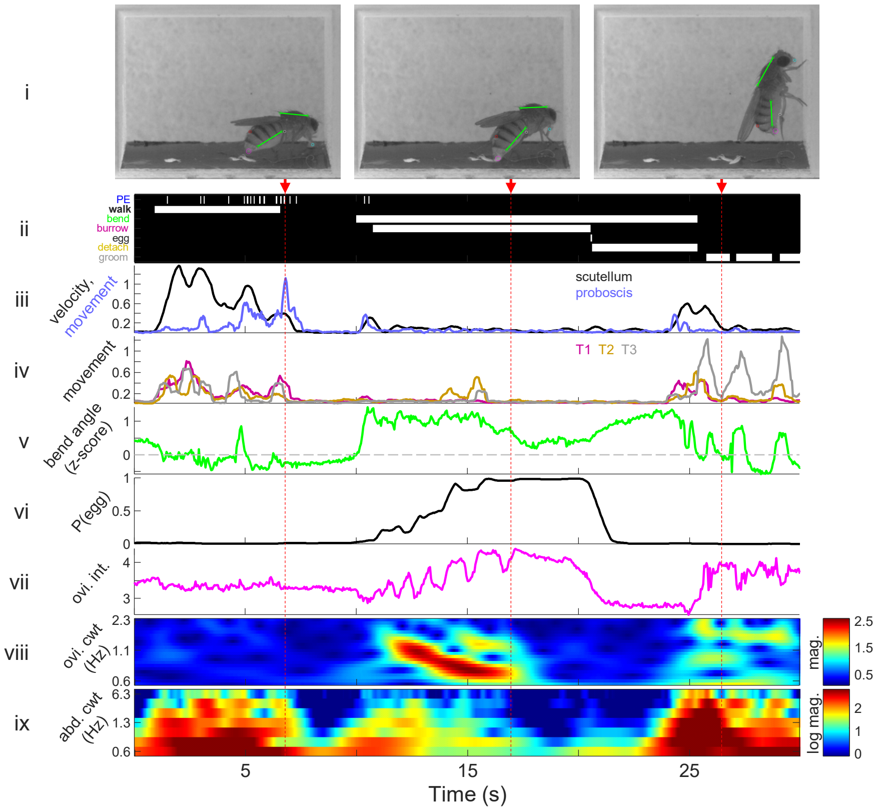

Supplementary Fig. 3 | Manual and automated analysis of egg-laying behavior.
i, Video snapshots displaying relevant key-points used to determine features for unsupervised behavioral classification analysis (in Extended Data Fig. 2; Methods). Cyan circle, proboscis tip; red circle, dorsal arch of the stripe on abdominal segment A5; pink circle, region of interest used to determine ovipositor pixel intensity in **vii**; white circle, T3 (metathoracic) leg joint; green lines, used to determine abdominal bend angle in **v**, with the upper green line connecting the ocellus to the posterior tip of the thoracic scutellum and the lower green line connecting the ventral-most edge of the stripes on abdominal segments A2 and A6. Red arrows and vertical dashed lines, corresponding time point for each video snapshot in the plots below. **ii**, Manual annotations. **iii**, Velocity (black, 'vel'; 1/20× pixels per s) and proboscis movement (blue, 'pe'; pixels per s). **iv**, Movement of leg joint from each leg (magenta, 'T1'; brown, 'T2'; gray, 'T3'; pixels per s). **v**, Z-score normalized abdominal bend angle (>0 is downward bending, 'ba'). **vi**, Egg emergence (DeepLabCut prediction confidence, 'Pegg'). **vii**, Pixel intensity in a circular region of interest surrounding the ovipositor (1/1,000× intensity; pink circle in **i**). **viii**, Magnitude of continuous Morlet wavelet transform of ovipositor intensity trace in **vii**. The ovipositor intensity trace displays oscillations that slow in frequency as the egg incrementally emerges and is completely expelled. **ix**, Log magnitude of continuous Morlet wavelet transform of the position of the stripe on abdominal segment A5 (red circle in **i**; same data are displayed in Supplementary Video 2).

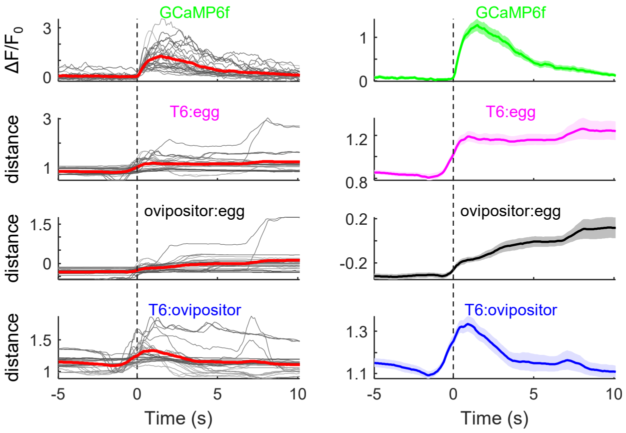

Supplementary Fig. 4 | Imaging and behavioral data aligned to PU calcium-response events. Left: average time course of GCamMP6f relative fluorescence changes ($\Delta F/F_0$, top plot; n = 28 time courses from 28 neurons in 8 flies) and behavioral measurements (bottom three plots; n = 8 flies) aligned to PU calcium-response events (Methods); grey, average time course for each individual fly; red, mean of the per-fly average time courses. Right: mean and s.e.m. of the per-fly average time courses. $t = 0$, onset of calcium response events.

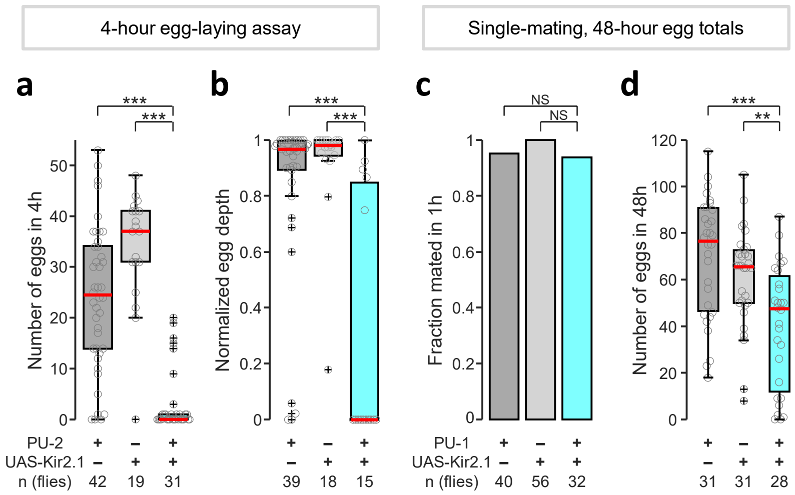

Supplementary Fig. 5 | Egg-laying deficits in PU-2>Kir2.1 and after a single mating event. **a**, Number of eggs released on a 1% agarose substrate in 4 h (n = 42, 19, 31 flies per group). Here and in **b** and **d**, box bounds, 25th and 75th percentile; red line, median; whiskers, 5th and 95th percentile; o, data from individual flies; +, outliers; *p<0.05, **p<0.01, ***p<0.001, two-sided Wilcoxon rank sum test followed by Bonferroni correction (Supplementary Table 7). **b**, Average normalized depth of penetration of eggs released on a 1% agarose substrate (n = 39, 18, 15 flies per group). **c**, Fraction of virgin females that copulated within one hour. NS, p>0.05, two-sided Fisher's exact test (n = 40, 56, 32 flies per group). For the assessment of virgin receptivity and egg-laying after a single mating, experiments were performed similarly as described before (Feng et al., *Neuron* 83, 135-148 (2014)). Receptivity was scored over a one-hour period. For egg-laying, females were transferred to small chambers (9.5 mm x 8 mm x 10 mm) containing cornmeal-agar-molasses food after the one-hour mating period and again 24 h later. Eggs were counted at 24-h and 48-h post-mating, and combined. **d**, Number of eggs released in 48 h following a single mating event (n = 31, 31, 28 flies per group).

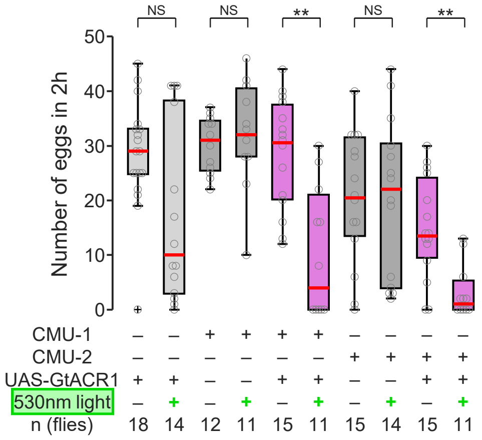

Supplementary Fig. 6 | Optogenetic inhibition of CMU neurons reduces egg output. Number of eggs released on a 1% agarose substrate in 2 h with and without constant green-light photo-inhibition (530 nm, 6 µw/mm^2 intensity; n = 18, 14, 12, 11, 15, 11, 15, 14, 15, 11 flies per group). **p<0.01, NS, p>.05, two-sided Wilcoxon rank sum test (Supplementary Table 7).

Supplementary Table 1 | Behavioral annotation scoring criterion.

Behavior	Criterion
PE	Scored as a single frame upon initial contact of the labellum with the substrate or chamber walls.
bend	Onset scored at the initiation of abdominal bends that result in the ovipositor coming in close proximity to the substrate. Offset scored as the lifting of the ovipositor and straightening of the abdomen. Abdominal bends resulting in defecation or occurring during grooming were not scored. Static, sustained abdominal bends that occurred without “burrow” (see below) were not scored.
burrow	Scored during “bend” whereupon the ovipositor is pressed against the substrate or chamber wall, and rhythmic contractions ensue. Depending on the viewing angle and background, these rhythmic contractions were observed to include rhythmic intensity changes of the ovipositor, rhythmic opening and closing of the vaginal plates, rhythmic progression of the egg out of the ovipositor, and/or rhythmic swaying anteriorly and posteriorly of all appendages. Burrow offset was scored as the termination of rhythmic contractions and lifting or sliding of the ovipositor, or upon completed egg expulsion (“egg out”, see below).
“egg out”	If the egg was completely expelled during “burrow” (see above), scored as the first frame observed where the egg occupied its final resting place. If the egg was spontaneously expelled without “burrow” (a “spontaneously dropped” egg), scored as the first frame where the egg is observed to be fully emerged from the ovipositor.
detach	Scored during “bend”, immediately after "egg out", for as long as the fly maintained a bent abdominal posture, following the criteria established for “bend” offset (see above).
groom	Scored during all varieties of inter-appendage contact involving the legs. Onset was the time of leg lifting off the substrate or chamber wall, and offset was the time at which all legs were in contact with the substrate or chamber wall.

Supplementary Table 2 | Start-to-start transition matrices, including starting behavior.

a. Starting behavior distribution

	PE	walk	bend	burrow	"egg out"	detach	groom
	0.494	0.324	0.051	0.006	0.006	0	0.119

b. Start-to-start transition probabilities for behaviors occurring BEFORE "egg out"

	PE	walk	bend	burrow	"egg out"	detach	groom
**PE**	0.310	0.630	0.044	0	0	0	0.017
**walk**	0.710	0.155	0.101	0	0	0	0.034
**bend**	0.063	0.139	0.026	0.766	0	0	0.007
**burrow**	0.056	0.126	0.026	0.134	0.651	0	0.007
**"egg out"**	-	-	-	-	-	-	-
**detach**	-	-	-	-	-	-	-
**groom**	0.214	0.268	0.021	0	0	0	0.498

c. Start-to-start transition probabilities for behaviors occurring AFTER "egg out"

	PE	walk	bend	burrow	"egg out"	detach	groom
**PE**	0.390	0.426	0	0	0	0	0.184
**walk**	0.502	0.153	0	0	0	0	0.345
**bend**	-	-	-	-	-	-	-
**burrow**	-	-	-	-	-	-	-
**"egg out"**	0	0	0	0	0	0.994	0.006
**detach**	0.069	0.063	0	0	0	0	0.869
**groom**	0.155	0.174	0	0	0	0	0.672

d. Significance (*p*-value) of transitions occurring BEFORE "egg out" (one-sided permutation test)

	PE	walk	bend	burrow	"egg out"	detach	groom
**PE**	1	0	0.0005	1	1	1	1
**walk**	0	1	0	1	1	1	1
**bend**	1	1	0.8538	0	1	1	1
**burrow**	1	1	0.9988	0	0	1	1
**"egg out"**	-	-	-	-	-	-	-
**detach**	-	-	-	-	-	-	-
**groom**	1	0.3438	1	1	1	1	0

e. Significance (*p*-value) of transitions occurring AFTER "egg out" (one-sided permutation test)

	PE	walk	bend	burrow	"egg out"	detach	groom
**PE**	1	0	1	1	1	1	0
**walk**	0	1	1	1	1	1	0
**bend**	-	-	-	-	-	-	-
**burrow**	-	-	-	-	-	-	-
**"egg out"**	1	1	1	1	1	0	1
**detach**	1	1	1	1	1	1	0
**groom**	1	1	1	1	1	1	0

Supplementary Table 3 | Terminalia bristle innervation by ATB splitGAL4 labeled neurons.

	**7th sternite** (hemi)	**7th tergite** (unilateral)	**8th tergite (epigynium*)** (unilateral)	**Hypogynial teeth*** (unilateral)	**Hypogynial long sensillum*** (unilateral)	**Hypogynial short sensilla*** (unilateral)	**Dorsal anal plate (epiproct*)**	**Ventral anal plate (hypoproct*)**
**bristle count**	5.3 ± 1.0	30.7 ± 3.7	3.4 ± 1.1	12.9 ± 0.5	1.0 ± 0.0	3.0 ± 0.0	17.9 ± 0.4	18.0 ± 0.0
**ATB-1** % (n)	94 ± 13 (5)	95 ± 2 (3)	100 ± 0 (5)	98 ± 3 (5)	100 ± 0 (5)	0 ± 0 (6)	67 ± 16 (2)	89 ± 16 (2)
**ATB-2** % (n)	100 ± 0 (6)	75 ± 9 (3)	0 ± 0 (10)	75 ± 9 (8)	100 ± 0 (8)	87 ± 30 (5)	65 ± 12 (5)	96 ± 6 (6)

* categorized as in McQueen et al., *Fly*, 16:1, 128-151 (2022)

Supplementary Table 4 | Expression data for CMU splitGAL4 lines.

	**Axons innervating uterus (genital chamber)** (bilateral)	**Abdominal neuromere descending neurons**	**Abdominal neuromere local neurons**	**Abdominal nerve trunk processes**
**CMU-1** (n)	1.8 ± 0.5 (8)	2.1 ± 0.4 (8)	1.4 ± 0.9 (8)	2.0 ± 0.5 (8)
**CMU-2** (n)	2.0 ± 0.0 (11)	3.8 ± 0.4 (22)	0.0 ± 0.2 (22)	3.9 ± 0.5 (22)
**CMU-3** (n)	2.0 ± 0.0 (2)	3.5 ± 0.8 (6)	2.0 ± 1.1 (6)	4.2 ± 1.5 (6)
**CMU-4** (n)	2.0 ± 0.0 (10)	4.3 ± 1.5 (12)	4.3 ± 2.9 (12)	4.7 ± 1.6 (12)

Supplementary Table 5 | Fly stocks and sources.

**Fly Genotype**	**Source**
Canton-S	Gift of Barry Dickson
2U	Gift of Barbara Noro
UAS-IVS-mCD8-GFP in VK00005	Gift of Gerry Rubin
UAS-IVS-myr-smGFP in VK00005	Gift of Gerry Rubin
UAS-Kir2.1-T2A-tdTomato in VK00005	Gift of Daisuke Hattori
UAS-RedStinger (X chromosome)	Bloomington Stock Center, No. 8545
UAS-IVS-GCaMP6f in attp40	Bloomington Stock Center, No. 42747
UAS-tdTomato in VK00005	Gift of Gerry Rubin
UAS(FRT.stop)CsChrimson-mVenus in attp18	Gift of Barry Dickson
UAS-DenMark, UAS-syt-GFP	Bloomington Stock Center, No. 33064
UAS-CsChrimson-tdTomato in VK00005	Hoopfer et al., *eLife* 4, e11346 (2015)
UAS-mCD8-GFP (second chromosome)	Lee and Luo, *Neuron* 22, 451-461 (1999)
UAS-CsChrimson-mVenus in attp18	Bloomington Stock Center, No. 55134
UAS-GtACR1-T2A-tdTomato in attp8	Gift of Barbara Noro
UAS-IVS-mCD8-GFP in attp8	Bloomington Stock Center, No. 32189
UAS(FRT.mCherry)Kir2.1-GFP	Gift of Troy Shirangi
ATB-1 splitGAL4 p65AD (R38B08-p65ADZp in attp40)	Bloomington Stock Center, No. 68637
ATB-2 splitGAL4 p65AD (VT037601-p65ADZp in attp40)	Bloomington Stock Center, No. 74486
ATB-1 and ATB-2 splitGAL4 ZpG4DBD (*dsx*-G4DBD)	Pavlou et al., *eLife* 5, e20713 (2016)
PU-1 and PU-2 splitGAL4 p65AD (R12A10-p65ADZp in attp40)	Bloomington Stock Center, No. 68819
PU-1 splitGAL4 G4DBD (R9D10-ZpG4DBD in attp2)	This paper
PU-2 splitGAL4 G4DBD (R28H10-ZpG4DBD in attp2)	This paper
CMU-1 splitGAL4 p65AD (R71D08-p65ADZp in attp40)	Bloomington Stock Center, No. 70796
CMU-2 splitGAL4 p65AD (R65C02-p65ADZp in attp40)	Bloomington Stock Center, No. 71006
CMU-3 splitGAL4 p65AD (R55D12-p65ADZp in attp40)	Bloomington Stock Center, No. 75942
CMU-4 splitGAL4 p65AD (R51G06-p65ADZp in attp40)	This paper
CMU-1, CMU-3, and CMU-4 splitGAL4 G4DBD (R70D11-ZpG4DBD in attp2)	This paper
CMU-2 splitGAL4 G4DBD (R55D12-ZpG4DBD in attp2)	Bloomington Stock Center, No. 68775
empty-splitGAL4 (pBPp65ADZp in attP40; pBPZpGDBD in attP2)	Bloomington Stock Center, No. 79603
Otd-nls:FLP	Asahina et al., *Cell* 156, 221–235 (2014).
yw, hs-FLP	Gordon and Scott, *Neuron* 61, 373–384 (2009)

Supplementary Table 6 | Fly genotypes by figure.

Figure	Fly Genotype
Figures 1, 2, 4, Extended Data Figures 1-3, Supplementary Figures 1-3, and Supplementary Videos 1-3	Canton-S
Figure 3c-e and Extended Data Figure 4c	+/+; R38B08-p65AD/+; dsx-G4DBD/UAS-IVS-mCD8-GFP
Figure 3f-k and Extended Data Figure 5a-b	+/+; R38B08-p65AD/+; dsx-G4DBD/UAS-IVS-myr-smGFP
Figure 3f-k and Extended Data Figures 4d, 5a-b	+/+; R38B08-p65AD/+; dsx-G4DBD/UAS-Kir2.1-T2A-tdTomato
Figures 3f-k, 6, Extended Data Figure 5 and Supplementary Figure 5	+/+; pBPp65AD/+; pBPGDBD/UAS-Kir2.1-T2A-tdTomato
Extended Data Figure 4a-c	+/+; VT037601-p65AD/+; dsx-G4DBD/UAS-IVS-mCD8-GFP
Extended Data Figure 5c-d	+/+; VT037601-p65AD/+; dsx-G4DBD/UAS-IVS-myr-smGFP<br>+/+; VT037601-p65AD/+; dsx-G4DBD/UAS-Kir2.1-T2A-tdTomato
Extended Data Figure 6	+/+; R38B08-p65AD/+; dsx-G4DBD/ UAS(FRT.mCherry)Kir2.1-GFP<br>+/+; pBPp65AD/Otd-nls:FLP; pBPGDBD/ UAS(FRT.mCherry)Kir2.1-GFP<br>+/+; R38B08-p65AD/Otd-nls:FLP; dsx-G4DBD/ UAS(FRT.mCherry)Kir2.1-GFP
Figure 5a-c	UAS-RedStinger; R12A10-p65AD/+; R9D10-G4DBD/UAS-IVS-mCD8-GFP
Figure 5e and Extended Data Figure 7b	+/+; R12A10-p65AD/+; R9D10-G4DBD/UAS-IVS-mCD8-GFP
Figure 5f,g,j, Supplementary Figure 4, and Supplementary Videos 4, 5	+/+; R12A10-p65AD/UAS-GCaMP6f; R9D10-G4DBD/UAS-tdTomato
Figure 5h,i,k,l	+/+; R12A10-p65AD/UAS-GCaMP6f; R9D10-G4DBD/UAS-tdTomato<br>+/+; R12A10-p65AD/UAS- GCaMP6f; R28H10-G4DBD/UAS-tdTomato
Extended Data Figure 7a,b	+/+; R12A10-p65AD/+; R28H10-G4DBD/UAS-IVS-mCD8-GFP
Extended Data Figure 7c	hs-FLP/UAS(FRT.stop)CsChrimson; R12A10-p65AD/+; R28H10-G4DBD/+
Extended Data Figure 10d	+/+; R12A10-p65AD/ UAS-DenMark, UAS-syt-GFP; R28H10-G4DBD
Figure 6 and Supplementary Figure 5c,d	+/+; R12A10-p65AD/+; R9D10-G4DBD /UAS-IVS-myr-smGFP<br>+/+; R12A10-p65AD/+; R9D10-G4DBD /UAS-Kir2.1-T2A-tdTomato
Supplementary Figure 5a,b	+/+; R12A10-p65AD/+; R28H10-G4DBD /UAS-IVS-myr-smGFP<br>+/+; R12A10-p65AD/+; R28H10-G4DBD /UAS-Kir2.1-T2A-tdTomato
Figure 7b,c, Extended Data Figures 8, 9, and Supplementary Videos 6, 7	+/+; R12A10-p65AD/+; R28H10-G4DBD /UAS-CsChrimson
Figure 8a,c and Extended Data Figure 10a	+/+; R71D08-p65AD/UAS-mCD8-GFP/+; R70D11-G4DBD/+
Figure 8d	UAS-CsChrimson; R55D12-p65AD/+; R70D11-G4DBD/+<br>UAS-CsChrimson; pBPp65AD/+; pBPGDBD/+<br>+/+; R55D12-p65AD/+; R70D11-G4DBD/+<br>+/+; R51G06-p65AD/+; R70D11-G4DBD/+
Figure 8d and Extended Data Figure 10b	UAS-CsChrimson; R51G06-p65AD/+; R70D11-G4DBD/+
Figure 8e,f	+/+; R55D12-p65AD/UAS- GCaMP6f 6f; R70D11-G4DBD/UAS-tdTomato
Figure 8g-k and Supplementary Figure 6	UAS-IVS-mCD8-GFP; R65C02-p65AD/+; R55D12-G4DBD/+<br>UAS-GtACR1; pBPp65AD/+; pBPGDBD/+<br>UAS-GtACR1; R65C02-p65AD/+; R55D12-G4DBD/+
Extended Data Figure 10a	+/+; R65C02-p65AD/UAS-mCD8-GFP; R55D12-G4DBD/+<br>+/+; R55D12-p65AD/UAS-mCD8-GFP; R70D11-G4DBD/+<br>+/+; R51G06-p65AD/UAS-mCD8-GFP; R70D11-G4DBD/+
Supplementary Figure 6	UAS-IVS-mCD8-GFP; R71D08-p65AD/+; R70D11-G4DBD/+<br>UAS-GtACR1; R71D08-p65AD/+; R70D11-G4DBD/+

Supplementary Table 7 | Statistical analyses.

Figure	Group 1	Group 2	Test	*p*-value
2a	0.75%, on surface	1.0%, on surface	Kruskal-Wallis test with post hoc Tukey's HSD test	0.994
2a	0.75%, on surface	1.25%, on surface	Kruskal-Wallis test with post hoc Tukey's HSD test	0.9996
2a	0.75%, on surface	1.5%, on surface	Kruskal-Wallis test with post hoc Tukey's HSD test	0.7013
2a	0.75%, on surface	1.75%, on surface	Kruskal-Wallis test with post hoc Tukey's HSD test	0.4456
2a	0.75%, on surface	2.0%, on surface	Kruskal-Wallis test with post hoc Tukey's HSD test	0.0001
2a	0.75%, on surface	2.25%, on surface	Kruskal-Wallis test with post hoc Tukey's HSD test	<0.0001
2a	0.75%, on surface	2. 5%, on surface	Kruskal-Wallis test with post hoc Tukey's HSD test	<0.0001
2a	1.0%, on surface	1.25%, on surface	Kruskal-Wallis test with post hoc Tukey's HSD test	1
2a	1.0%, on surface	1.5%, on surface	Kruskal-Wallis test with post hoc Tukey's HSD test	0.9829
2a	1.0%, on surface	1.75%, on surface	Kruskal-Wallis test with post hoc Tukey's HSD test	0.8935
2a	1.0%, on surface	2.0%, on surface	Kruskal-Wallis test with post hoc Tukey's HSD test	0.002
2a	1.0%, on surface	2.25%, on surface	Kruskal-Wallis test with post hoc Tukey's HSD test	<0.0001
2a	1.0%, on surface	2.5%, on surface	Kruskal-Wallis test with post hoc Tukey's HSD test	<0.0001
2a	1.25%, on surface	1.5%, on surface	Kruskal-Wallis test with post hoc Tukey's HSD test	0.9408
2a	1.25%, on surface	1.75%, on surface	Kruskal-Wallis test with post hoc Tukey's HSD test	0.7837
2a	1.25%, on surface	2.0%, on surface	Kruskal-Wallis test with post hoc Tukey's HSD test	0.001
2a	1.25%, on surface	2.25%, on surface	Kruskal-Wallis test with post hoc Tukey's HSD test	<0.0001
2a	1.25%, on surface	2.5%, on surface	Kruskal-Wallis test with post hoc Tukey's HSD test	<0.0001
2a	1.5%, on surface	1.75%, on surface	Kruskal-Wallis test with post hoc Tukey's HSD test	1
2a	1.5%, on surface	2.0%, on surface	Kruskal-Wallis test with post hoc Tukey's HSD test	0.0427
2a	1.5%, on surface	2.25%, on surface	Kruskal-Wallis test with post hoc Tukey's HSD test	<0.0001
2a	1.5%, on surface	2.5%, on surface	Kruskal-Wallis test with post hoc Tukey's HSD test	<0.0001
2a	1.75%, on surface	2.0%, on surface	Kruskal-Wallis test with post hoc Tukey's HSD test	0.1053
2a	1.75%, on surface	2.25%, on surface	Kruskal-Wallis test with post hoc Tukey's HSD test	<0.0001
2a	1.75%, on surface	2.5%, on surface	Kruskal-Wallis test with post hoc Tukey's HSD test	<0.0001
2a	2.0%, on surface	2.25%, on surface	Kruskal-Wallis test with post hoc Tukey's HSD test	0.0087
2a	2.0%, on surface	2.5%, on surface	Kruskal-Wallis test with post hoc Tukey's HSD test	<0.0001
2a	2.25%, on surface	2.5%, on surface	Kruskal-Wallis test with post hoc Tukey's HSD test	0.8561
2b	0.75% agarose	1.0% agarose	Kruskal-Wallis test with post hoc Tukey's HSD test	0.6753
2b	0.75% agarose	1.25% agarose	Kruskal-Wallis test with post hoc Tukey's HSD test	0.9997
2b	0.75% agarose	1.5% agarose	Kruskal-Wallis test with post hoc Tukey's HSD test	0.9986
2b	0.75% agarose	1.75% agarose	Kruskal-Wallis test with post hoc Tukey's HSD test	0.9821
2b	0.75% agarose	2.0% agarose	Kruskal-Wallis test with post hoc Tukey's HSD test	0.0006
2b	0.75% agarose	2.25% agarose	Kruskal-Wallis test with post hoc Tukey's HSD test	0.0033
2b	0.75% agarose	2. 5% agarose	Kruskal-Wallis test with post hoc Tukey's HSD test	<0.0001
2b	1.0% agarose	1.25% agarose	Kruskal-Wallis test with post hoc Tukey's HSD test	0.9247
2b	1.0% agarose	1.5% agarose	Kruskal-Wallis test with post hoc Tukey's HSD test	0.9614
2b	1.0% agarose	1.75% agarose	Kruskal-Wallis test with post hoc Tukey's HSD test	0.9954
2b	1.0% agarose	2.0% agarose	Kruskal-Wallis test with post hoc Tukey's HSD test	0.0892
2b	1.0% agarose	2.25% agarose	Kruskal-Wallis test with post hoc Tukey's HSD test	0.2487
2b	1.0% agarose	2.5% agarose	Kruskal-Wallis test with post hoc Tukey's HSD test	<0.0001
2b	1.25% agarose	1.5% agarose	Kruskal-Wallis test with post hoc Tukey's HSD test	1
2b	1.25% agarose	1.75% agarose	Kruskal-Wallis test with post hoc Tukey's HSD test	0.9998
2b	1.25% agarose	2.0% agarose	Kruskal-Wallis test with post hoc Tukey's HSD test	0.0033
2b	1.25% agarose	2.25% agarose	Kruskal-Wallis test with post hoc Tukey's HSD test	0.0157
2b	1.25% agarose	2.5% agarose	Kruskal-Wallis test with post hoc Tukey's HSD test	<0.0001
2b	1.5% agarose	1.75% agarose	Kruskal-Wallis test with post hoc Tukey's HSD test	1
2b	1.5% agarose	2.0% agarose	Kruskal-Wallis test with post hoc Tukey's HSD test	0.0054
2b	1.5% agarose	2.25% agarose	Kruskal-Wallis test with post hoc Tukey's HSD test	0.0240
2b	1.5% agarose	2.5% agarose	Kruskal-Wallis test with post hoc Tukey's HSD test	<0.0001
2b	1.75% agarose	2.0% agarose	Kruskal-Wallis test with post hoc Tukey's HSD test	0.0149
2b	1.75% agarose	2.25% agarose	Kruskal-Wallis test with post hoc Tukey's HSD test	0.0573
2b	1.75% agarose	2.5% agarose	Kruskal-Wallis test with post hoc Tukey's HSD test	<0.0001
2b	2.0% agarose	2.25% agarose	Kruskal-Wallis test with post hoc Tukey's HSD test	0.9999
2b	2.0% agarose	2.5% agarose	Kruskal-Wallis test with post hoc Tukey's HSD test	0.2937
2b	2.25% agarose	2.5% agarose	Kruskal-Wallis test with post hoc Tukey's HSD test	0.1252
2d	0.75% agarose	1.0% agarose	Kruskal-Wallis test with post hoc Tukey's HSD test	0.8589
2d	0.75% agarose	1.25% agarose	Kruskal-Wallis test with post hoc Tukey's HSD test	0.7427
2d	0.75% agarose	1.5% agarose	Kruskal-Wallis test with post hoc Tukey's HSD test	0.9984
2d	0.75% agarose	1.75% agarose	Kruskal-Wallis test with post hoc Tukey's HSD test	0.2476
2d	1.0% agarose	1.25% agarose	Kruskal-Wallis test with post hoc Tukey's HSD test	0.9999
2d	1.0% agarose	1.5% agarose	Kruskal-Wallis test with post hoc Tukey's HSD test	0.9806
2d	1.0% agarose	1.75% agarose	Kruskal-Wallis test with post hoc Tukey's HSD test	0.0398
2d	1.25% agarose	1.5% agarose	Kruskal-Wallis test with post hoc Tukey's HSD test	0.9554
2d	1.25% agarose	1.75% agarose	Kruskal-Wallis test with post hoc Tukey's HSD test	0.0161
2d	1.5% agarose	1.75% agarose	Kruskal-Wallis test with post hoc Tukey's HSD test	0.2525

2e	0.75% agarose	1.0% agarose	Kruskal-Wallis test with post hoc Tukey’s HSD test	0.9642
2e	0.75% agarose	1.25% agarose	Kruskal-Wallis test with post hoc Tukey’s HSD test	0.9903
2e	0.75% agarose	1.5% agarose	Kruskal-Wallis test with post hoc Tukey’s HSD test	0.0046
2e	0.75% agarose	1.75% agarose	Kruskal-Wallis test with post hoc Tukey’s HSD test	<0.0001
2e	1.0% agarose	1.25% agarose	Kruskal-Wallis test with post hoc Tukey’s HSD test	0.9993
2e	1.0% agarose	1.5% agarose	Kruskal-Wallis test with post hoc Tukey’s HSD test	0.0450
2e	1.0% agarose	1.75% agarose	Kruskal-Wallis test with post hoc Tukey’s HSD test	0.0007
2e	1.25% agarose	1.5% agarose	Kruskal-Wallis test with post hoc Tukey’s HSD test	0.0141
2e	1.25% agarose	1.75% agarose	Kruskal-Wallis test with post hoc Tukey’s HSD test	0.0001
2e	1.5% agarose	1.75% agarose	Kruskal-Wallis test with post hoc Tukey’s HSD test	0.8497
3f	GAL4-only 1.0%	GAL4-only 1.25%	two-sided Wilcoxon rank sum test	0.0953
3f	GAL4-only 1.0%	GAL4-only wall	two-sided Wilcoxon rank sum test	0.0119
3f	GAL4-only 1.0%	ATB-1>Kir2.1 1.0%	two-sided Wilcoxon rank sum test	0.8052
3f	GAL4-only 1.25%	GAL4-only wall	two-sided Wilcoxon rank sum test	0.2144
3f	GAL4-only 1.25%	ATB-1>Kir2.1 1.25%	two-sided Wilcoxon rank sum test	0.0014
3f	GAL4-only wall	ATB-1>Kir2.1 wall	two-sided Wilcoxon rank sum test	0.0002
3f	UAS-only 1.0%	UAS-only 1.25%	two-sided Wilcoxon rank sum test	0.0588
3f	UAS-only 1.0%	UAS-only wall	two-sided Wilcoxon rank sum test	0.0004
3f	UAS-only 1.0%	ATB-1>Kir2.1 1.0%	two-sided Wilcoxon rank sum test	0.8108
3f	UAS-only 1.25%	UAS-only wall	two-sided Wilcoxon rank sum test	0.0414
3f	UAS-only 1.25%	ATB-1>Kir2.1 1.25%	two-sided Wilcoxon rank sum test	0.0076
3f	UAS-only wall	ATB-1>Kir2.1 wall	two-sided Wilcoxon rank sum test	0.0005
3f	ATB-1>Kir2.1 1.0%	ATB-1>Kir2.1 1.25%	two-sided Wilcoxon rank sum test	0.595
3f	ATB-1>Kir2.1 1.0%	ATB-1>Kir2.1 wall	two-sided Wilcoxon rank sum test	0.9875
3f	ATB-1>Kir2.1 1.25%	ATB-1>Kir2.1 wall	two-sided Wilcoxon rank sum test	0.6632
3g	GAL4-only 1.0%	GAL4-only 1.25%	two-sided Wilcoxon rank sum test	0.1742
3g	GAL4-only 1.0%	GAL4-only wall	two-sided Wilcoxon rank sum test	<0.0001
3g	GAL4-only 1.0%	ATB-1>Kir2.1 1.0%	two-sided Wilcoxon rank sum test	<0.0001
3g	GAL4-only 1.25%	GAL4-only wall	two-sided Wilcoxon rank sum test	<0.0001
3g	GAL4-only 1.25%	ATB-1>Kir2.1 1.25%	two-sided Wilcoxon rank sum test	<0.0001
3g	GAL4-only wall	ATB-1>Kir2.1 wall	two-sided Wilcoxon rank sum test	0.0038
3g	UAS-only 1.0%	UAS-only 1.25%	two-sided Wilcoxon rank sum test	0.0901
3g	UAS-only 1.0%	UAS-only wall	two-sided Wilcoxon rank sum test	<0.0001
3g	UAS-only 1.0%	ATB-1>Kir2.1 1.0%	two-sided Wilcoxon rank sum test	<0.0001
3g	UAS-only 1.25%	UAS-only wall	two-sided Wilcoxon rank sum test	<0.0001
3g	UAS-only 1.25%	ATB-1>Kir2.1 1.25%	two-sided Wilcoxon rank sum test	0.0072
3g	UAS-only wall	ATB-1>Kir2.1 wall	two-sided Wilcoxon rank sum test	0.034
3g	ATB-1>Kir2.1 1.0%	ATB-1>Kir2.1 1.25%	two-sided Wilcoxon rank sum test	0.1026
3g	ATB-1>Kir2.1 1.0%	ATB-1>Kir2.1 wall	two-sided Wilcoxon rank sum test	0.0082
3g	ATB-1>Kir2.1 1.25%	ATB-1>Kir2.1 wall	two-sided Wilcoxon rank sum test	0.0001
3i	GAL4-only	ATB-1>Kir2.1	two-sided Wilcoxon rank sum test	3.80x10^{-7}
3i	UAS-only	ATB-1>Kir2.1	two-sided Wilcoxon rank sum test	3.08x10^{-6}
3j	GAL4-only 0.25%	GAL4-only 0.5%	Kruskal-Wallis test with post hoc Tukey’s HSD test	1
3j	GAL4-only 0.25%	GAL4-only 0.75%	Kruskal-Wallis test with post hoc Tukey’s HSD test	0.997
3j	GAL4-only 0.25%	GAL4-only 1.0%	Kruskal-Wallis test with post hoc Tukey’s HSD test	0.9567
3j	GAL4-only 0.25%	GAL4-only 1.25%	Kruskal-Wallis test with post hoc Tukey’s HSD test	0.9671
3j	GAL4-only 0.25%	GAL4-only 1.5%	Kruskal-Wallis test with post hoc Tukey’s HSD test	0.6599
3j	GAL4-only 0.25%	UAS-only 0.25%	Kruskal-Wallis test with post hoc Tukey’s HSD test	1
3j	GAL4-only 0.25%	UAS-only 0.5%	Kruskal-Wallis test with post hoc Tukey’s HSD test	0.9627
3j	GAL4-only 0.25%	UAS-only 0.75%	Kruskal-Wallis test with post hoc Tukey’s HSD test	0.9215
3j	GAL4-only 0.25%	UAS-only 1.0%	Kruskal-Wallis test with post hoc Tukey’s HSD test	1
3j	GAL4-only 0.25%	UAS-only 1.25%	Kruskal-Wallis test with post hoc Tukey’s HSD test	0.4721
3j	GAL4-only 0.25%	UAS-only 1.5%	Kruskal-Wallis test with post hoc Tukey’s HSD test	1
3j	GAL4-only 0.25%	ATB-1>Kir2.1 0.25%	Kruskal-Wallis test with post hoc Tukey’s HSD test	0.9992
3j	GAL4-only 0.25%	ATB-1>Kir2.1 0.5%	Kruskal-Wallis test with post hoc Tukey’s HSD test	1
3j	GAL4-only 0.25%	ATB-1>Kir2.1 0.75%	Kruskal-Wallis test with post hoc Tukey’s HSD test	0.9913
3j	GAL4-only 0.25%	ATB-1>Kir2.1 1.0%	Kruskal-Wallis test with post hoc Tukey’s HSD test	0.6424
3j	GAL4-only 0.25%	ATB-1>Kir2.1 1.25%	Kruskal-Wallis test with post hoc Tukey’s HSD test	0.2205
3j	GAL4-only 0.25%	ATB-1>Kir2.1 1.5%	Kruskal-Wallis test with post hoc Tukey’s HSD test	0.6359
3j	GAL4-only 0.5%	GAL4-only 0.75%	Kruskal-Wallis test with post hoc Tukey’s HSD test	1
3j	GAL4-only 0.5%	GAL4-only 1.0%	Kruskal-Wallis test with post hoc Tukey’s HSD test	0.9975
3j	GAL4-only 0.5%	GAL4-only 1.25%	Kruskal-Wallis test with post hoc Tukey’s HSD test	0.9983

3j	GAL4-only 0.5%	GAL4-only 1.5%	Kruskal-Wallis test with post hoc Tukey's HSD test	0.85
3j	GAL4-only 0.5%	UAS-only 0.25%	Kruskal-Wallis test with post hoc Tukey's HSD test	1
3j	GAL4-only 0.5%	UAS-only 0.5%	Kruskal-Wallis test with post hoc Tukey's HSD test	0.9983
3j	GAL4-only 0.5%	UAS-only 0.75%	Kruskal-Wallis test with post hoc Tukey's HSD test	0.9911
3j	GAL4-only 0.5%	UAS-only 1.0%	Kruskal-Wallis test with post hoc Tukey's HSD test	1
3j	GAL4-only 0.5%	UAS-only 1.25%	Kruskal-Wallis test with post hoc Tukey's HSD test	0.6378
3j	GAL4-only 0.5%	UAS-only 1.5%	Kruskal-Wallis test with post hoc Tukey's HSD test	1
3j	GAL4-only 0.5%	ATB-1>Kir2.1 0.25%	Kruskal-Wallis test with post hoc Tukey's HSD test	0.4137
3j	GAL4-only 0.5%	ATB-1>Kir2.1 0.5%	Kruskal-Wallis test with post hoc Tukey's HSD test	0.7786
3j	GAL4-only 0.5%	ATB-1>Kir2.1 0.75%	Kruskal-Wallis test with post hoc Tukey's HSD test	0.9999
3j	GAL4-only 0.5%	ATB-1>Kir2.1 1.0%	Kruskal-Wallis test with post hoc Tukey's HSD test	0.0097
3j	GAL4-only 0.5%	ATB-1>Kir2.1 1.25%	Kruskal-Wallis test with post hoc Tukey's HSD test	0.0004
3j	GAL4-only 0.5%	ATB-1>Kir2.1 1.5%	Kruskal-Wallis test with post hoc Tukey's HSD test	0.068
3j	GAL4-only 0.75%	GAL4-only 1.0%	Kruskal-Wallis test with post hoc Tukey's HSD test	1
3j	GAL4-only 0.75%	GAL4-only 1.25%	Kruskal-Wallis test with post hoc Tukey's HSD test	1
3j	GAL4-only 0.75%	GAL4-only 1.5%	Kruskal-Wallis test with post hoc Tukey's HSD test	0.9985
3j	GAL4-only 0.75%	UAS-only 0.25%	Kruskal-Wallis test with post hoc Tukey's HSD test	0.9983
3j	GAL4-only 0.75%	UAS-only 0.5%	Kruskal-Wallis test with post hoc Tukey's HSD test	1
3j	GAL4-only 0.75%	UAS-only 0.75%	Kruskal-Wallis test with post hoc Tukey's HSD test	1
3j	GAL4-only 0.75%	UAS-only 1.0%	Kruskal-Wallis test with post hoc Tukey's HSD test	1
3j	GAL4-only 0.75%	UAS-only 1.25%	Kruskal-Wallis test with post hoc Tukey's HSD test	0.9964
3j	GAL4-only 0.75%	UAS-only 1.5%	Kruskal-Wallis test with post hoc Tukey's HSD test	1
3j	GAL4-only 0.75%	ATB-1>Kir2.1 0.25%	Kruskal-Wallis test with post hoc Tukey's HSD test	0.1137
3j	GAL4-only 0.75%	ATB-1>Kir2.1 0.5%	Kruskal-Wallis test with post hoc Tukey's HSD test	0.3291
3j	GAL4-only 0.75%	ATB-1>Kir2.1 0.75%	Kruskal-Wallis test with post hoc Tukey's HSD test	1
3j	GAL4-only 0.75%	ATB-1>Kir2.1 1.0%	Kruskal-Wallis test with post hoc Tukey's HSD test	0.0018
3j	GAL4-only 0.75%	ATB-1>Kir2.1 1.25%	Kruskal-Wallis test with post hoc Tukey's HSD test	0.0001
3j	GAL4-only 0.75%	ATB-1>Kir2.1 1.5%	Kruskal-Wallis test with post hoc Tukey's HSD test	0.016
3j	GAL4-only 1.0%	GAL4-only 1.25%	Kruskal-Wallis test with post hoc Tukey's HSD test	1
3j	GAL4-only 1.0%	GAL4-only 1.5%	Kruskal-Wallis test with post hoc Tukey's HSD test	0.9998
3j	GAL4-only 1.0%	UAS-only 0.25%	Kruskal-Wallis test with post hoc Tukey's HSD test	0.9414
3j	GAL4-only 1.0%	UAS-only 0.5%	Kruskal-Wallis test with post hoc Tukey's HSD test	1
3j	GAL4-only 1.0%	UAS-only 0.75%	Kruskal-Wallis test with post hoc Tukey's HSD test	1
3j	GAL4-only 1.0%	UAS-only 1.0%	Kruskal-Wallis test with post hoc Tukey's HSD test	0.9996
3j	GAL4-only 1.0%	UAS-only 1.25%	Kruskal-Wallis test with post hoc Tukey's HSD test	0.9996
3j	GAL4-only 1.0%	UAS-only 1.5%	Kruskal-Wallis test with post hoc Tukey's HSD test	0.9997
3j	GAL4-only 1.0%	ATB-1>Kir2.1 0.25%	Kruskal-Wallis test with post hoc Tukey's HSD test	0.007
3j	GAL4-only 1.0%	ATB-1>Kir2.1 0.5%	Kruskal-Wallis test with post hoc Tukey's HSD test	0.0512
3j	GAL4-only 1.0%	ATB-1>Kir2.1 0.75%	Kruskal-Wallis test with post hoc Tukey's HSD test	1
3j	GAL4-only 1.0%	ATB-1>Kir2.1 1.0%	Kruskal-Wallis test with post hoc Tukey's HSD test	<0.0001
3j	GAL4-only 1.0%	ATB-1>Kir2.1 1.25%	Kruskal-Wallis test with post hoc Tukey's HSD test	<0.0001
3j	GAL4-only 1.0%	ATB-1>Kir2.1 1.5%	Kruskal-Wallis test with post hoc Tukey's HSD test	0.0023
3j	GAL4-only 1.0%	GAL4-only 1.5%	Kruskal-Wallis test with post hoc Tukey's HSD test	1
3j	GAL4-only 1.25%	UAS-only 0.25%	Kruskal-Wallis test with post hoc Tukey's HSD test	0.9714
3j	GAL4-only 1.25%	UAS-only 0.5%	Kruskal-Wallis test with post hoc Tukey's HSD test	1
3j	GAL4-only 1.25%	UAS-only 0.75%	Kruskal-Wallis test with post hoc Tukey's HSD test	1
3j	GAL4-only 1.25%	UAS-only 1.0%	Kruskal-Wallis test with post hoc Tukey's HSD test	0.9996
3j	GAL4-only 1.25%	UAS-only 1.25%	Kruskal-Wallis test with post hoc Tukey's HSD test	1
3j	GAL4-only 1.25%	UAS-only 1.5%	Kruskal-Wallis test with post hoc Tukey's HSD test	0.9997
3j	GAL4-only 1.25%	ATB-1>Kir2.1 0.25%	Kruskal-Wallis test with post hoc Tukey's HSD test	0.0826
3j	GAL4-only 1.25%	ATB-1>Kir2.1 0.5%	Kruskal-Wallis test with post hoc Tukey's HSD test	0.2212
3j	GAL4-only 1.25%	ATB-1>Kir2.1 0.75%	Kruskal-Wallis test with post hoc Tukey's HSD test	1
3j	GAL4-only 1.25%	ATB-1>Kir2.1 1.0%	Kruskal-Wallis test with post hoc Tukey's HSD test	0.0019
3j	GAL4-only 1.25%	ATB-1>Kir2.1 1.25%	Kruskal-Wallis test with post hoc Tukey's HSD test	0.0001
3j	GAL4-only 1.25%	ATB-1>Kir2.1 1.5%	Kruskal-Wallis test with post hoc Tukey's HSD test	0.0097
3j	GAL4-only 1.5%	UAS-only 0.25%	Kruskal-Wallis test with post hoc Tukey's HSD test	0.6242
3j	GAL4-only 1.5%	UAS-only 0.5%	Kruskal-Wallis test with post hoc Tukey's HSD test	0.9995
3j	GAL4-only 1.5%	UAS-only 0.75%	Kruskal-Wallis test with post hoc Tukey's HSD test	1
3j	GAL4-only 1.5%	UAS-only 1.0%	Kruskal-Wallis test with post hoc Tukey's HSD test	0.9086

3j	GAL4-only 1.5%	UAS-only 1.25%	Kruskal-Wallis test with post hoc Tukey's HSD test	1
3j	GAL4-only 1.5%	UAS-only 1.5%	Kruskal-Wallis test with post hoc Tukey's HSD test	0.919
3j	GAL4-only 1.5%	ATB-1>Kir2.1 0.25%	Kruskal-Wallis test with post hoc Tukey's HSD test	0.012
3j	GAL4-only 1.5%	ATB-1>Kir2.1 0.5%	Kruskal-Wallis test with post hoc Tukey's HSD test	0.0405
3j	GAL4-only 1.5%	ATB-1>Kir2.1 0.75%	Kruskal-Wallis test with post hoc Tukey's HSD test	0.9984
3j	GAL4-only 1.5%	ATB-1>Kir2.1 1.0%	Kruskal-Wallis test with post hoc Tukey's HSD test	0.0002
3j	GAL4-only 1.5%	ATB-1>Kir2.1 1.25%	Kruskal-Wallis test with post hoc Tukey's HSD test	<0.0001
3j	GAL4-only 1.5%	ATB-1>Kir2.1 1.5%	Kruskal-Wallis test with post hoc Tukey's HSD test	0.0014
3j	UAS-only 0.25%	UAS-only 0.5%	Kruskal-Wallis test with post hoc Tukey's HSD test	0.946
3j	UAS-only 0.25%	UAS-only 0.75%	Kruskal-Wallis test with post hoc Tukey's HSD test	0.8907
3j	UAS-only 0.25%	UAS-only 1.0%	Kruskal-Wallis test with post hoc Tukey's HSD test	1
3j	UAS-only 0.25%	UAS-only 1.25%	Kruskal-Wallis test with post hoc Tukey's HSD test	0.3341
3j	UAS-only 0.25%	UAS-only 1.5%	Kruskal-Wallis test with post hoc Tukey's HSD test	1
3j	UAS-only 0.25%	ATB-1>Kir2.1 0.25%	Kruskal-Wallis test with post hoc Tukey's HSD test	0.8582
3j	UAS-only 0.25%	ATB-1>Kir2.1 0.5%	Kruskal-Wallis test with post hoc Tukey's HSD test	0.9839
3j	UAS-only 0.25%	ATB-1>Kir2.1 0.75%	Kruskal-Wallis test with post hoc Tukey's HSD test	0.992
3j	UAS-only 0.25%	ATB-1>Kir2.1 1.0%	Kruskal-Wallis test with post hoc Tukey's HSD test	0.0775
3j	UAS-only 0.25%	ATB-1>Kir2.1 1.25%	Kruskal-Wallis test with post hoc Tukey's HSD test	0.0055
3j	UAS-only 0.25%	ATB-1>Kir2.1 1.5%	Kruskal-Wallis test with post hoc Tukey's HSD test	0.2085
3j	UAS-only 0.5%	UAS-only 0.75%	Kruskal-Wallis test with post hoc Tukey's HSD test	1
3j	UAS-only 0.5%	UAS-only 1.0%	Kruskal-Wallis test with post hoc Tukey's HSD test	0.9998
3j	UAS-only 0.5%	UAS-only 1.25%	Kruskal-Wallis test with post hoc Tukey's HSD test	0.9984
3j	UAS-only 0.5%	UAS-only 1.5%	Kruskal-Wallis test with post hoc Tukey's HSD test	0.9999
3j	UAS-only 0.5%	ATB-1>Kir2.1 0.25%	Kruskal-Wallis test with post hoc Tukey's HSD test	0.0044
3j	UAS-only 0.5%	ATB-1>Kir2.1 0.5%	Kruskal-Wallis test with post hoc Tukey's HSD test	0.041
3j	UAS-only 0.5%	ATB-1>Kir2.1 0.75%	Kruskal-Wallis test with post hoc Tukey's HSD test	1
3j	UAS-only 0.5%	ATB-1>Kir2.1 1.0%	Kruskal-Wallis test with post hoc Tukey's HSD test	<0.0001
3j	UAS-only 0.5%	ATB-1>Kir2.1 1.25%	Kruskal-Wallis test with post hoc Tukey's HSD test	<0.0001
3j	UAS-only 0.5%	ATB-1>Kir2.1 1.5%	Kruskal-Wallis test with post hoc Tukey's HSD test	0.002
3j	UAS-only 0.75%	UAS-only 1.0%	Kruskal-Wallis test with post hoc Tukey's HSD test	0.9979
3j	UAS-only 0.75%	UAS-only 1.25%	Kruskal-Wallis test with post hoc Tukey's HSD test	1
3j	UAS-only 0.75%	UAS-only 1.5%	Kruskal-Wallis test with post hoc Tukey's HSD test	0.9985
3j	UAS-only 0.75%	ATB-1>Kir2.1 0.25%	Kruskal-Wallis test with post hoc Tukey's HSD test	0.0051
3j	UAS-only 0.75%	ATB-1>Kir2.1 0.5%	Kruskal-Wallis test with post hoc Tukey's HSD test	0.0371
3j	UAS-only 0.75%	ATB-1>Kir2.1 0.75%	Kruskal-Wallis test with post hoc Tukey's HSD test	1
3j	UAS-only 0.75%	ATB-1>Kir2.1 1.0%	Kruskal-Wallis test with post hoc Tukey's HSD test	<0.0001
3j	UAS-only 0.75%	ATB-1>Kir2.1 1.25%	Kruskal-Wallis test with post hoc Tukey's HSD test	<0.0001
3j	UAS-only 0.75%	ATB-1>Kir2.1 1.5%	Kruskal-Wallis test with post hoc Tukey's HSD test	0.0016
3j	UAS-only 1.0%	UAS-only 1.25%	Kruskal-Wallis test with post hoc Tukey's HSD test	0.7574
3j	UAS-only 1.0%	UAS-only 1.5%	Kruskal-Wallis test with post hoc Tukey's HSD test	1
3j	UAS-only 1.0%	ATB-1>Kir2.1 0.25%	Kruskal-Wallis test with post hoc Tukey's HSD test	0.3038
3j	UAS-only 1.0%	ATB-1>Kir2.1 0.5%	Kruskal-Wallis test with post hoc Tukey's HSD test	0.6688
3j	UAS-only 1.0%	ATB-1>Kir2.1 0.75%	Kruskal-Wallis test with post hoc Tukey's HSD test	1
3j	UAS-only 1.0%	ATB-1>Kir2.1 1.0%	Kruskal-Wallis test with post hoc Tukey's HSD test	0.0056
3j	UAS-only 1.0%	ATB-1>Kir2.1 1.25%	Kruskal-Wallis test with post hoc Tukey's HSD test	0.0002
3j	UAS-only 1.0%	ATB-1>Kir2.1 1.5%	Kruskal-Wallis test with post hoc Tukey's HSD test	0.0484
3j	UAS-only 1.25%	UAS-only 1.5%	Kruskal-Wallis test with post hoc Tukey's HSD test	0.7888
3j	UAS-only 1.25%	ATB-1>Kir2.1 0.25%	Kruskal-Wallis test with post hoc Tukey's HSD test	0.0003
3j	UAS-only 1.25%	ATB-1>Kir2.1 0.5%	Kruskal-Wallis test with post hoc Tukey's HSD test	0.0027
3j	UAS-only 1.25%	ATB-1>Kir2.1 0.75%	Kruskal-Wallis test with post hoc Tukey's HSD test	0.9953
3j	UAS-only 1.25%	ATB-1>Kir2.1 1.0%	Kruskal-Wallis test with post hoc Tukey's HSD test	<0.0001
3j	UAS-only 1.25%	ATB-1>Kir2.1 1.25%	Kruskal-Wallis test with post hoc Tukey's HSD test	<0.0001
3j	UAS-only 1.25%	ATB-1>Kir2.1 1.5%	Kruskal-Wallis test with post hoc Tukey's HSD test	0.0001

3j	UAS-only 1.5%	ATB-1>Kir2.1 0.25%	Kruskal-Wallis test with post hoc Tukey's HSD test	0.359
3j	UAS-only 1.5%	ATB-1>Kir2.1 0.5%	Kruskal-Wallis test with post hoc Tukey's HSD test	0.7136
3j	UAS-only 1.5%	ATB-1>Kir2.1 0.75%	Kruskal-Wallis test with post hoc Tukey's HSD test	1
3j	UAS-only 1.5%	ATB-1>Kir2.1 1.0%	Kruskal-Wallis test with post hoc Tukey's HSD test	0.0085
3j	UAS-only 1.5%	ATB-1>Kir2.1 1.25%	Kruskal-Wallis test with post hoc Tukey's HSD test	0.0003
3j	UAS-only 1.5%	ATB-1>Kir2.1 1.5%	Kruskal-Wallis test with post hoc Tukey's HSD test	0.056
3j	ATB-1>Kir2.1 0.25%	ATB-1>Kir2.1 0.5%	Kruskal-Wallis test with post hoc Tukey's HSD test	1
3j	ATB-1>Kir2.1 0.25%	ATB-1>Kir2.1 0.75%	Kruskal-Wallis test with post hoc Tukey's HSD test	0.0322
3j	ATB-1>Kir2.1 0.25%	ATB-1>Kir2.1 1.0%	Kruskal-Wallis test with post hoc Tukey's HSD test	0.9761
3j	ATB-1>Kir2.1 0.25%	ATB-1>Kir2.1 1.25%	Kruskal-Wallis test with post hoc Tukey's HSD test	0.5526
3j	ATB-1>Kir2.1 0.25%	ATB-1>Kir2.1 1.5%	Kruskal-Wallis test with post hoc Tukey's HSD test	0.9633
3j	ATB-1>Kir2.1 0.5%	ATB-1>Kir2.1 0.75%	Kruskal-Wallis test with post hoc Tukey's HSD test	0.1534
3j	ATB-1>Kir2.1 0.5%	ATB-1>Kir2.1 1.0%	Kruskal-Wallis test with post hoc Tukey's HSD test	0.9118
3j	ATB-1>Kir2.1 0.5%	ATB-1>Kir2.1 1.25%	Kruskal-Wallis test with post hoc Tukey's HSD test	0.3901
3j	ATB-1>Kir2.1 0.5%	ATB-1>Kir2.1 1.5%	Kruskal-Wallis test with post hoc Tukey's HSD test	0.9055
3j	ATB-1>Kir2.1 0.75%	ATB-1>Kir2.1 1.0%	Kruskal-Wallis test with post hoc Tukey's HSD test	0.0002
3j	ATB-1>Kir2.1 0.75%	ATB-1>Kir2.1 1.25%	Kruskal-Wallis test with post hoc Tukey's HSD test	<0.0001
3j	ATB-1>Kir2.1 0.75%	ATB-1>Kir2.1 1.5%	Kruskal-Wallis test with post hoc Tukey's HSD test	0.0066
3j	ATB-1>Kir2.1 1.0%	ATB-1>Kir2.1 1.25%	Kruskal-Wallis test with post hoc Tukey's HSD test	1
3j	ATB-1>Kir2.1 1.0%	ATB-1>Kir2.1 1.5%	Kruskal-Wallis test with post hoc Tukey's HSD test	1
3j	ATB-1>Kir2.1 1.25%	ATB-1>Kir2.1 1.5%	Kruskal-Wallis test with post hoc Tukey's HSD test	1
3k	GAL4-only 0.25%	GAL4-only 0.5%	Kruskal-Wallis test with post hoc Tukey's HSD test	1
3k	GAL4-only 0.25%	GAL4-only 0.75%	Kruskal-Wallis test with post hoc Tukey's HSD test	0.9995
3k	GAL4-only 0.25%	GAL4-only 1.0%	Kruskal-Wallis test with post hoc Tukey's HSD test	1
3k	GAL4-only 0.25%	GAL4-only 1.25%	Kruskal-Wallis test with post hoc Tukey's HSD test	1
3k	GAL4-only 0.25%	GAL4-only 1.5%	Kruskal-Wallis test with post hoc Tukey's HSD test	1
3k	GAL4-only 0.25%	UAS-only 0.25%	Kruskal-Wallis test with post hoc Tukey's HSD test	1
3k	GAL4-only 0.25%	UAS-only 0.5%	Kruskal-Wallis test with post hoc Tukey's HSD test	1
3k	GAL4-only 0.25%	UAS-only 0.75%	Kruskal-Wallis test with post hoc Tukey's HSD test	0.0046
3k	GAL4-only 0.25%	UAS-only 1.0%	Kruskal-Wallis test with post hoc Tukey's HSD test	0.976
3k	GAL4-only 0.25%	UAS-only 1.25%	Kruskal-Wallis test with post hoc Tukey's HSD test	0.9998
3k	GAL4-only 0.25%	UAS-only 1.5%	Kruskal-Wallis test with post hoc Tukey's HSD test	<0.0001
3k	GAL4-only 0.25%	ATB-1>Kir2.1 0.25%	Kruskal-Wallis test with post hoc Tukey's HSD test	0.1706
3k	GAL4-only 0.25%	ATB-1>Kir2.1 0.5%	Kruskal-Wallis test with post hoc Tukey's HSD test	0.001
3k	GAL4-only 0.25%	ATB-1>Kir2.1 0.75%	Kruskal-Wallis test with post hoc Tukey's HSD test	<0.0001
3k	GAL4-only 0.25%	ATB-1>Kir2.1 1.0%	Kruskal-Wallis test with post hoc Tukey's HSD test	<0.0001
3k	GAL4-only 0.25%	ATB-1>Kir2.1 1.25%	Kruskal-Wallis test with post hoc Tukey's HSD test	<0.0001
3k	GAL4-only 0.25%	ATB-1>Kir2.1 1.5%	Kruskal-Wallis test with post hoc Tukey's HSD test	0.0126
3k	GAL4-only 0.5%	GAL4-only 0.75%	Kruskal-Wallis test with post hoc Tukey's HSD test	0.9999
3k	GAL4-only 0.5%	GAL4-only 1.0%	Kruskal-Wallis test with post hoc Tukey's HSD test	0.9991
3k	GAL4-only 0.5%	GAL4-only 1.25%	Kruskal-Wallis test with post hoc Tukey's HSD test	1
3k	GAL4-only 0.5%	GAL4-only 1.5%	Kruskal-Wallis test with post hoc Tukey's HSD test	1
3k	GAL4-only 0.5%	UAS-only 0.25%	Kruskal-Wallis test with post hoc Tukey's HSD test	1
3k	GAL4-only 0.5%	UAS-only 0.5%	Kruskal-Wallis test with post hoc Tukey's HSD test	1
3k	GAL4-only 0.5%	UAS-only 0.75%	Kruskal-Wallis test with post hoc Tukey's HSD test	0.0001
3k	GAL4-only 0.5%	UAS-only 1.0%	Kruskal-Wallis test with post hoc Tukey's HSD test	0.9681
3k	GAL4-only 0.5%	UAS-only 1.25%	Kruskal-Wallis test with post hoc Tukey's HSD test	1
3k	GAL4-only 0.5%	UAS-only 1.5%	Kruskal-Wallis test with post hoc Tukey's HSD test	<0.0001
3k	GAL4-only 0.5%	ATB-1>Kir2.1 0.25%	Kruskal-Wallis test with post hoc Tukey's HSD test	0.0249
3k	GAL4-only 0.5%	ATB-1>Kir2.1 0.5%	Kruskal-Wallis test with post hoc Tukey's HSD test	<0.0001
3k	GAL4-only 0.5%	ATB-1>Kir2.1 0.75%	Kruskal-Wallis test with post hoc Tukey's HSD test	<0.0001
3k	GAL4-only 0.5%	ATB-1>Kir2.1 1.0%	Kruskal-Wallis test with post hoc Tukey's HSD test	<0.0001
3k	GAL4-only 0.5%	ATB-1>Kir2.1 1.25%	Kruskal-Wallis test with post hoc Tukey's HSD test	<0.0001
3k	GAL4-only 0.5%	ATB-1>Kir2.1 1.5%	Kruskal-Wallis test with post hoc Tukey's HSD test	0.0074
3k	GAL4-only 0.75%	GAL4-only 1.0%	Kruskal-Wallis test with post hoc Tukey's HSD test	0.7938
3k	GAL4-only 0.75%	GAL4-only 1.25%	Kruskal-Wallis test with post hoc Tukey's HSD test	0.9812

3k	GAL4-only 0.75%	GAL4-only 1.5%	Kruskal-Wallis test with post hoc Tukey's HSD test	0.9987
3k	GAL4-only 0.75%	UAS-only 0.25%	Kruskal-Wallis test with post hoc Tukey's HSD test	0.986
3k	GAL4-only 0.75%	UAS-only 0.5%	Kruskal-Wallis test with post hoc Tukey's HSD test	0.9998
3k	GAL4-only 0.75%	UAS-only 0.75%	Kruskal-Wallis test with post hoc Tukey's HSD test	0.0401
3k	GAL4-only 0.75%	UAS-only 1.0%	Kruskal-Wallis test with post hoc Tukey's HSD test	1
3k	GAL4-only 0.75%	UAS-only 1.25%	Kruskal-Wallis test with post hoc Tukey's HSD test	1
3k	GAL4-only 0.75%	UAS-only 1.5%	Kruskal-Wallis test with post hoc Tukey's HSD test	<0.0001
3k	GAL4-only 0.75%	ATB-1>Kir2.1 0.25%	Kruskal-Wallis test with post hoc Tukey's HSD test	0.7277
3k	GAL4-only 0.75%	ATB-1>Kir2.1 0.5%	Kruskal-Wallis test with post hoc Tukey's HSD test	0.008
3k	GAL4-only 0.75%	ATB-1>Kir2.1 0.75%	Kruskal-Wallis test with post hoc Tukey's HSD test	<0.0001
3k	GAL4-only 0.75%	ATB-1>Kir2.1 1.0%	Kruskal-Wallis test with post hoc Tukey's HSD test	0.0003
3k	GAL4-only 0.75%	ATB-1>Kir2.1 1.25%	Kruskal-Wallis test with post hoc Tukey's HSD test	0.0002
3k	GAL4-only 0.75%	ATB-1>Kir2.1 1.5%	Kruskal-Wallis test with post hoc Tukey's HSD test	0.0814
3k	GAL4-only 1.0%	GAL4-only 1.25%	Kruskal-Wallis test with post hoc Tukey's HSD test	1
3k	GAL4-only 1.0%	GAL4-only 1.5%	Kruskal-Wallis test with post hoc Tukey's HSD test	1
3k	GAL4-only 1.0%	UAS-only 0.25%	Kruskal-Wallis test with post hoc Tukey's HSD test	1
3k	GAL4-only 1.0%	UAS-only 0.5%	Kruskal-Wallis test with post hoc Tukey's HSD test	0.9988
3k	GAL4-only 1.0%	UAS-only 0.75%	Kruskal-Wallis test with post hoc Tukey's HSD test	<0.0001
3k	GAL4-only 1.0%	UAS-only 1.0%	Kruskal-Wallis test with post hoc Tukey's HSD test	0.2773
3k	GAL4-only 1.0%	UAS-only 1.25%	Kruskal-Wallis test with post hoc Tukey's HSD test	0.8708
3k	GAL4-only 1.0%	UAS-only 1.5%	Kruskal-Wallis test with post hoc Tukey's HSD test	<0.0001
3k	GAL4-only 1.0%	ATB-1>Kir2.1 0.25%	Kruskal-Wallis test with post hoc Tukey's HSD test	0.0002
3k	GAL4-only 1.0%	ATB-1>Kir2.1 0.5%	Kruskal-Wallis test with post hoc Tukey's HSD test	<0.0001
3k	GAL4-only 1.0%	ATB-1>Kir2.1 0.75%	Kruskal-Wallis test with post hoc Tukey's HSD test	<0.0001
3k	GAL4-only 1.0%	ATB-1>Kir2.1 1.0%	Kruskal-Wallis test with post hoc Tukey's HSD test	<0.0001
3k	GAL4-only 1.0%	ATB-1>Kir2.1 1.25%	Kruskal-Wallis test with post hoc Tukey's HSD test	<0.0001
3k	GAL4-only 1.0%	ATB-1>Kir2.1 1.5%	Kruskal-Wallis test with post hoc Tukey's HSD test	0.0006
3k	GAL4-only 1.0%	GAL4-only 1.5%	Kruskal-Wallis test with post hoc Tukey's HSD test	1
3k	GAL4-only 1.25%	UAS-only 0.25%	Kruskal-Wallis test with post hoc Tukey's HSD test	1
3k	GAL4-only 1.25%	UAS-only 0.5%	Kruskal-Wallis test with post hoc Tukey's HSD test	1
3k	GAL4-only 1.25%	UAS-only 0.75%	Kruskal-Wallis test with post hoc Tukey's HSD test	0.0002
3k	GAL4-only 1.25%	UAS-only 1.0%	Kruskal-Wallis test with post hoc Tukey's HSD test	0.7949
3k	GAL4-only 1.25%	UAS-only 1.25%	Kruskal-Wallis test with post hoc Tukey's HSD test	0.9909
3k	GAL4-only 1.25%	UAS-only 1.5%	Kruskal-Wallis test with post hoc Tukey's HSD test	<0.0001
3k	GAL4-only 1.25%	ATB-1>Kir2.1 0.25%	Kruskal-Wallis test with post hoc Tukey's HSD test	0.0235
3k	GAL4-only 1.25%	ATB-1>Kir2.1 0.5%	Kruskal-Wallis test with post hoc Tukey's HSD test	<0.0001
3k	GAL4-only 1.25%	ATB-1>Kir2.1 0.75%	Kruskal-Wallis test with post hoc Tukey's HSD test	<0.0001
3k	GAL4-only 1.25%	ATB-1>Kir2.1 1.0%	Kruskal-Wallis test with post hoc Tukey's HSD test	<0.0001
3k	GAL4-only 1.25%	ATB-1>Kir2.1 1.25%	Kruskal-Wallis test with post hoc Tukey's HSD test	<0.0001
3k	GAL4-only 1.25%	ATB-1>Kir2.1 1.5%	Kruskal-Wallis test with post hoc Tukey's HSD test	0.0033
3k	GAL4-only 1.5%	UAS-only 0.25%	Kruskal-Wallis test with post hoc Tukey's HSD test	1
3k	GAL4-only 1.5%	UAS-only 0.5%	Kruskal-Wallis test with post hoc Tukey's HSD test	1
3k	GAL4-only 1.5%	UAS-only 0.75%	Kruskal-Wallis test with post hoc Tukey's HSD test	0.0032
3k	GAL4-only 1.5%	UAS-only 1.0%	Kruskal-Wallis test with post hoc Tukey's HSD test	0.9605
3k	GAL4-only 1.5%	UAS-only 1.25%	Kruskal-Wallis test with post hoc Tukey's HSD test	0.9995
3k	GAL4-only 1.5%	UAS-only 1.5%	Kruskal-Wallis test with post hoc Tukey's HSD test	<0.0001
3k	GAL4-only 1.5%	ATB-1>Kir2.1 0.25%	Kruskal-Wallis test with post hoc Tukey's HSD test	0.1351
3k	GAL4-only 1.5%	ATB-1>Kir2.1 0.5%	Kruskal-Wallis test with post hoc Tukey's HSD test	0.0007
3k	GAL4-only 1.5%	ATB-1>Kir2.1 0.75%	Kruskal-Wallis test with post hoc Tukey's HSD test	<0.0001
3k	GAL4-only 1.5%	ATB-1>Kir2.1 1.0%	Kruskal-Wallis test with post hoc Tukey's HSD test	<0.0001
3k	GAL4-only 1.5%	ATB-1>Kir2.1 1.25%	Kruskal-Wallis test with post hoc Tukey's HSD test	<0.0001
3k	GAL4-only 1.5%	ATB-1>Kir2.1 1.5%	Kruskal-Wallis test with post hoc Tukey's HSD test	0.0102
3k	UAS-only 0.25%	UAS-only 0.5%	Kruskal-Wallis test with post hoc Tukey's HSD test	1
3k	UAS-only 0.25%	UAS-only 0.75%	Kruskal-Wallis test with post hoc Tukey's HSD test	<0.0001
3k	UAS-only 0.25%	UAS-only 1.0%	Kruskal-Wallis test with post hoc Tukey's HSD test	0.7644
3k	UAS-only 0.25%	UAS-only 1.25%	Kruskal-Wallis test with post hoc Tukey's HSD test	0.9943
3k	UAS-only 0.25%	UAS-only 1.5%	Kruskal-Wallis test with post hoc Tukey's HSD test	<0.0001
3k	UAS-only 0.25%	ATB-1>Kir2.1 0.25%	Kruskal-Wallis test with post hoc Tukey's HSD test	0.0071

3k	UAS-only 0.25%	ATB-1>Kir2.1 0.5%	Kruskal-Wallis test with post hoc Tukey's HSD test	<0.0001
3k	UAS-only 0.25%	ATB-1>Kir2.1 0.75%	Kruskal-Wallis test with post hoc Tukey's HSD test	<0.0001
3k	UAS-only 0.25%	ATB-1>Kir2.1 1.0%	Kruskal-Wallis test with post hoc Tukey's HSD test	<0.0001
3k	UAS-only 0.25%	ATB-1>Kir2.1 1.25%	Kruskal-Wallis test with post hoc Tukey's HSD test	<0.0001
3k	UAS-only 0.25%	ATB-1>Kir2.1 1.5%	Kruskal-Wallis test with post hoc Tukey's HSD test	0.0027
3k	UAS-only 0.5%	UAS-only 0.75%	Kruskal-Wallis test with post hoc Tukey's HSD test	<0.0001
3k	UAS-only 0.5%	UAS-only 1.0%	Kruskal-Wallis test with post hoc Tukey's HSD test	0.9508
3k	UAS-only 0.5%	UAS-only 1.25%	Kruskal-Wallis test with post hoc Tukey's HSD test	1
3k	UAS-only 0.5%	UAS-only 1.5%	Kruskal-Wallis test with post hoc Tukey's HSD test	<0.0001
3k	UAS-only 0.5%	ATB-1>Kir2.1 0.25%	Kruskal-Wallis test with post hoc Tukey's HSD test	0.0137
3k	UAS-only 0.5%	ATB-1>Kir2.1 0.5%	Kruskal-Wallis test with post hoc Tukey's HSD test	<0.0001
3k	UAS-only 0.5%	ATB-1>Kir2.1 0.75%	Kruskal-Wallis test with post hoc Tukey's HSD test	<0.0001
3k	UAS-only 0.5%	ATB-1>Kir2.1 1.0%	Kruskal-Wallis test with post hoc Tukey's HSD test	<0.0001
3k	UAS-only 0.5%	ATB-1>Kir2.1 1.25%	Kruskal-Wallis test with post hoc Tukey's HSD test	<0.0001
3k	UAS-only 0.5%	ATB-1>Kir2.1 1.5%	Kruskal-Wallis test with post hoc Tukey's HSD test	0.0062
3k	UAS-only 0.75%	UAS-only 1.0%	Kruskal-Wallis test with post hoc Tukey's HSD test	0.0927
3k	UAS-only 0.75%	UAS-only 1.25%	Kruskal-Wallis test with post hoc Tukey's HSD test	0.0339
3k	UAS-only 0.75%	UAS-only 1.5%	Kruskal-Wallis test with post hoc Tukey's HSD test	0.956
3k	UAS-only 0.75%	ATB-1>Kir2.1 0.25%	Kruskal-Wallis test with post hoc Tukey's HSD test	0.9815
3k	UAS-only 0.75%	ATB-1>Kir2.1 0.5%	Kruskal-Wallis test with post hoc Tukey's HSD test	1
3k	UAS-only 0.75%	ATB-1>Kir2.1 0.75%	Kruskal-Wallis test with post hoc Tukey's HSD test	0.5613
3k	UAS-only 0.75%	ATB-1>Kir2.1 1.0%	Kruskal-Wallis test with post hoc Tukey's HSD test	0.9962
3k	UAS-only 0.75%	ATB-1>Kir2.1 1.25%	Kruskal-Wallis test with post hoc Tukey's HSD test	0.91
3k	UAS-only 0.75%	ATB-1>Kir2.1 1.5%	Kruskal-Wallis test with post hoc Tukey's HSD test	0.9978
3k	UAS-only 1.0%	UAS-only 1.25%	Kruskal-Wallis test with post hoc Tukey's HSD test	1
3k	UAS-only 1.0%	UAS-only 1.5%	Kruskal-Wallis test with post hoc Tukey's HSD test	<0.0001
3k	UAS-only 1.0%	ATB-1>Kir2.1 0.25%	Kruskal-Wallis test with post hoc Tukey's HSD test	0.9259
3k	UAS-only 1.0%	ATB-1>Kir2.1 0.5%	Kruskal-Wallis test with post hoc Tukey's HSD test	0.0196
3k	UAS-only 1.0%	ATB-1>Kir2.1 0.75%	Kruskal-Wallis test with post hoc Tukey's HSD test	<0.0001
3k	UAS-only 1.0%	ATB-1>Kir2.1 1.0%	Kruskal-Wallis test with post hoc Tukey's HSD test	0.0006
3k	UAS-only 1.0%	ATB-1>Kir2.1 1.25%	Kruskal-Wallis test with post hoc Tukey's HSD test	0.0004
3k	UAS-only 1.0%	ATB-1>Kir2.1 1.5%	Kruskal-Wallis test with post hoc Tukey's HSD test	0.1476
3k	UAS-only 1.25%	UAS-only 1.5%	Kruskal-Wallis test with post hoc Tukey's HSD test	<0.0001
3k	UAS-only 1.25%	ATB-1>Kir2.1 0.25%	Kruskal-Wallis test with post hoc Tukey's HSD test	0.6765
3k	UAS-only 1.25%	ATB-1>Kir2.1 0.5%	Kruskal-Wallis test with post hoc Tukey's HSD test	0.0067
3k	UAS-only 1.25%	ATB-1>Kir2.1 0.75%	Kruskal-Wallis test with post hoc Tukey's HSD test	<0.0001
3k	UAS-only 1.25%	ATB-1>Kir2.1 1.0%	Kruskal-Wallis test with post hoc Tukey's HSD test	0.0002
3k	UAS-only 1.25%	ATB-1>Kir2.1 1.25%	Kruskal-Wallis test with post hoc Tukey's HSD test	0.0001
3k	UAS-only 1.25%	ATB-1>Kir2.1 1.5%	Kruskal-Wallis test with post hoc Tukey's HSD test	0.0716
3k	UAS-only 1.5%	ATB-1>Kir2.1 0.25%	Kruskal-Wallis test with post hoc Tukey's HSD test	0.0456
3k	UAS-only 1.5%	ATB-1>Kir2.1 0.5%	Kruskal-Wallis test with post hoc Tukey's HSD test	0.9958
3k	UAS-only 1.5%	ATB-1>Kir2.1 0.75%	Kruskal-Wallis test with post hoc Tukey's HSD test	1
3k	UAS-only 1.5%	ATB-1>Kir2.1 1.0%	Kruskal-Wallis test with post hoc Tukey's HSD test	1
3k	UAS-only 1.5%	ATB-1>Kir2.1 1.25%	Kruskal-Wallis test with post hoc Tukey's HSD test	1
3k	UAS-only 1.5%	ATB-1>Kir2.1 1.5%	Kruskal-Wallis test with post hoc Tukey's HSD test	1
3k	ATB-1>Kir2.1 0.25%	ATB-1>Kir2.1 0.5%	Kruskal-Wallis test with post hoc Tukey's HSD test	0.8418
3k	ATB-1>Kir2.1 0.25%	ATB-1>Kir2.1 0.75%	Kruskal-Wallis test with post hoc Tukey's HSD test	0.0023
3k	ATB-1>Kir2.1 0.25%	ATB-1>Kir2.1 1.0%	Kruskal-Wallis test with post hoc Tukey's HSD test	0.197
3k	ATB-1>Kir2.1 0.25%	ATB-1>Kir2.1 1.25%	Kruskal-Wallis test with post hoc Tukey's HSD test	0.0861
3k	ATB-1>Kir2.1 0.25%	ATB-1>Kir2.1 1.5%	Kruskal-Wallis test with post hoc Tukey's HSD test	0.7555
3k	ATB-1>Kir2.1 0.5%	ATB-1>Kir2.1 0.75%	Kruskal-Wallis test with post hoc Tukey's HSD test	0.8066

3k	ATB-1>Kir2.1 0.5%	ATB-1>Kir2.1 1.0%	Kruskal-Wallis test with post hoc Tukey's HSD test	0.9999
3k	ATB-1>Kir2.1 0.5%	ATB-1>Kir2.1 1.25%	Kruskal-Wallis test with post hoc Tukey's HSD test	0.9803
3k	ATB-1>Kir2.1 0.5%	ATB-1>Kir2.1 1.5%	Kruskal-Wallis test with post hoc Tukey's HSD test	0.9997
3k	ATB-1>Kir2.1 0.75%	ATB-1>Kir2.1 1.0%	Kruskal-Wallis test with post hoc Tukey's HSD test	1
3k	ATB-1>Kir2.1 0.75%	ATB-1>Kir2.1 1.25%	Kruskal-Wallis test with post hoc Tukey's HSD test	1
3k	ATB-1>Kir2.1 0.75%	ATB-1>Kir2.1 1.5%	Kruskal-Wallis test with post hoc Tukey's HSD test	<0.0001
3k	ATB-1>Kir2.1 1.0%	ATB-1>Kir2.1 1.25%	Kruskal-Wallis test with post hoc Tukey's HSD test	1
3k	ATB-1>Kir2.1 1.0%	ATB-1>Kir2.1 1.5%	Kruskal-Wallis test with post hoc Tukey's HSD test	1
3k	ATB-1>Kir2.1 1.25%	ATB-1>Kir2.1 1.5%	Kruskal-Wallis test with post hoc Tukey's HSD test	1
4c	abort, 0.75% agarose	abort, 1.0% agarose	Kruskal-Wallis test with post hoc Tukey's HSD test	0.9516
4c	abort, 0.75% agarose	abort, 1.25% agarose	Kruskal-Wallis test with post hoc Tukey's HSD test	0.9997
4c	abort, 0.75% agarose	abort, 1.5% agarose	Kruskal-Wallis test with post hoc Tukey's HSD test	0.2716
4c	abort, 0.75% agarose	abort, 1.75% agarose	Kruskal-Wallis test with post hoc Tukey's HSD test	0.1482
4c	abort, 1.0% agarose	abort, 1.25% agarose	Kruskal-Wallis test with post hoc Tukey's HSD test	0.9728
4c	abort, 1.0% agarose	abort, 1.5% agarose	Kruskal-Wallis test with post hoc Tukey's HSD test	0.0141
4c	abort, 1.0% agarose	abort, 1.75% agarose	Kruskal-Wallis test with post hoc Tukey's HSD test	0.0035
4c	abort, 1.25% agarose	abort, 1.5% agarose	Kruskal-Wallis test with post hoc Tukey's HSD test	0.0674
4c	abort, 1.25% agarose	abort, 1.75% agarose	Kruskal-Wallis test with post hoc Tukey's HSD test	0.0213
4c	abort, 1.5% agarose	abort, 1.75% agarose	Kruskal-Wallis test with post hoc Tukey's HSD test	0.9979
4d	expel, 0.75% agarose	expel, 1.0% agarose	Kruskal-Wallis test with post hoc Tukey's HSD test	0.2492
4d	expel, 0.75% agarose	expel, 1.25% agarose	Kruskal-Wallis test with post hoc Tukey's HSD test	0.1088
4d	expel, 0.75% agarose	expel, 1.5% agarose	Kruskal-Wallis test with post hoc Tukey's HSD test	0.0000
4d	expel, 0.75% agarose	expel, 1.75% agarose	Kruskal-Wallis test with post hoc Tukey's HSD test	0.0000
4d	expel, 1.0% agarose	expel, 1.25% agarose	Kruskal-Wallis test with post hoc Tukey's HSD test	0.9997
4d	expel, 1.0% agarose	expel, 1.5% agarose	Kruskal-Wallis test with post hoc Tukey's HSD test	0.0156
4d	expel, 1.0% agarose	expel, 1.75% agarose	Kruskal-Wallis test with post hoc Tukey's HSD test	0.0070
4d	expel, 1.25% agarose	expel, 1.5% agarose	Kruskal-Wallis test with post hoc Tukey's HSD test	0.0140
4d	expel, 1.25% agarose	expel, 1.75% agarose	Kruskal-Wallis test with post hoc Tukey's HSD test	0.0059
4d	expel, 1.5% agarose	expel, 1.75% agarose	Kruskal-Wallis test with post hoc Tukey's HSD test	0.9994
5i	Incomplete expulsion	Pre-expulsion baseline	two-sided Wilcoxon signed rank test	3.47×10^{-6}
5i	Complete expulsion	Pre-expulsion baseline	two-sided Wilcoxon signed rank test	1.80×10^{-6}
5i	Post-expulsion	Pre-expulsion baseline	two-sided Wilcoxon signed rank test	0.7156
5l	Incomplete expulsion	Pre-expulsion baseline	two-sided Wilcoxon signed rank test	2.44×10^{-4}
5l	Ovipositor extrusion	Pre-expulsion baseline	two-sided Wilcoxon signed rank test	0.8394
6a	GAL4-only	PU-1>Kir2.1	two-sided Wilcoxon rank sum test	2.29×10^{-5}
6a	UAS-only	PU-1>Kir2.1	two-sided Wilcoxon rank sum test	2.33×10^{-6}
6b	GAL4-only	PU-1>Kir2.1	two-sided Wilcoxon rank sum test	3.33×10^{-7}
6b	UAS-only	PU-1>Kir2.1	two-sided Wilcoxon rank sum test	1.47×10^{-6}
6d	GAL4-only	PU-1>Kir2.1	two-sided Wilcoxon rank sum test	1.86×10^{-5}
6d	UAS-only	PU-1>Kir2.1	two-sided Wilcoxon rank sum test	1.68×10^{-8}
6e	GAL4-only	PU-1>Kir2.1	two-sided Wilcoxon rank sum test	1.13×10^{-4}
6e	UAS-only	PU-1>Kir2.1	two-sided Wilcoxon rank sum test	8.72×10^{-6}
6f	GAL4-only	PU-1>Kir2.1	two-sided Wilcoxon rank sum test	0.0033
6f	UAS-only	PU-1>Kir2.1	two-sided Wilcoxon rank sum test	0.0040
6g	GAL4-only	PU-1>Kir2.1	two-sided Wilcoxon rank sum test	8.66×10^{-4}
6g	UAS-only	PU-1>Kir2.1	two-sided Wilcoxon rank sum test	0.0063
8d	CMU-3>CsChR 0.1s	CMU-3-control 0.1s	two-sided Fisher's exact test	1
8d	CMU-3>CsChR 0.2s	CMU-3-control 0.2s	two-sided Fisher's exact test	<0.0001
8d	CMU-3>CsChR 0.5s	CMU-3-control 0.5s	two-sided Fisher's exact test	<0.0001
8d	CMU-3>CsChR 1.0s	CMU-3-control 1.0s	two-sided Fisher's exact test	<0.0001
8d	CMU-3>CsChR 0.1s	UAS-control 0.1s	two-sided Fisher's exact test	1
8d	CMU-3>CsChR 0.2s	UAS-control 0.2s	two-sided Fisher's exact test	0.0020
8d	CMU-3>CsChR 0.5s	UAS-control 0.5s	two-sided Fisher's exact test	<0.0001
8d	CMU-3>CsChR 1.0s	UAS-control 1.0s	two-sided Fisher's exact test	<0.0001
8d	CMU-4>CsChR 0.1s	CMU-4-control 0.1s	two-sided Fisher's exact test	1
8d	CMU-4>CsChR 0.2s	CMU-4-control 0.2s	two-sided Fisher's exact test	<0.0001
8d	CMU-4>CsChR 0.5s	CMU-4-control 0.5s	two-sided Fisher's exact test	<0.0001
8d	CMU-4>CsChR 1.0s	CMU-4-control 1.0s	two-sided Fisher's exact test	<0.0001
8d	CMU-4>CsChR 0.1s	UAS-control 0.1s	two-sided Fisher's exact test	1
8d	CMU-4>CsChR 0.2s	UAS-control 0.2s	two-sided Fisher's exact test	<0.0001
8d	CMU-4>CsChR 0.5s	UAS-control 0.5s	two-sided Fisher's exact test	<0.0001
8d	CMU-4>CsChR 1.0s	UAS-control 1.0s	two-sided Fisher's exact test	<0.0001
8g	GAL4-only	CMU-2>GtACR1	two-sided Wilcoxon rank sum test	8.60×10^{-4}

8g	UAS-only	CMU-2>GtACR1	two-sided Wilcoxon rank sum test	0.0101
8h	GAL4-only	CMU-2>GtACR1	two-sided Wilcoxon rank sum test	0.0017
8h	UAS-only	CMU-2>GtACR1	two-sided Wilcoxon rank sum test	0.0016
8j	GAL4-only	CMU-2>GtACR1	two-sided Wilcoxon rank sum test	0.8478
8j	UAS-only	CMU-2>GtACR1	two-sided Wilcoxon rank sum test	0.5281
8k	GAL4-only	CMU-2>GtACR1	two-sided Wilcoxon rank sum test	1.92x10^{-4}
8k	UAS-only	CMU-2>GtACR1	two-sided Wilcoxon rank sum test	4.33x10^{-5}
Extended Data Fig. 1a	Human1:Human2 background	Human1:DeepEtho background	two-sided Wilcoxon rank sum test	1.75x10^{-5}
Extended Data Fig. 1a	Human1:Human2 PE	Human1:DeepEtho PE	two-sided Wilcoxon rank sum test	7.04x10^{-10}
Extended Data Fig. 1a	Human1:Human2 bend	Human1:DeepEtho bend	two-sided Wilcoxon rank sum test	1.61x10^{-6}
Extended Data Fig. 1a	Human1:Human2 burrow	Human1:DeepEtho burrow	two-sided Wilcoxon rank sum test	3.96x10^{-8}
Extended Data Fig. 1a	Human1:Human2 egg	Human1:DeepEtho egg	two-sided Wilcoxon rank sum test	0.7111
Extended Data Fig. 1a	Human1:Human2 detach	Human1:DeepEtho PE	two-sided Wilcoxon rank sum test	1.61x10^{-9}
Extended Data Fig. 1a	Human1:Human2 groom	Human1:DeepEtho groom	two-sided Wilcoxon rank sum test	1.53x10^{-5}
Extended Data Fig. 1a	Human1:Human2 all	Human1:DeepEtho all	two-sided Wilcoxon rank sum test	2.44x10^{-9}
Extended Data Fig. 2f	Cluster #0, fraction in ± 60 s of “egg out”	Cluster #0, fraction in t-SNE embedding	one-sided Fisher’s exact test	1
Extended Data Fig. 2f	Cluster #1, fraction in ± 60 s of “egg out”	Cluster #1, fraction in t-SNE embedding	one-sided Fisher’s exact test	1
Extended Data Fig. 2f	Cluster #2, fraction in ± 60 s of “egg out”	Cluster #2, fraction in t-SNE embedding	one-sided Fisher’s exact test	0.1933
Extended Data Fig. 2f	Cluster #3, fraction in ± 60 s of “egg out”	Cluster #3, fraction in t-SNE embedding	one-sided Fisher’s exact test	0.4019
Extended Data Fig. 2f	Cluster #4, fraction in ± 60 s of “egg out”	Cluster #4, fraction in t-SNE embedding	one-sided Fisher’s exact test	<0.0001
Extended Data Fig. 2f	Cluster #5, fraction in ± 60 s of “egg out”	Cluster #5, fraction in t-SNE embedding	one-sided Fisher’s exact test	<0.0001
Extended Data Fig. 2f	Cluster #6, fraction in ± 60 s of “egg out”	Cluster #6, fraction in t-SNE embedding	one-sided Fisher’s exact test	<0.0001
Extended Data Fig. 2f	Cluster #7, fraction in ± 60 s of “egg out”	Cluster #7, fraction in t-SNE embedding	one-sided Fisher’s exact test	0.984
Extended Data Fig. 2f	Cluster #8, fraction in ± 60 s of “egg out”	Cluster #8, fraction in t-SNE embedding	one-sided Fisher’s exact test	<0.0001
Extended Data Fig. 2f	Cluster #9, fraction in ± 60 s of “egg out”	Cluster #9, fraction in t-SNE embedding	one-sided Fisher’s exact test	1
Extended Data Fig. 2f	Cluster #10, fraction in ± 60 s of “egg out”	Cluster #10, fraction in t-SNE embedding	one-sided Fisher’s exact test	1
Extended Data Fig. 2f	Cluster #11, fraction in ± 60 s of “egg out”	Cluster #11, fraction in t-SNE embedding	one-sided Fisher’s exact test	<0.0001
Extended Data Fig. 2f	Cluster #12, fraction in ± 60 s of “egg out”	Cluster #12, fraction in t-SNE embedding	one-sided Fisher’s exact test	<0.0001
Extended Data Fig. 2f	Cluster #13, fraction in ± 60 s of “egg out”	Cluster #13, fraction in t-SNE embedding	one-sided Fisher’s exact test	1
Extended Data Fig. 2f	Cluster #14, fraction in ± 60 s of “egg out”	Cluster #14, fraction in t-SNE embedding	one-sided Fisher’s exact test	<0.0001
Extended Data Fig. 2f	Cluster #15, fraction in ± 60 s of “egg out”	Cluster #15, fraction in t-SNE embedding	one-sided Fisher’s exact test	<0.0001
Extended Data Fig. 2f	Cluster #16, fraction in ± 60 s of “egg out”	Cluster #16, fraction in t-SNE embedding	one-sided Fisher’s exact test	1
Extended Data Fig. 2f	Cluster #17, fraction in ± 60 s of “egg out”	Cluster #17, fraction in t-SNE embedding	one-sided Fisher’s exact test	<0.0001
Extended Data Fig. 2f	Cluster #18, fraction in ± 60 s of “egg out”	Cluster #18, fraction in t-SNE embedding	one-sided Fisher’s exact test	<0.0001
Extended Data Fig. 2f	Cluster #19, fraction in ± 60 s of “egg out”	Cluster #19, fraction in t-SNE embedding	one-sided Fisher’s exact test	<0.0001
Extended Data Fig. 2f	Cluster #20, fraction in ± 60 s of “egg out”	Cluster #20, fraction in t-SNE embedding	one-sided Fisher’s exact test	<0.0001
Extended Data Fig. 2f	Cluster #21, fraction in ± 60 s of “egg out”	Cluster #21, fraction in t-SNE embedding	one-sided Fisher’s exact test	<0.0001
Extended Data Fig. 2f	Cluster #22, fraction in ± 60 s of “egg out”	Cluster #22, fraction in t-SNE embedding	one-sided Fisher’s exact test	1
Extended Data Fig. 2f	Cluster #23, fraction in ± 60 s of “egg out”	Cluster #23, fraction in t-SNE embedding	one-sided Fisher’s exact test	1

Extended Data Fig. 2f	Cluster #24, fraction in ± 60 s of "egg out"	Cluster #24, fraction in t-SNE embedding	one-sided Fisher's exact test	1
Extended Data Fig. 2f	Cluster #25, fraction in ± 60 s of "egg out"	Cluster #25, fraction in t-SNE embedding	one-sided Fisher's exact test	<0.0001
Extended Data Fig. 2f	Cluster #26, fraction in ± 60 s of "egg out"	Cluster #26, fraction in t-SNE embedding	one-sided Fisher's exact test	1
Extended Data Fig. 2f	Cluster #27, fraction in ± 60 s of "egg out"	Cluster #27, fraction in t-SNE embedding	one-sided Fisher's exact test	<0.0001
Extended Data Fig. 2f	Cluster #28, fraction in ± 60 s of "egg out"	Cluster #28, fraction in t-SNE embedding	one-sided Fisher's exact test	1
Extended Data Fig. 2f	Cluster #29, fraction in ± 60 s of "egg out"	Cluster #29, fraction in t-SNE embedding	one-sided Fisher's exact test	<0.0001
Extended Data Fig. 2f	Cluster #30, fraction in ± 60 s of "egg out"	Cluster #30, fraction in t-SNE embedding	one-sided Fisher's exact test	1
Extended Data Fig. 2f	Cluster #31, fraction in ± 60 s of "egg out"	Cluster #31, fraction in t-SNE embedding	one-sided Fisher's exact test	1
Extended Data Fig. 2f	Cluster #32, fraction in ± 60 s of "egg out"	Cluster #32, fraction in t-SNE embedding	one-sided Fisher's exact test	1
Extended Data Fig. 2f	Cluster #33, fraction in ± 60 s of "egg out"	Cluster #33, fraction in t-SNE embedding	one-sided Fisher's exact test	<0.0001
Extended Data Fig. 2f	Cluster #34, fraction in ± 60 s of "egg out"	Cluster #34, fraction in t-SNE embedding	one-sided Fisher's exact test	<0.0001
Extended Data Fig. 2f	Cluster #35, fraction in ± 60 s of "egg out"	Cluster #35, fraction in t-SNE embedding	one-sided Fisher's exact test	1
Extended Data Fig. 2f	Cluster #36, fraction in ± 60 s of "egg out"	Cluster #36, fraction in t-SNE embedding	one-sided Fisher's exact test	1
Extended Data Fig. 2f	Cluster #37, fraction in ± 60 s of "egg out"	Cluster #37, fraction in t-SNE embedding	one-sided Fisher's exact test	1
Extended Data Fig. 2f	Cluster #38, fraction in ± 60 s of "egg out"	Cluster #38, fraction in t-SNE embedding	one-sided Fisher's exact test	<0.0001
Extended Data Fig. 2f	Cluster #39, fraction in ± 60 s of "egg out"	Cluster #39, fraction in t-SNE embedding	one-sided Fisher's exact test	1
Extended Data Fig. 2f	Cluster #40, fraction in ± 60 s of "egg out"	Cluster #40, fraction in t-SNE embedding	one-sided Fisher's exact test	1
Extended Data Fig. 2f	Cluster #41, fraction in ± 60 s of "egg out"	Cluster #41, fraction in t-SNE embedding	one-sided Fisher's exact test	1
Extended Data Fig. 2f	Cluster #42, fraction in ± 60 s of "egg out"	Cluster #42, fraction in t-SNE embedding	one-sided Fisher's exact test	0.0003
Extended Data Fig. 2f	Cluster #43, fraction in ± 60 s of "egg out"	Cluster #43, fraction in t-SNE embedding	one-sided Fisher's exact test	1
Extended Data Fig. 2f	Cluster #44, fraction in ± 60 s of "egg out"	Cluster #44, fraction in t-SNE embedding	one-sided Fisher's exact test	0.4886
Extended Data Fig. 2f	Cluster #45, fraction in ± 60 s of "egg out"	Cluster #45, fraction in t-SNE embedding	one-sided Fisher's exact test	1
Extended Data Fig. 2f	Cluster #46, fraction in ± 60 s of "egg out"	Cluster #46, fraction in t-SNE embedding	one-sided Fisher's exact test	1
Extended Data Fig. 2f	Cluster #47, fraction in ± 60 s of "egg out"	Cluster #47, fraction in t-SNE embedding	one-sided Fisher's exact test	0.9834
Extended Data Fig. 2f	Cluster #48, fraction in ± 60 s of "egg out"	Cluster #48, fraction in t-SNE embedding	one-sided Fisher's exact test	<0.0001
Extended Data Fig. 2f	Cluster #49, fraction in ± 60 s of "egg out"	Cluster #49, fraction in t-SNE embedding	one-sided Fisher's exact test	1
Extended Data Fig. 2f	Cluster #50, fraction in ± 60 s of "egg out"	Cluster #50, fraction in t-SNE embedding	one-sided Fisher's exact test	1
Extended Data Fig. 2f	Cluster #51, fraction in ± 60 s of "egg out"	Cluster #51, fraction in t-SNE embedding	one-sided Fisher's exact test	1
Extended Data Fig. 2f	Cluster #52, fraction in ± 60 s of "egg out"	Cluster #52, fraction in t-SNE embedding	one-sided Fisher's exact test	1
Extended Data Fig. 2f	Cluster #53, fraction in ± 60 s of "egg out"	Cluster #53, fraction in t-SNE embedding	one-sided Fisher's exact test	1
Extended Data Fig. 5a	GAL4-only	ATB-1>Kir2.1	two-sided Wilcoxon rank sum test	$4.80x10^{-4}$
Extended Data Fig. 5a	UAS-only	ATB-1>Kir2.1	two-sided Wilcoxon rank sum test	0.0017
Extended Data Fig. 5b	GAL4-only	ATB-1>Kir2.1	two-sided Wilcoxon rank sum test	$4.11x10^{-4}$
Extended Data Fig. 5b	UAS-only	ATB-1>Kir2.1	two-sided Wilcoxon rank sum test	0.0455
Extended Data Fig. 5c	GAL4-only	ATB-2>Kir2.1	two-sided Wilcoxon rank sum test	$1.15x10^{-8}$
Extended Data Fig. 5c	UAS-only	ATB-2>Kir2.1	two-sided Wilcoxon rank sum test	$6.01x10^{-6}$
Extended Data Fig. 5d	GAL4-only	ATB-2>Kir2.1	two-sided Wilcoxon rank sum test	0.4644
Extended Data Fig. 5d	UAS-only	ATB-2>Kir2.1	two-sided Wilcoxon rank sum test	$6.46x10^{-4}$
Extended Data Fig. 6b	ATB-1 > UAS>>Kir2.1	ATB-1 > UAS>>Kir2.1; Otd-nls:FLP	two-sided Wilcoxon rank sum test	$9.26x10^{-9}$

Extended Data Fig. 6b	empty-splitGAL4> (FRT.mCherry)Kir2.1; Otd-nls:FLP	ATB-1 > (FRT.mCherry)Kir2.1; Otd-nls:FLP	two-sided Wilcoxon rank sum test	0.0302
Extended Data Fig. 6c	ATB-1 > (FRT.mCherry)Kir2.1;	ATB-1 > (FRT.mCherry)Kir2.1; Otd-nls:FLP	two-sided Wilcoxon rank sum test	1.53×10^{-6}
Extended Data Fig. 6c	empty-splitGAL4> (FRT.mCherry)Kir2.1; Otd-nls:FLP	ATB-1 > (FRT.mCherry)Kir2.1; Otd-nls:FLP	two-sided Wilcoxon rank sum test	0.0012
Extended Data Fig. 8a	"No light" control	Photo-stimulation	two-sided Wilcoxon rank sum test	0.0575
Extended Data Fig. 8d	"No light" control	2.5s stim, persist	two-sided Fisher's exact test	1
Extended Data Fig. 8d	"No light" control	2.5s stim, stop	two-sided Fisher's exact test	<0.0001
Extended Data Fig. 8d	"No light" control	5s stim, persist	two-sided Fisher's exact test	1
Extended Data Fig. 8d	"No light" control	5s stim, stop	two-sided Fisher's exact test	<0.0001
Extended Data Fig. 8d	"No light" control	20s stim, persist	two-sided Fisher's exact test	1
Extended Data Fig. 8d	"No light" control	20s stim, stop	two-sided Fisher's exact test	<0.0001
Extended Data Fig. 8d	2.5s stim, persist	2.5s stim, stop	two-sided Fisher's exact test	<0.0001
Extended Data Fig. 8d	5s stim, persist	5s stim, stop	two-sided Fisher's exact test	<0.0001
Extended Data Fig. 8d	20s stim, persist	20s stim, stop	two-sided Fisher's exact test	0.0667
Supplementary Fig. 5a	GAL4-only	PU-2>Kir2.1	two-sided Wilcoxon rank sum test	6.34×10^{-9}
Supplementary Fig. 5a	UAS-only	PU-2>Kir2.1	two-sided Wilcoxon rank sum test	3.23×10^{-8}
Supplementary Fig. 5b	GAL4-only	PU-2>Kir2.1	two-sided Wilcoxon rank sum test	4.99×10^{-5}
Supplementary Fig. 5b	UAS-only	PU-2>Kir2.1	two-sided Wilcoxon rank sum test	2.23×10^{-5}
Supplementary Fig. 5c	GAL4-only	PU-1>Kir2.1	two-sided Fisher's exact test	1
Supplementary Fig. 5c	UAS-only	PU-1>Kir2.1	two-sided Fisher's exact test	0.1296
Supplementary Fig. 5d	GAL4-only	PU-1>Kir2.1	two-sided Wilcoxon rank sum test	1.47×10^{-4}
Supplementary Fig. 5d	UAS-only	PU-1>Kir2.1	two-sided Wilcoxon rank sum test	0.0018
Supplementary Fig. 6	empty-splitGAL4> GtACR1, light OFF	empty-splitGAL4> GtACR1, light ON	two-sided Wilcoxon rank sum test	1
Supplementary Fig. 6	CMU-1-control, light OFF	CMU-1-control, light ON	two-sided Wilcoxon rank sum test	0.0546
Supplementary Fig. 6	CMU-1>GtACR1, light OFF	CMU-1>GtACR1, light ON	two-sided Wilcoxon rank sum test	0.0025
Supplementary Fig. 6	CMU-2-control, light OFF	CMU-2-control, light ON	two-sided Wilcoxon rank sum test	0.4055
Supplementary Fig. 6	CMU-2>GtACR1, light OFF	CMU-2>GtACR1, light ON	two-sided Wilcoxon rank sum test	0.0019

Supplementary Table 8 | Significance (*p*-value) of mapping between t-SNE clusters and human labels.

one-sided permutation test

	walk	PE	bend	burrow	expel	drag	groom	bg
**Cluster #0**	1	1	1	1	1	1	1	0
**Cluster #1**	0.0007	0.0884	0.3356	1	1	0.6001	0.8859	0.5
**Cluster #2**	1	1	1	1	1	1	0	0.5
**Cluster #3**	0.5	0	0	1	1	0.5	0.583	0.5
**Cluster #4**	1	0.9866	0.8674	1	1	0.9987	0	0.5
**Cluster #5**	1	1	1	1	1	1	0	0.5
**Cluster #6**	1	1	0.9987	1	1	0.9028	0	0.5
**Cluster #7**	1	0.9927	1	1	1	1	0	0.4794
**Cluster #8**	1	0.9947	0	0	0.6587	0.0011	0.3584	0.5
**Cluster #9**	0.9307	0.9896	0.8152	0.9127	1	0.9737	0.5052	0
**Cluster #10**	1	0.9996	1	1	1	0.9997	0	0.5
**Cluster #11**	0.989	0.4655	0	0.7182	1	0.6778	0.6103	0.5
**Cluster #12**	1	0.9245	0.9845	1	1	0.9998	0.5	0
**Cluster #13**	1	1	1	1	1	1	0.201	0.0579
**Cluster #14**	0.4784	0	0.0023	0.9865	1	0.6139	0.8694	0.5
**Cluster #15**	1	0.9981	0.9151	0.984	1	0.9631	0	0.5
**Cluster #16**	1	0.9995	1	1	1	0.9686	0.8123	0
**Cluster #17**	0.9286	0	0.7026	1	1	0.9986	0.5858	0.0105
**Cluster #18**	1	0.9997	0.8656	0.8739	1	0.8792	0	0.5
**Cluster #19**	1	1	0.9999	1	0.3978	0.5	0.5	0
**Cluster #20**	1	1	0.0307	0	0.1313	0.5006	0.5618	0.5
**Cluster #21**	0.9386	0	0.7991	0.8609	0.0836	0.4986	0.4999	0.0434
**Cluster #22**	0.999	0.7058	1	1	1	0.6731	0	0.5
**Cluster #23**	1	0.9955	1	1	1	0.9138	0.82	0
**Cluster #24**	1	0.9955	0.0375	0.0001	0.0008	0	1	0.5
**Cluster #25**	1	0.9996	0.2008	0	0	0.0374	0.8767	0.5
**Cluster #26**	1	1	1	1	1	1	0	0.5
**Cluster #27**	1	1	1	1	1	1	1	0
**Cluster #28**	1	0.9085	1	1	1	0.8246	0	0.4967
**Cluster #29**	1	1	0.9878	0	0	0.5687	0.6435	0.5
**Cluster #30**	1	0.0001	0.7993	1	1	1	0	0.4981
**Cluster #31**	1	0.9998	0.9508	0	0	0	0.4987	0.5
**Cluster #32**	1	1	1	1	1	0.5678	0.2527	0.0307
**Cluster #33**	1	0.9998	1	1	1	1	0.9935	0
**Cluster #34**	1	1	1	1	1	1	1	0
**Cluster #35**	1	0.862	0.5309	1	1	1	0.4308	0.0039
**Cluster #36**	1	0.5886	1	0.9782	1	1	0	0.4989
**Cluster #37**	1	1	1	1	1	1	1	0
**Cluster #38**	1	1	1	1	1	1	1	0
**Cluster #39**	1	1	1	1	1	1	0	0.5
**Cluster #40**	1	0.9974	1	0.7919	0.2318	0.6491	0.4347	0.0005
**Cluster #41**	1	0.867	1	1	1	1	0	0.4983
**Cluster #42**	1	0.9999	1	1	1	0.9771	0.939	0
**Cluster #43**	1	0	1	1	1	0.9983	0.5015	0
**Cluster #44**	1	0.999	1	0.79	0.3876	0.4372	0.8594	0
**Cluster #45**	1	0.9919	1	1	1	1	0	0.4998
**Cluster #46**	1	1	1	1	1	1	0.953	0
**Cluster #47**	1	1	1	1	1	1	0.9896	0
**Cluster #48**	1	1	1	1	1	1	0.636	0
**Cluster #49**	1	1	1	1	1	1	1	0.0001
**Cluster #50**	1	1	0.9969	1	1	0.7989	0.9987	0
**Cluster #51**	1	1	0.8057	0.9472	1	1	0.8524	0
**Cluster #52**	1	1	1	1	1	1	1	0
**Cluster #53**	1	1	1	0.6208	1	0.6842	0.5788	0.0051

Supplementary Methods

Unsupervised behavioral classification analysis. DeepLabCut (Mathis et al., *Nat Neurosci* 21, 1281–1289 (2018)) was used to track the following 18 key-points: two points on the head (the ocellus and proboscis tip), the posterior tip of the scutellum on the thorax, both wing tips, the joint between the femur and tibia on all six legs, the dorsal arch of the stripe on abdominal segment A5, the ventral-most edge of the stripes on abdominal segments A2 and A6 (per side; 4 points total), the tip of the ovipositor, and the egg, when visibly emerging from the ovipositor.

Given the challenges of resolving a consistent pose estimate from a fixed viewing perspective within a 3-dimensional environment (Günel et al., *eLife* 8, e48571 (2019)), this analysis was restricted to subsets of the data where the fly displayed an approximately lateral perspective relative to the camera for greater than 4 s (the first and last 2 s were discarded) (**Supplementary Fig. 3** and **Supplementary Video 2**). This was determined as those frames meeting the following criteria: the dorsal and ventral abdominal stripes, as well as the scutellum tip, were all visible, and the distance between the scutellum tip and the wing tips was greater than 70% of the maximum. By this criterion, 7.5 million suitable frames (~105 hours) were identified from 184 wild-type flies. Note that synchronous recordings from multiple cameras would be required to resolve a consistent, orientation-invariant pose-estimation model to implement the unsupervised behavioral classification analysis pipeline used here over contiguous video segments within 3-dimensional chambers.

Abdominal bend angle ('ba') was normalized to correct for angular changes that result from small deviations in perspective. Different perspectives were identified and grouped according to the angle formed between a line connecting the ocellus and scutellum and the chamber surface. Within each group, the mean and standard deviation of bend angles was used to determine the z-score.

Diverse frames were selected from each fly (Berman et al., *J. R. Soc. Interface*. 11, 20140672 (2014)), amounting to a total of 192,068 frames, which were then embedded into a two-dimensional representation using t-SNE (distance metric, standardized Euclidean distance; perplexity, 750). A subset of frames where the fly was not moving and exhibited a neutral posture were designated as "stationary" frames and were precluded from this analysis. This mapping was then convolved with a Gaussian (sigma = 1.1) and a watershed transform was performed to delineate 53 regions within the embedded space (**Extended Data Fig. 2b**). "Stationary" frames were assigned to cluster #0 in **Extended Data Fig. 2e**,**f**.

For the projection of egg-laying behavioral sequences onto the t-SNE embedding (**Extended Data Fig. 2c-f**), an automated analysis was used to identify 731 egg-laying events from 74 flies (a cohort of 52 flies were filmed in chambers that were 50% longer along the lateral-viewing axis of the substrate). This analysis identified peaks in the DLC egg-emergence prediction, derived a spatial mask from the deposited egg surrounding a given peak, and finally determined the egg-laying event as the time at which this mask was >95% occupied by the egg. The cluster identity of individual frames from the ± 60 s of these egg-laying events was determined as the mode cluster identity of the 100 k-nearest neighboring frames in the original t-SNE embedding. To identify clusters with enriched expression surrounding egg laying, the total fraction

of frames assigned a given cluster within this 120 second window was compared to its fraction within the original t-SNE embedding (**Extended Data Fig. 2f**, top). For plotting the time course of expression (**Extended Data Fig. 2c**), the fraction of frames assigned to a given cluster relative to the total number of analyzed frames was calculated independently at each time point. The statistical significance of mappings between human labels and t-SNE clusters, and vice versa, was determined by comparison to a "chance" mapping distribution obtained by randomly shuffling the annotation data relative to the cluster identities for 10,000 permutations (**Extended Data Fig. 2e**,**f**).

Correspondence between the manual labels and unsupervised classifier was high, further validating the behavioral composition and sequential organization of egg-laying behavior described in Figure 1. 14 of the 53 t-SNE behavioral clusters were expressed significantly above chance and formed a temporal sequence in the 40 s surrounding egg deposition (**Extended Data Fig. 2a-d** and **Supplementary Video 3**). 10 of these 14 behaviors reliably map onto a single manually defined label ($p<0.001$, permutation test; **Extended Data Fig. 2d-f**). 3 of the remaining 4 behaviors mapped onto non-labeled frames and primarily consisted of stationary behaviors involving abdominal contortions likely related to ovulation (Vijayan et al., bioRxiv doi:10.1101/2021.09.23.461548 (2021)). Though reliable, mapping was not one-to-one: individual human labels typically mapped onto a small subset of t-SNE clusters and vice versa. This is an indication that finer scale behavioral sub-components were identified in the unsupervised behavioral classification analysis, and is a function of the Gaussian filter used to smooth the original t-SNE embedding. For example, t-SNE clusters associated with oscillating ovipositor pixel intensity ("w1ovi", "w2ovi") or egg emergence ("Pegg") were both reliably labeled as "burrow" (cluster #20 and #29, respectively), whereas clusters associated with various combinations of hind-leg movement ('T3') and high angular velocity of the abdomen ('velba') were reliably labeled as "groom" (clusters #4, #5, #6, #15, #18; **Extended Data Fig. 2d**). Note that "PE" reliably maps to t-SNE cluster #17 in **Extended Data Fig. 2f**, but that cluster #17 maps more reliably to "background" in **Extended Data Fig. 2e**. This apparent discrepancy is an artifact of the manual labeling criterion used for "PE" where only a single frame was labeled at the onset of a given proboscis extension event.
