# Supplementary material for: Flexible neural control of transition points within the egg-laying behavioral sequence in Drosophila
Source: Nat Neurosci. 2023 May 22;26(6):1054–67. doi: 10.1038/s41593-023-01332-5 (PMC10244180; doi:10.1038/s41593-023-01332-5)
Supplement: Supplementary file 2 — Reporting Summary [file 41593_2023_1332_MOESM2_ESM.pdf]

nature portfolio

Corresponding author(s): Richard Axel

Last updated by author(s): March 23, 2022

# Reporting Summary

Nature Portfolio wishes to improve the reproducibility of the work that we publish. This form provides structure for consistency and transparency in reporting. For further information on Nature Portfolio policies, see our Editorial Policies and the Editorial Policy Checklist.

## Statistics

For all statistical analyses, confirm that the following items are present in the figure legend, table legend, main text, or Methods section.

| n/a | Confirmed | |
|---|---|---|
| ☐ | ☒ | The exact sample size (*n*) for each experimental group/condition, given as a discrete number and unit of measurement |
| ☐ | ☒ | A statement on whether measurements were taken from distinct samples or whether the same sample was measured repeatedly |
| ☐ | ☒ | The statistical test(s) used AND whether they are one- or two-sided<br>*Only common tests should be described solely by name; describe more complex techniques in the Methods section.* |
| ☒ | ☐ | A description of all covariates tested |
| ☐ | ☒ | A description of any assumptions or corrections, such as tests of normality and adjustment for multiple comparisons |
| ☐ | ☒ | A full description of the statistical parameters including central tendency (e.g. means) or other basic estimates (e.g. regression coefficient) AND variation (e.g. standard deviation) or associated estimates of uncertainty (e.g. confidence intervals) |
| ☐ | ☒ | For null hypothesis testing, the test statistic (e.g. *F*, *t*, *r*) with confidence intervals, effect sizes, degrees of freedom and *P* value noted<br>*Give P values as exact values whenever suitable.* |
| ☒ | ☐ | For Bayesian analysis, information on the choice of priors and Markov chain Monte Carlo settings |
| ☒ | ☐ | For hierarchical and complex designs, identification of the appropriate level for tests and full reporting of outcomes |
| ☒ | ☐ | Estimates of effect sizes (e.g. Cohen's *d*, Pearson's *r*), indicating how they were calculated |

*Our web collection on statistics for biologists contains articles on many of the points above.*

## Software and code

Policy information about availability of computer code

| | |
|---|---|
| Data collection | Video data was collected using pylon Viewer 6.2 (Basler) and FlyCapture 2.0 SDK (Point Grey). 2-photon imaging data was collected using PrairieView 5.4 (Bruker). |
| Data analysis | Confocal images were analyzed in ImageJ (ver: 1.53f51). Two-photon ROI segmentation and unsupervised behavioral classification were based on models generated in DeepLabCut (ver: 2.2.1). Supervised behavioral classification was performed using DeepEthogram (ver: 0.1.4). Behavioral and imaging data were analyzed using custom MATLAB (R2019a) code, which we have made available for public use via GitHub. |

For manuscripts utilizing custom algorithms or software that are central to the research but not yet described in published literature, software must be made available to editors and reviewers. We strongly encourage code deposition in a community repository (e.g. GitHub). See the Nature Portfolio guidelines for submitting code & software for further information.

## Data

Policy information about availability of data

All manuscripts must include a data availability statement. This statement should provide the following information, where applicable:

- Accession codes, unique identifiers, or web links for publicly available datasets
- A description of any restrictions on data availability
- For clinical datasets or third party data, please ensure that the statement adheres to our policy

The data from this study can be accessed via GitHub and upon request. Trained pose-estimation models and supervised behavioral classifier available via Dropbox.

## Human research participants

Policy information about studies involving human research participants and Sex and Gender in Research.

| | |
|---|---|
| Reporting on sex and gender | N/A |
| Population characteristics | N/A |
| Recruitment | N/A |
| Ethics oversight | N/A |

Note that full information on the approval of the study protocol must also be provided in the manuscript.

# Field-specific reporting

Please select the one below that is the best fit for your research. If you are not sure, read the appropriate sections before making your selection.

☒ Life sciences ☐ Behavioural & social sciences ☐ Ecological, evolutionary & environmental sciences

For a reference copy of the document with all sections, see nature.com/documents/nr-reporting-summary-flat.pdf

# Life sciences study design

All studies must disclose on these points even when the disclosure is negative.

| | |
|---|---|
| Sample size | No statistical methods were used to pre-determine sample sizes but our sample sizes are comparable to the other functional and behavioral studies in Drosophila (e.g. Feng et al., 2014; Gou et al., 2014; Seeholzer et al., 2018; Bräcker et al., 2019; Wang et al., 2020; Zhang et al., 2020.) |
| Data exclusions | We did not exclude flies or data from analysis. |
| Replication | Each experiment presented in the manuscript was repeated in multiple animals, and the effects identified were consistent across animals. The specific number of replicates for each experiment is detailed on the figure and/or its corresponding legend entry. Furthermore, the main findings of the paper were confirmed by multiple complimentary experiments. A subset of 30 randomly selected 2-minute videos was re-annotated by a second human annotator and labeling agreement was above 95% across all behaviors. Analysis was performed with code that is available for public use via GitHub to promote replication. |
| Randomization | Flies were group housed separated by genotype, and females were randomly chosen for functional or behavioral experiments. |
| Blinding | The experimenter was not blind to fly genotype and/or experimental condition during behavioral data acquisition (Figures 1-4, 6-8) as this was not logistically possible. However, all behavioral annotations were performed blinded to these details. Furthermore, the subsequent analyses were automated and run the same way for all flies and so there was no opportunity for subjective influence on the outcome. The assessment of the depth and count of eggs laid (Figures 2-3, 6) was performed blinded to the genotype and/or experimental condition. For imaging experiments (Figures 5, 8), the ROIs corresponding to the relevant cell types were determined at the conclusion of the experiment and so the experimenter had no opportunity to influence the outcome. Furthermore, the analysis and identification of relevant behavioral events used to interpret the imaging data was entirely automated. The assessment of the fraction of flies that expelled an egg during optogenetic stimulation (Figure 8) was not performed in real-time; video data was later scored blind to the genotype and experimental condition. |

# Reporting for specific materials, systems and methods

We require information from authors about some types of materials, experimental systems and methods used in many studies. Here, indicate whether each material, system or method listed is relevant to your study. If you are not sure if a list item applies to your research, read the appropriate section before selecting a response.

## Materials & experimental systems

| n/a | Involved in the study |
|---|---|
| ☐ | ☒ Antibodies |
| ☒ | ☐ Eukaryotic cell lines |
| ☒ | ☐ Palaeontology and archaeology |
| ☐ | ☒ Animals and other organisms |
| ☒ | ☐ Clinical data |
| ☒ | ☐ Dual use research of concern |

## Methods

| n/a | Involved in the study |
|---|---|
| ☒ | ☐ ChIP-seq |
| ☒ | ☐ Flow cytometry |
| ☒ | ☐ MRI-based neuroimaging |

# Antibodies

Antibodies used

Mouse mAb anti-bruchpilot (nc82) (Developmental Studies Hybridoma Bank - nc82; RRID: AB_2314865)
Chicken anti-GFP (Aves Labs - GFP-1020; RRID: AB_10000240)
Rabbit anti-DsRed (Clontech - 632496; RRID: AB_10013483)
Rabbit anti-NOMPC (gift of Y.N. Jan; Zhang et al., Cell 162, 1391–1403 (2015))
Rabbit anti-dVGlut (gift of A. DiAntonio; Daniels et al., J Neurosci. 24, 10466-10474 (2004))
Alexa Fluor 633-conjugated goat anti-mouse IgG (Life Technologies - A21052; RRID: AB_141459)
Alexa Fluor 488-conjugated goat anti-chicken IgY (Life Technologies - A11039; RRID: AB_142924)
Alexa Fluor 488-conjugated goat anti-rabbit IgG (Life Technologies - A11008; RRID: AB_143165)
Alexa Fluor 633 phalloidin F-actin probe (Life Technologies - A22284)
Alexa Fluor 555-conjugated goat anti-rabbit IgG (Life Technologies - A27039; RRID: AB_2536100)

Validation

The antibodies and protocols were used and validated in numerous studies, including for example Hattori et al., Cell 169, 956-969 (2017), Zhang et al., Cell 162, 1391–1403 (2015), and Daniels et al., J Neurosci. 24, 10466-10474 (2004).

# Animals and other research organisms

Policy information about studies involving animals; ARRIVE guidelines recommended for reporting animal research, and Sex and Gender in Research

Laboratory animals

We used 3-20 day old female Drosophila melanogaster. Canton-S gifted from B.J. Dickson was used as a wild-type strain. All relevant genotypes with citations and sources are described in Supplementary Table 5.

Wild animals

This study did not involve wild animals.

Reporting on sex

All findings apply only to female Drosophila melanogaster.

Field-collected samples

This study did not involve samples collected in the field.

Ethics oversight

No ethical approval was required for work on Drosophila melanogaster.

Note that full information on the approval of the study protocol must also be provided in the manuscript.
